# Identification and analysis of seven effector protein families with different adaptive and evolutionary histories in plant-associated members of the Xanthomonadaceae

**DOI:** 10.1038/s41598-017-16325-1

**Published:** 2017-11-23

**Authors:** Renata de A. B. Assis, Lorraine Cristina Polloni, José S. L. Patané, Shalabh Thakur, Érica B. Felestrino, Julio Diaz-Caballero, Luciano Antonio Digiampietri, Luiz Ricardo Goulart, Nalvo F. Almeida, Rafael Nascimento, Abhaya M. Dandekar, Paulo A. Zaini, João C. Setubal, David S. Guttman, Leandro Marcio Moreira

**Affiliations:** 1 Center of Research in Biological Science, Federal University of Ouro Preto, Ouro Preto, MG Brazil; 2 Institute of Genetics and Biochemistry, Federal University of Uberlândia, Uberlândia, MG Brazil; 3 Departamento de Bioquímica, Instituto de Química, Universidade de São Paulo, São Paulo, SP Brazil; 4 Department of Cell & Systems Biology, University of Toronto, 25 Willcocks St., Toronto, Ontario, M5S 3B2 Canada; 5 Escola de Artes, Ciências e Humanidades, Universidade de São Paulo, São Paulo, SP Brazil; 6 School of Computing, Federal University of Mato Grosso do Sul, Mato Grosso do Sul, MS Brazil; 7 Department of Plant Sciences, University of California, Davis, CA USA; 8 Centre for the Analysis of Genome Evolution and Function, University of Toronto, 25 Willcocks St., Toronto, Ontario M5S 3B2 Canada; 9 Department of Biological Science, Institute of Exact and Biological Science, Federal University of Ouro Preto, Ouro Preto, MG Brazil

## Abstract

The Xanthomonadaceae family consists of species of non-pathogenic and pathogenic γ-proteobacteria that infect different hosts, including humans and plants. In this study, we performed a comparative analysis using 69 fully sequenced genomes belonging to this family, with a focus on identifying proteins enriched in phytopathogens that could explain the lifestyle and the ability to infect plants. Using a computational approach, we identified seven phytopathogen-enriched protein families putatively secreted by type II secretory system: PheA (*CM-sec*), LipA/LesA, VirK, and four families involved in N-glycan degradation, NixE, NixF, NixL, and FucA1. *In silico* and phylogenetic analyses of these protein families revealed they all have orthologs in other phytopathogenic or symbiotic bacteria, and are involved in the modulation and evasion of the immune system. As a proof of concept, we performed a biochemical characterization of LipA from Xac306 and verified that the mutant strain lost most of its lipase and esterase activities and displayed reduced virulence in citrus. Since this study includes closely related organisms with distinct lifestyles and highlights proteins directly related to adaptation inside plant tissues, novel approaches might use these proteins as biotechnological targets for disease control, and contribute to our understanding of the coevolution of plant-associated bacteria.

## Introduction

With the advancement of comparative genomics tools and the rapid increase in completely sequenced bacterial genomes, it became feasible to relate their genetic constitution to their lifestyle and interaction with compatible hosts^1,2^. Comparison among complete genomes of closely related strains with different lifestyles and virulence may elucidate not only mechanisms of infection and adaptation inside the host, but also identify genes responsible for induction of virulence and ability to infect different hosts^3,4^.

The Xanthomonadaceae family includes endophytes, phytopathogens, human pathogens, as well as soil-associated and marine bacteria. It contains at least 27 genera, including *Arenimonas*, *Kaistibacter*, *Luteimonas*, *Lysobacter*, *Pseudoxanthomonas*, *Rehaibacterium*, *Silanimonas*, *Stenotrophomonas*, *Thermomonas*, *Vulcaniibacterium*, *Xanthomonas*, and *Xylella* ^5^. As of January 10, 2017, 862 genomes from this family have been completely or partially sequenced^6^. However, this coverage is not uniform in terms of genera. In particular, *Stenotrophomonas*, *Pseudoxanthomonas*, *Xylella*, and *Xanthomonas* dominate the sequencing statistics for the Xanthomonadaceae family.

Members of the *Xanthomonas* genus propagate through injuries on leaves, stems, and fruits, or even through natural openings of their plant hosts, with the wind as the main dispersion agent^7^. In addition, most *Xanthomonas* species can occupy both mesophyllic and the vascular environments within their respective hosts, except *Xanthomonas albilineans*, which is the only xylem-limited species so far described in this genus^8^. A remarkable feature shared by bacteria of the *Xanthomonas* genus is the large number and diversity of type III secretion system (T3SS) effectors and transcription activator-like (TAL) type III effector proteins that regulate host gene expression by associating with promoters of plant genes^9^. Bacteria of the *Xylella* genus are non-flagellate and xylem-limited, transferred directly inside the host xylem vessels by insect vectors upon feeding^10^. The known genomes of *Xylella* species do not encode a type III secretory system (T3SS), but encode type I and type II secretory systems for the export of exoenzymes and other proteins, which allow colonization within the plant xylem^11–13^. Biofilm formation is important for *Xylella* species survival in unstable environments with high turbulence, pressure oscillation, and limited nutrient availability, such as the xylem vessels and insect foreguts^11^. Bacteria of the genus *Stenotrophomonas* are opportunistic human pathogens, plant endophytes, or rhizosphere- and river sediment-associated^14–16^. Members of the *Pseudoxanthomonas* genus have been found in soil contaminated with hydrocarbons^17^, bioreactor compost-feedstock enrichment cultures^18^, and leafy wood soil^19^. Interestingly while *Xanthomonas* and *Xylella* have many members that are phytopathogens, this lifestyle has not been described for any *Stenotrophomonas* and *Pseudoxanthomonas*.

As aforementioned, the mechanisms for propagation, colonization, and induction of virulence are distinct among *Xanthomonas* and *Xylella* species. Other members of the Xanthomonadaceae family present additional diverse aspects in terms of plant-related lifestyles. Previous comparative genomics work by our group on *Xylella* and *Xanthomonas* genomes has shown that the diversity in plant hosts, and in distinct niches within hosts, whose pathologies are phenotypically distinct, can be related to differences in genomic content^20–22^. This work can thus be seen as an expansion of those prior studies, aiming to cover more members of the Xanthomonadaceae with fully sequenced genomes. By expanding the phylogenetic scope we were able to amplify the diversity in plant-associated lifestyles while retaining genomic relatedness, a combination that offers an excellent opportunity to deepen our understanding of bacteria-plant interactions.

## Results

**Figure 1:**
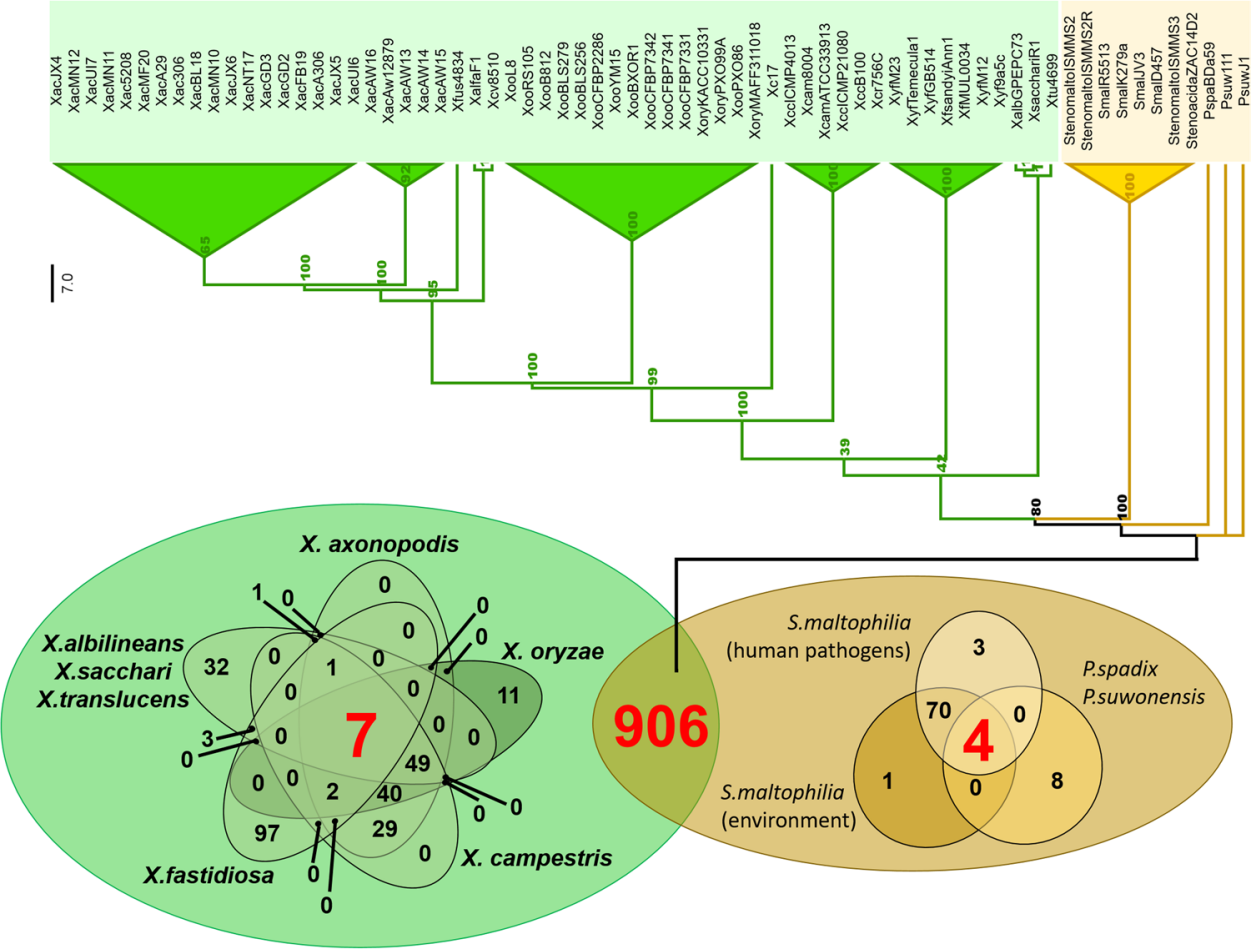
Comparative genomics of the 69 selected strains. Venn diagrams demonstrating core genome in phytopathogens, non-phytopathogens, and involving all analyzed strains. The phylogenomic tree was based on the core genome. The numbers in nodes represent bootstrap values and the branch lengths are not proportional to evolution time.

Sixty-nine completely sequenced genomes were selected for our comparative study (Table S1 – Supporting Information). Fifty-one belong to the *Xanthomonas* genus and seven to the *Xylella* genus, and all of the corresponding organisms are phytopathogens, except *Xanthomonas sacchari* strain R1, which was isolated from asymptomatic rice seeds (Fig. S1 and Table S1 – Supporting Information). Eight genomes from the *Stenotrophomonas* genus were included. Five of these are classified as human opportunistic pathogens (strains ISMMS2, ISMMS2R, ISMMS3, D457, K279a), one as endophyte (strain R5513), one as rhizosphere-associated (strain JV3), and one as river sediment-associated (strain ZAC14D2). Three genomes belonging to the genus *Pseudoxanthomonas* were also included. One is from a species with metabolic potential for xenobiotic degradation (strain BDa59), and the other two (strains 11-1 and J1) were isolated from a bioreactor and leafy wood soil, respectively. A synthesis of current knowledge regarding the species in this study is presented in Fig. S1. A phylogenomic profile based on the core protein families of the 69 species analyzed is shown in Fig. 1.

The basic question that we tried to answer with this genome set was whether they contain protein-coding gene families that could be used to characterize Xanthomonadaceae plant pathogens. We answer this question in the affirmative, presenting in what follows a set of seven protein families containing 420 members in total that were present in all 58 phytopathogenic strains analyzed and absent in all others (Table S2 – Supporting Information). The only exception was *Xanthomonas sacchari* R1, which has genes encoding all seven protein families, but has been isolated from a nonsymptomatic host^23^. A protein family in our study was defined operationally as a group of protein-coding genes clustered by OrthoMCL, with the added manual curation step of including additional genes by inspecting the results of a tBLASTN search (see Methods).

The seven protein families are the following: (1) lipase/esterase (LipA/LesA); (2) secreted chorismate mutase; (3) glycosyl hydrolase (NixF); (4) beta-galactosidase (NixL); two (5 and 6) alpha-L-fucosidase (NixE and FucA1); and (7) VirK protein. Various features of these seven protein families are shown in Table 1, including information regarding presence of a signal peptide, cellular localization, domain analysis (Fig. S3 – Supporting Information), and putative protein-protein interaction results. We now interpret the biological meaning and implications of this general result, focusing on each protein family in turn.

**Table 1:** Characterization of seven plant-associated exclusive proteins families.

Old locus tag	+ XAC0435	+ XAC0501	+ XAC1306	+ XAC3072	+ XAC3073	+ XAC3084	−XAC3647
**New locus tag**	XAC_RS02280	XAC_RS02605	XAC_RS06675	XAC_RS15590	XAC_RS15595	XAC_RS15650	XAC_RS18440
**Accession** ^**a**^	WP_003486419	WP_015462810	WP_011050833	WP_011052017	WP_015472473	WP_005924753	WP_003484013
**Gene name** ^**b**^	*virK*	*lipA/lesA*	*fucA1*	*nixE*	*nixF*	*nixL*	*pheA* (CMs)
**Product** ^**b**^	VirK protein	Secreted lipase	Alpha-L-fucosidase	Alpha-L-fucosidase	Glycosyl hydrolase	Beta-galactosidase	Secreted chorismate mutase
**COG**	----	COG0412	COG3669	COG3669	COG3858	COG1874	COG1605
**Start position** ^**b**^	518070	587832	1503221	3595544	3597245	3620253	4326854
**End position** ^**b**^	518501	588989	1505149	3597229	3598300	3622094	4326285
**Gene Onthology** ^**c**^	----	MF – 0004806	MF – 0004560	MF – 0004560	MF – 0004568	MF – 0004565	MF – 0004106
		BP – 0016042	BP – 0005975	BP – 0006004	BP – 0005975	BP – 0005975	BP – 0046417
		----	----	----	BP – 0006032	----	----
**Protein ID** ^**b**^	AAM35326	AAM35390	AAM36177	AAM37917	AAM37918	AAM37929	AAM38490
**Size (aa)** ^**b**^	143	421	642	561	351	613	189
**EC number** ^**b**^	----	----	3.2.1.51	3.2.1.51	----	3.2.1.23	5.4.99.5
**Domains prediction** ^**d**^ **(position)**	SP (1–22)	Abhydrolase_6 (54–366)	SP (1–23)	SP (1–40)	SP (1–21)	SP (1–24)	SP (1–30)
	VirK (25–121)	Abhydrolase_5 (88–356)	Alpha_L_fucos (39–395)	Alpha_L_fucos (87–509)	Glyco_18 (24–338)	Glyco_hydro_35 (38–357)	CM_2 (29–107)
	----	LIP (90–211)	F5_F8_type_C (502–633)	----	----	Glyco_hydro_42 (53–207)	----
**Cell location** ^**e**^	Unknown	Unknown	Unknown	Unknown	Unknown	Unknown	P (9.76)
**Genome Island** ^**f**^	N	N	N	N	N	N	N
**Signal peptide (size)** ^**g**^	Y (1–22)	Y (1–35)	Y (1–23)	Y (1–40)	Y (1–21)	Y (1–23)	Y (1–30)
**Uniprot**	Q8PQ93	Q8PQ30	Q8PMW9	Q8PI26	Q8PI25	Q8PI14	Q8PGG9
**Protein-protein interactions** ^**h**^	AvrXacE1	AmpE	NahA	β-mannosidase	NahA	Bga (2x)	TyrA
	Cellulase (2x)	ComL	BgaΨ(2x)	Glycosyl hydrolase	FucA1	BgaΨ	AroC
	AvrXacE2	RluD	Bga	Hp (XAC3073)	β-mannosidase	Dgd	P-protein
	HrcR	GumE	Hp (XAC1774)	----	Glycosyl hydrolase	Glycosyl hydrolase	TrpG
	CutC	Ketosynthase	Hp (XAC3083)	----	TonB	α-xylosidase	Hp (XAC1130)
	VirJ (2x)	Hp (XAC0904)	Hp (XAC3089)	----	TonB like	----	----
	XcsH	Hp (XAC0500)	----	----	Membrane protein	----	----
	Hp (XAC0434)	Hp (XAC3216)	----	----	TBDR	----	----
	----	Hp (XAC1183)	----	----	Hp (XAC3703)	----	----
**Protein-chemical Interactions** ^**h**^	----	Octyl glucoside	Glucose	Fucose	----	Glucose	Beta-NAD
		----	Chitin	Hydroxyl r.		Lactose	Prephenate
		----	Fucose	Methanol		Melibiose	P-hydroxyp.lpy.
		----	----	f-uf		Hydroxyl r.	Phenylpyruvic.
		----	----	ZWZ		----	Chorismate
		----	----	CTK8J7674		----	----
**HGT or PI** ^**f**^	0	0	0	0	0	0	0

a – According to NCBI^6^; b – According to KEGG^84^; c – According to UniProtKB-EC and Interpro^85^; d – According to SMART^86^; e – According to PFAM^87^; f – According to Island Viewer^88^; g – According to SignalP, Phobius, and TatP^89–91^; h – According to STITCH 4.0^92^. “+” – Plus strand; “−” – Minus strand; MF – Molecular function; BP – Biological process; SP – Signal peptide; Y – Yes; N – No; PI – Pathogenicity island.

### Family 1: Lipase/esterase, virulence inducer and plant immunity modulator

Lipases are a broad group of lipolytic enzymes divided into eight families comprising six subfamilies^24,25^. They belong to the superfamily of alpha/beta hydrolases together with other enzymes such as esterases, acetylcholinesterases, cutinases, carboxylesterases and epoxide hydrolases. Despite differences in sequence and function, members of this superfamily possess a conserved alpha/beta fold and pentapeptide motif. According to the amino acid composition of the catalytic triad, there are three classes of alpha/beta hydrolases: GGGX-, GX-, and the Y-class^26^. The GGGX class includes carboxylesterases and short chain length specific lipases that encompass LipA orthologs^26^. All *Xanthomonas* strains studied here have one copy of LipA, whereas all *Xylella* strains have three copies, except for strain M12, which has two copies. Other genes assigned with the lipase or esterase function are found in *Xanthomonas* genomes; however, these other genes are not orthologous to the family described in this section according to our analysis, since *lipA/lesA* combine both lipase and esterase and cleave exclusively small chain fatty acids^27^, besides other functional parameters (Table S3 – Supporting Information)^28,29^.

**Figure 2:**
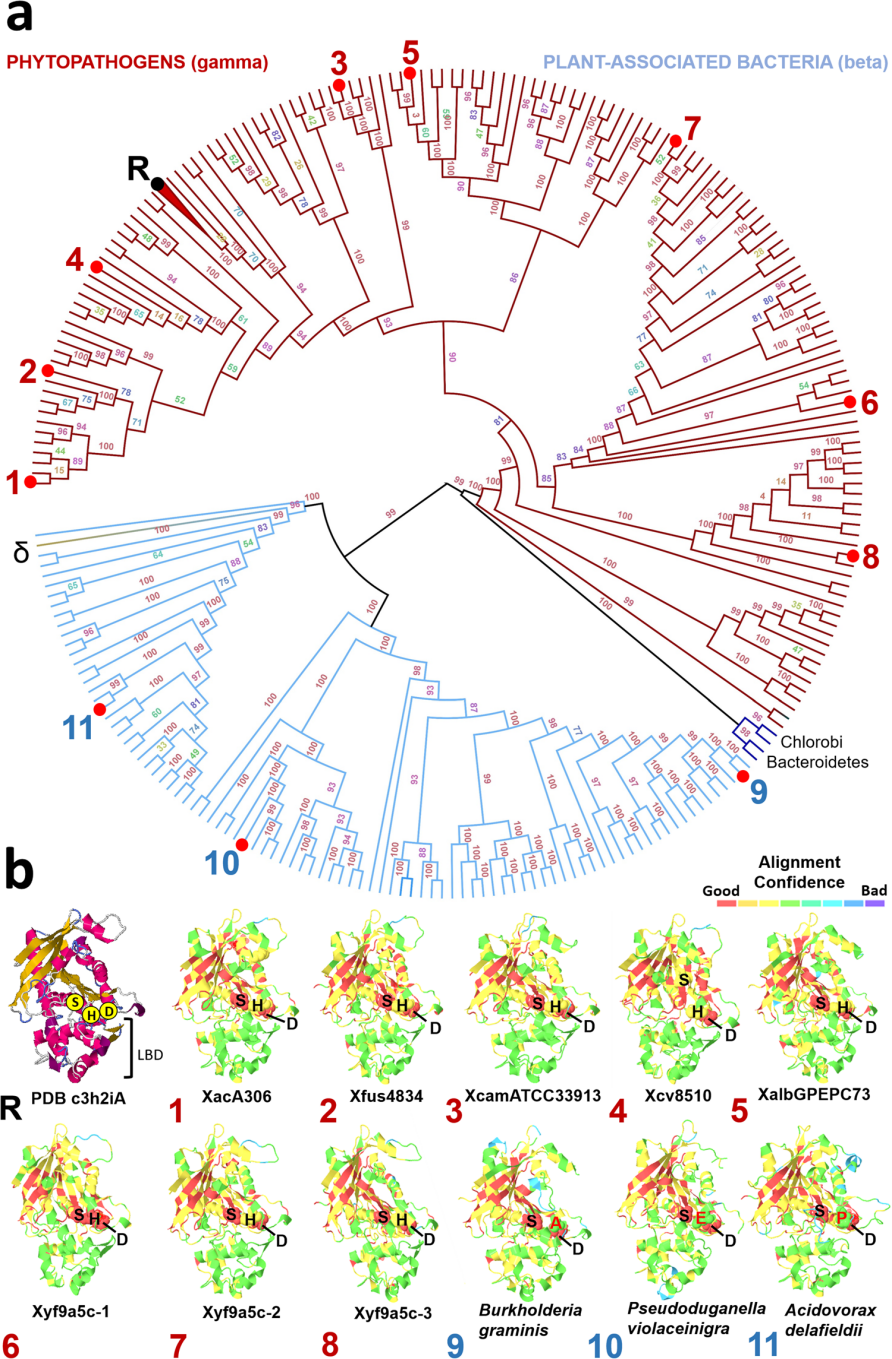
Phylogenetic and structural analysis of secreted lipase. (**a**) Highlighted in red are γ-proteobacteria (all phytopathogens) whereas blue highlights β-proteobacteria, all associated with soil or plant roots. The numbers associated with nodes represent bootstrap values at the color gradient. The numbers surrounding the phylogeny represent models proposed in “b” taken from the structure of the reference protein (R) obtained from *X. oryzae* using Protein Data Bank (PDB – c3h2Ia)^93^. (**b**) Structural profile of LipA orthologs from *X. oryzae* (R) highlighting the canonical residues directly involved in substrate catalysis (S176, D336 and H377). The putative protein structures (1 to 11) were obtained using Phyre2^82^. The alignment confidence degree varied from good (red) to bad (blue) in each structure, with the lowest conservation in the region associated with ligand-binding domain (LBD). Check for details at Fig. S9 – Supporting Information.

**Figure 3:**
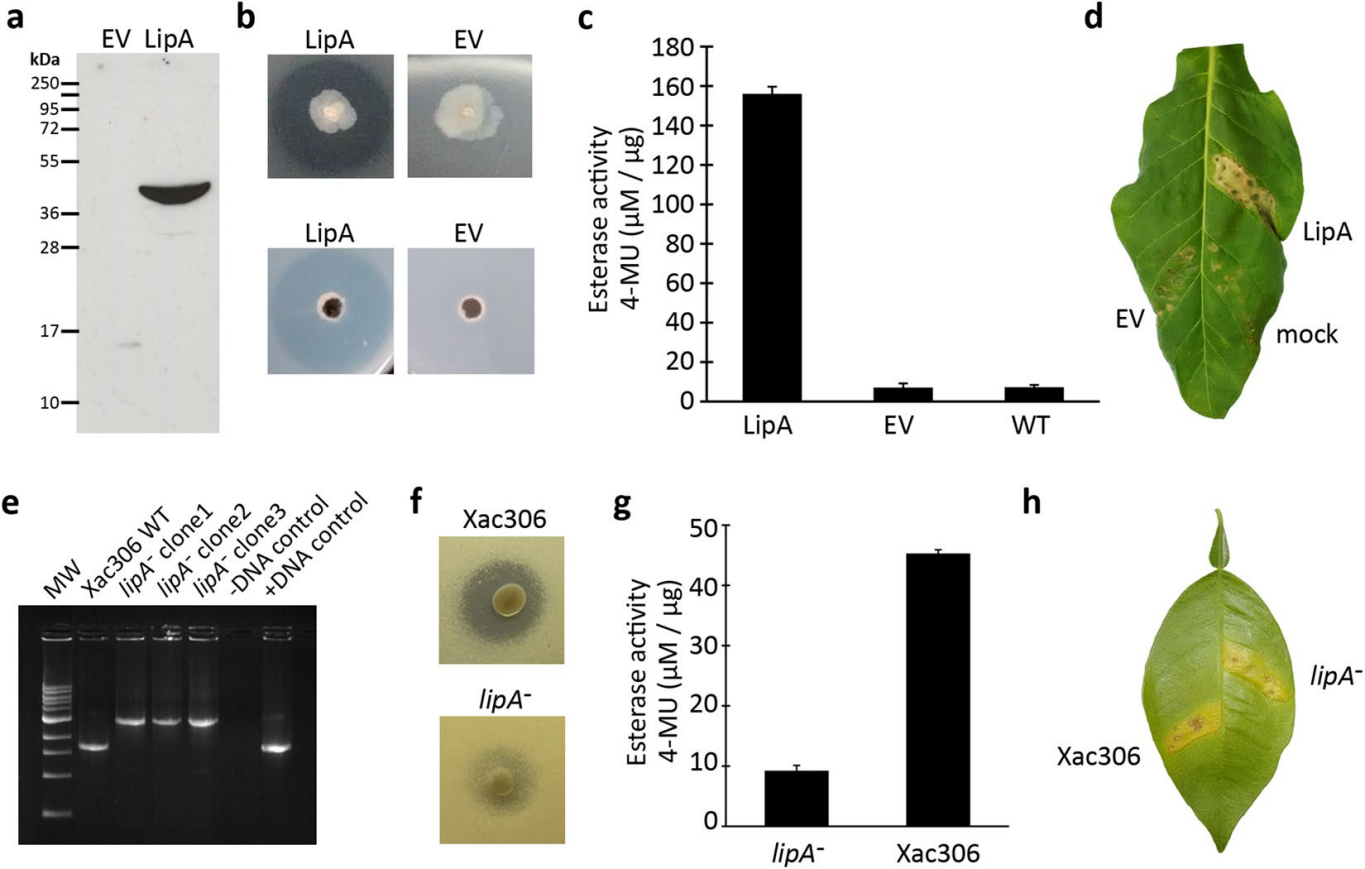
Characterization of LipA from *Xanthomonas axonopodis* pv. *citri* strain 306 pathotype A. Representative results of (**a**) western-blot of the protein encoded by XAC_RS02605 expressed in *E. coli* DH5α. (**b**) Lipase activity around a colony of the transformed *E. coli* on solid LB medium containing tributyrin, whereas transformant with an empty vector (EV) did not show any activity (n = 3). (**c**) Esterase activity of secreted extracts measured by fluorescence emitted by degradation of 4-methylumbelliferone (MUB) (n = 4). (**d**) Hypersensitive response on tobacco leaf infiltrated with cell extracts of *E. coli* transformed with an empty vector (EV) or containing the XAC_RS02605 cassette (n = 3). (**e**) Confirmation of disruption of *lipA* in Xac306 by PCR amplification. (**f**) Lipase activity around Xac306 colonies (n = 3). (**g**) Esterase activity of secreted Xac306 extracts (n = 4). (**h**) Virulence assay on citrus leaf by infiltration of wild type Xac306 and mutant with inactivated *lipA*. See Methods for more details on assays.

We investigated the presence of genes coding for LipA proteins (known as LesA in *Xylella* spp) in bacteria other than the 69 analyzed here. Interestingly we found that this gene is present in β- and other γ-proteobacteria, but only in plant-associated bacteria (Fig. 2a). This result indicates that this lipase/esterase may be fundamental not only for phytopathogenicity, but also for the association of bacteria with plants. Additional evidence for this hypothesis comes from the fact this lipase/esterase is absent in Xanthomonadaceae bacteria that are not phytopathogens, or indeed in any other members of the γ-proteobacteria family, according to our search results used to construct Fig. 2. The first hit from a non-plant associated bacteria is from *Thalassolituus sp. HI0120*, with an amino acid identity of only 40%; this protein product does not have the conserved catalytic triad. Although this ortholog from *Thalassolittus* has not been characterized biochemically, the substitution of the histidine within the catalytic triad suggests that this protein would have distinct biochemical properties^30^. Moreover, a structural profile of LipA orthologs from *X. oryzae* indicates that only γ-proteobacteria have genes that code for proteins with the same putative structure topology, with maintenance of the canonical residues directly involved in substrate catalysis (S176, D336 and H377) (Fig. 2b and Table S4 – Supporting Information). In the β-proteobacteria *B. graminis, P. violaceinigra*, and *A. delafieldii* the canonical residue H in the respective orthologs is replaced by A, E, and P, respectively. Whether this alters any of the enzymatic properties in this orthologs remains to be evaluated. Additionally, a biochemical characterization of LipA from Xac306 enabled us to verify its importance to virulence. When expressed in *E. coli*, the enzyme conferred lipase and esterase activity (Fig. 3a–c), and the cell extract was able to cause a hypersensitive response in tobacco leaves (Fig. 3d), while a Xac306 deletion-mutant for *lipA* lost most of its lipase and esterase activities (Fig. 3e–g) and displayed reduced virulence in citrus (Fig. 3h).

### Family 2: Secreted chorismate mutase, a key enzyme in modulation of the plant immune response

All genomes studied here have chorismate mutase (CM); however, non-phytopathogenic species have only one gene with this annotation while phytopathogens have two. The extra gene in phytopathogens is markedly distinct from the other gene, and despite being generally annotated as chorismate mutase, our analysis clearly shows that they belong to two distinct protein families that share a conserved domain. Firstly, the gene present only in phytopathogens averages 576 bp in length, encoding only the CM domain, whereas the other gene present in all taxa is 1,181 bp long, encompassing two other domains besides the CM domain. Secondly, we found that only those genes in the phytopathogens have a signal peptide (Fig. S5a–c – Supporting Information), suggesting that the encoded proteins are secreted. Based on these results we designated the phytopathogen-specific CM gene (and its respective orthologs) as *CM-sec* and the gene with wide distribution (and its respective orthologs) as *CM-nonsec*.

The protein coded by *CM-nonsec* has three domains: an N-terminal chorismate mutase (CM_2 PF01817), an internal prephenate dehydratase (PDT PF00800), and a C-terminal Phe regulatory (ACT PF01842) domain (Fig. S6a – Supporting Information), whereas the protein coded by *CM-sec* has only a chorismate mutase domain (CM_2 PF01817), which comprises almost the entire length of the protein (Fig. S6b and S7 – Supporting Information).

If CM-sec is secreted, what would be its function? It is known that the secretion of CM by the fungus *Ustilago maydis* reduces Trp synthesis, which modulates plant metabolism by reducing synthesis of defense compounds^31^. Based on this functional analogy, we performed a detailed *in silico* analysis of the CM-sec three-dimensional (3D) structure. The hit in the structural analysis was from *Burkholderia thailandensis* (PDB 4oj7.1.A) with 98.2% coverage and 39.74% identity, confirming the typical CM domain fold (Fig. S6b – Supporting Information). In addition, quality of conformation analysis between these two sequences indicates that all α-helices have a predicted quality higher than 80%. Furthermore, ten structural parameters were analyzed for this topology and only “relative conservation” shows low values, corroborating the alignment data and the coverage (Fig. S7 – Supporting Information), including conserved catalytic residues and residues involved in pocket conformation (Fig. S6c – Supporting Information).

**Figure 4:**
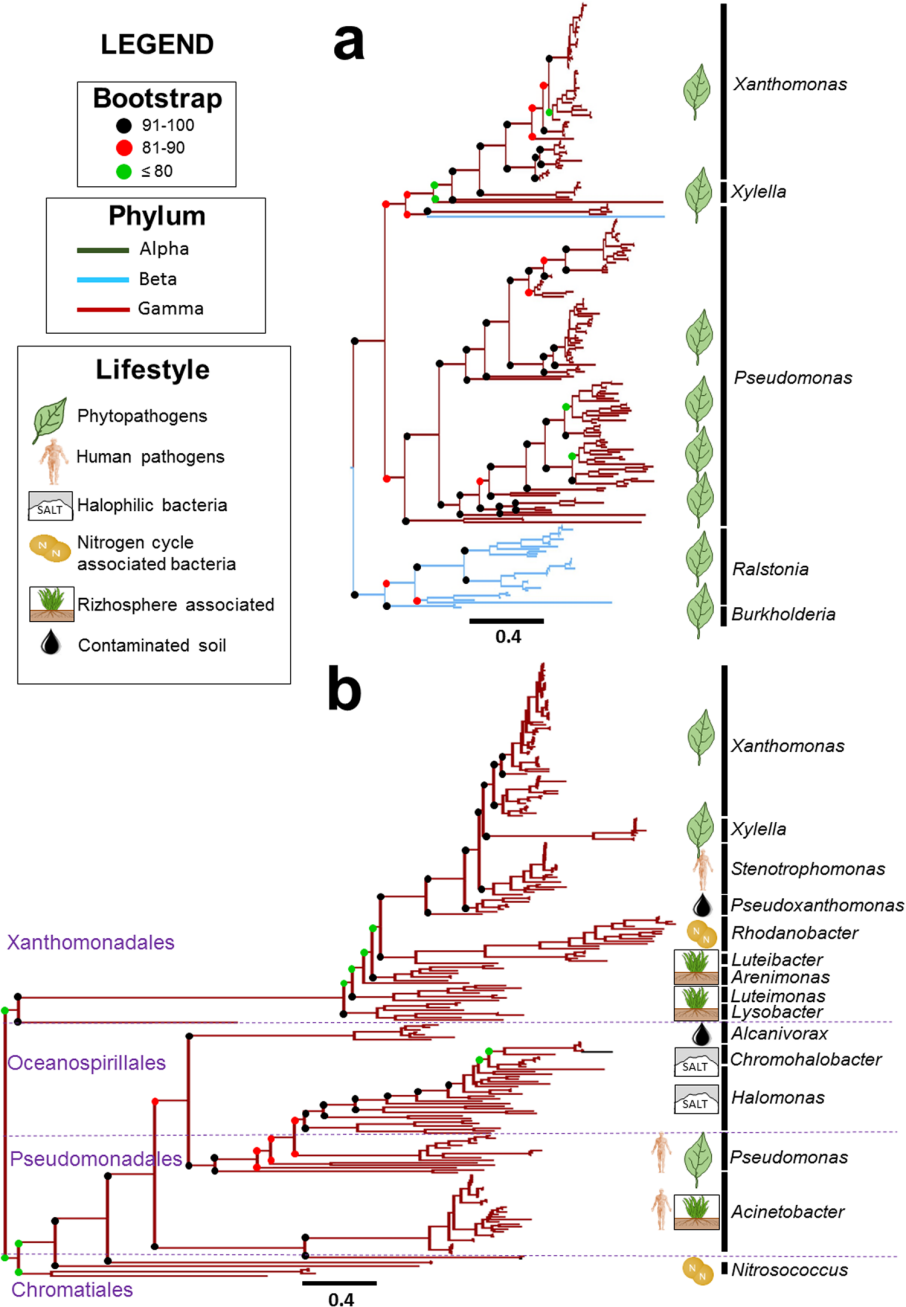
Phylogeny of *CM-sec* (**a**) and *CM-nonsec* (**b**). Both analysis utilized the first 250 comparison hits from Blastp, standardizing bars to 0.4 residues. It was noticed that for *CM-sec* the majority of analyzed organisms are plant-associated organisms (check for details at Fig. S10 and S11 – Supporting Information) which are found within three different classes (α, β and γ-proteobacterias), while for *CM-nonsec* all the analyzed microorganisms are introduced in γ-proteobacteria class maintaining the structural pattern of order belonging to this class. In addition, it is possible to observe that frequency of non-synonymous mutations is smaller in *CM-sec* indicating that this copy was more conserved during the evolution of the species.

We reconstructed the phylogenies for the 250 best Blastp hits for both *CM-sec* and *CM-nonsec*, using as queries the Xac306 versions. In the *CM-sec* tree 89% of the orthologs belong to plant-associated bacterial strains (Fig. 4a,b), from different classes (α, β, and γ-proteobacteria). The *CM-nonsec* tree follows the expected distribution of species within the γ-proteobacteria subdivisions. The branch lengths of the *CM-sec* tree are relatively small compared to those of the *CM-nonsec* tree, suggesting more evolutionary conservation for *CM-sec* genes.

### Families 3, 4, 5, and 6: N-glycan degradation enzymes, an intricate mechanism of plant immune system depletion

**Figure 5:**
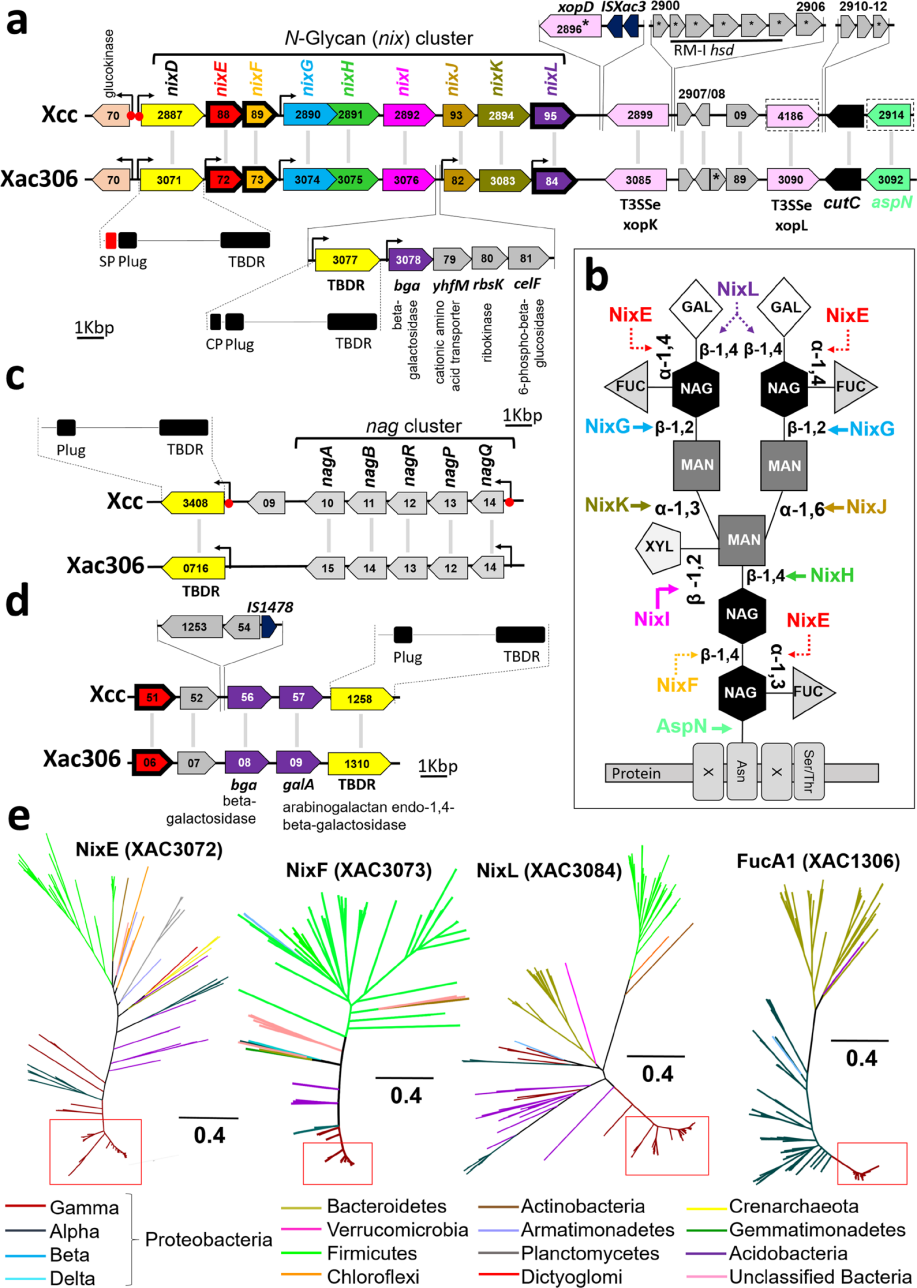
Functional and syntenic analysis of genes associated with N-glycans degradation. (**a**) Composition and structural organization of *nix* genes in Xcc and Xac306. Each of the individual colors of genes in the cluster corresponds to the respective site of catalysis shown for the N-glycan metabolism in Fig. 5b (adapted from Boulanger, *et al*.^48^). Three protein families from our study are encoded by genes included in this cluster and are circled in bold (NixE, NixF, and NixL). For Xac306 a set of genes with correlated functions between *nixI* and *nixJ* are highlighted. The region downstream of the cluster has structural variations among the investigated organisms. (**b**) Composition of an N-glycan with the respective enzymes associated with its degradation. The colors of the proteins identifiers and their catalysis follow the color pattern shown in Fig. 5a and d. The dotted lines refer to the catalysis of the phytopathogen-enriched proteins. (**c**) Structural organization of *nag* genes. (**d**) Composition and structural organization of putative genes associated with N-glycan degradation in Xcc and Xac306. The gene coding the FucA1 family specific to phytopathogens are circled in bold. (**e**) NixE, NixF, NixL, and FucA1 phylogenies. The red squares highlight positions occupied by *Xanthomonas* and *Xylella*. The clade composition of NixE, NixF, and NixL are similar among each other and distinct when compared to FucA1 because these enzymes encoded genes arranged in the same genomic region that compose the *nix* cluster. Check for details at Figs S12–S15 – Supporting Information.

The next four families that we have found are glycosyl hydrolase (NixF), beta-galactosidase (NixL), and two alpha-L-fucosidases (NixE and FucA1). These four families are associated with N-glycan degradation (Fig. 5)^32^. According to Boulanger, *et al*.^32^ and Dupoiron, *et al*.^33^, Xcc has two gene clusters associated with degradation (*nix*) and internalization of N-glycans and associated monomers (*nag*). Among the *nix* genes we find *nixE*, *nixF*, and *nixL*, coding for alpha-L-fucosidase, glycosyl hydrolase, and beta-galactosidase, respectively. Alpha-L-fucosidase catalyzes the α-1,4-glycosidic bond between L-fucose (FUC) and N-acetyl-glycosamine (NAG), glycosyl hydrolase hydrolyzes the β-1,4-glycosidic bound between NAG, and beta-galactosidase hydrolyzes the β-1,4-glycosidic between NAG and galactose (GAL) (Fig. 5b).

Comparative analysis of this cluster in Xcc and the one in Xac306 shows the following differences. Xac306 has an additional set of genes between *nixI* and *nixJ*: a gene encoding a TonB-dependent receptor (TBDR), a second copy of the beta-galactosidase gene (*bga*), a cation transporter gene (*yhfM*), a ribokinase gene (*rbk*), and a 6-phospho-beta-glucosidase gene (*celF*). The downstream region in Xcc has transposable elements, genes that encode restriction and modification systems, and the following three T3SS effector genes: *xopD*, which is involved with suppression of plant defense^34^ and is found only in Xcc; *xopK*, which has an unknown function^35^; and *xopL*, which is a leucine rich repeat protein^36^. Other proteins putatively involved in catalysis of glycosidic bonds, such as neuraminidase and asparaginase, are found in the genomic neighborhood of the *nix* cluster (Fig. 5a).

The fourth protein family related to N-glycan degradation is another copy of alpha-L-fucosidase (FucA1). This family also appears to be associated with a cluster involved in the degradation of this polymer since downstream genes include another copy of beta-galactosidase (*bga*), an arabinogalactan endo-1,4-beta-galactosidase (*galA*), and another gene encoding TBDR, all of which are conserved in *Xanthomonas* (Fig. 5d). The presence of transposable elements was also found in Xcc, not only within the cluster, but also in the upstream flanking region.

According to the CAZy (carbohydrate-active enzymes) database^37^ there are only two copies of alpha-L-fucosidase (GH4 group) in *Xanthomonas* and *Xylella* genomes, and both copies are enriched in phytopathogens. This may be an adaptation to the presence of fucose residues in plant N-glycans, which is not observed in other organisms.

A database search for each of the four proteins involved in N-glycan degradation showed a mixture of bacteria from different phyla (Fig. 5e), which is a distinct distribution pattern when compared to other protein families analyzed in this study. Most of the microorganisms retrieved in the search are associated with plants and soil^38^.

### Family 7: *VirK*, a new target for study of plant-bacteria interactions

**Figure 6:**
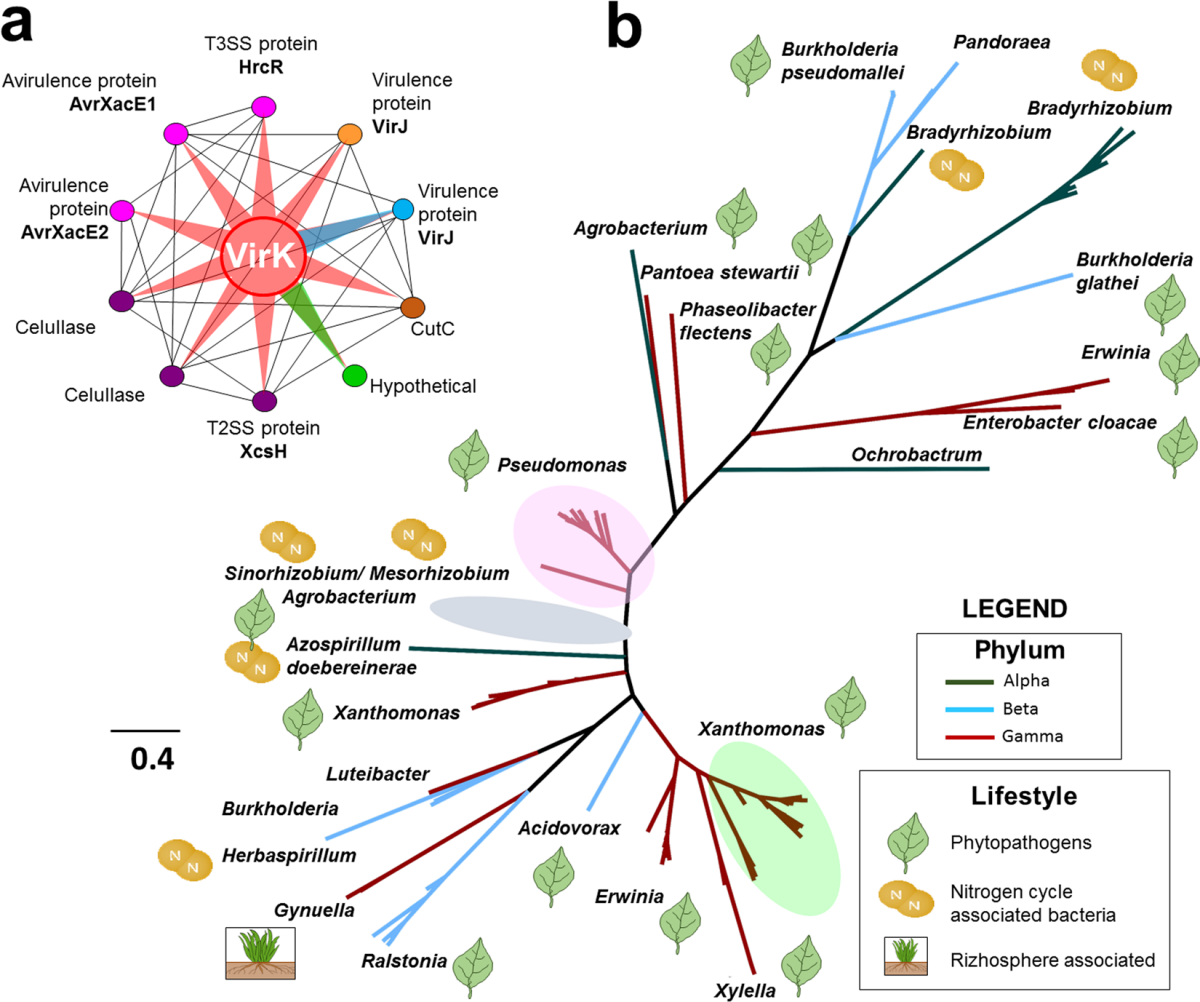
VirK interaction and phylogenetic analysis. (**a**) VirK interaction model, adapted from STITCH 4.0^77^. Circles denote nodes corresponding to each protein that interact with VirK (central). Proteins associated with T3SS are shown in pink and proteins associated with T2SS are highlighted in purple. VirJ corresponds to a protein annotated as associated with T4SS^92^ and phytopathogen genomes have two copies of VirJ. The copies present in all 69 genomes are shown in orange while blue highlights the copy specific to most phytopathogens. VirK associates with a copper resistance protein shown in brown (CutC) and with a hypothetical protein (shown in green) whose gene always appears concatenated downstream from *virK*. (**b**) VirK phylogeny. Check for details at Fig. S16 – Supporting Information.

Among the seven protein families highlighted in this study, the most intriguing is VirK. Until now, no study has determined its actual function. To better understand VirK function, several *in silico* investigations were performed. The encoded protein contains a signal peptide with approximately 22 residues, and secondary structure analysis of the mature protein suggested that VirK consists of 10 α-helices (Fig. S8 – Supporting Information). *In silico* analysis of the interaction between VirK and other proteins (Fig. 6a) predicts that VirK interacts with several other proteins, as follows: three T3SS-associated proteins, including HrcR, a protein required for formation of the inner membrane ring of the T3SS apparatus^39^, and the secreted effectors AvrXacE1 (XopE1) and AvrXacE2 (XopE3); XcsH, a T2SS structural protein, and two cellulases; and VirJ, a protein associated with the T4SS apparatus^40^. Curiously, all of the phytopathogen genomes contained two copies of VirJ, and VirK was associated with both copies. One of the copies is specific to the phytopathogen genomes analyzed here, with the exception of the *Xylella* strains Ann-1 and MUL0034 (which lack this copy). The computational predictions also suggest that VirK interacts with a copper resistant protein (CutC), with a hypothetical protein whose gene, in some genomes, appears immediately downstream of *virK*, and with *nixI* (XAC3076) and asparaginase (XAC3092), both of which are related to N-glycan catalysis.

We reconstructed a phylogeny for VirK based on the best 250 Blastp hits (Fig. 6b). The phylogeny suggests pervasive HGT in Proteobacteria, and subsequent selection for maintenance of this gene in specific lineages. The broad distribution of VirK among bacteria associated with plants and its putative interaction partners suggest a possible role for VirK in the modulation of plant immune response during the infection process.

## Discussion

In this section, we seek to determine the relevance of the results described in terms of previous work from the literature.

### LipA/LesA

In the *Xanthomonas* genus, lipases/esterases, polygalacturonases, cellulases, pectate lyases, xylanases, and cellobiosidases have been described as fundamental for cell wall degradation^41^. These enzymes have been analyzed in a study using *Xanthomonas oryzae* to probe for association with the xylanase XynB. These proteins were found to be required for virulence, since mutations in *xynB* caused a massive reduction of the virulent phenotype in rice crops^42^. A high-resolution analysis of LipA structure has shown an all-helical ligand binding module as a distinct functional attachment to the canonical hydrolase catalytic domain^28^. Point mutations that disrupted the carbohydrate anchor site or blocked the pocket, even at a considerable distance from the enzyme active site, abrogated LipA function in plants, as exemplified by loss of both virulence and the ability to elicit host defense responses. In addition, LipA, cellulase (ClsA) and a putative cellobiosidase (CbsA) were identified in *X. oryzae* pv. *oryzae* as type II secretory system (T2SS) effectors that induce the rice defense response, which are then repressed by T3SS effectors to ensure successful infection^43^.

Lipases/esterases and polygalacturonases have also been studied In the *Xylella* genus^27,44^. In *Xylella fastidiosa*, three new pathogenicity effectors have recently been described, and one of them (LesA) is a lipase/esterase homolog of LipA. The same study demonstrated that paralogs of genes that encode LipA were found in the *Xylella* genome and that one of these copies is concatenated upstream of *lipA*. LesA has recently been identified in the secretome of *Xylella*, and *lesA* mutants were shown to be not virulent when inoculated into grapevines^27^. Among cell-wall degrading enzymes identified in the *Xylella* secretome, three copies of lipase/esterase were among the most abundant (LesA, LesB and LesC). Finally, the same authors demonstrated that the LipA ortholog is secreted into outer membrane vesicles, and the enzyme was also detected in infected host tissue^27^. The duplicated secretory virulence factors of *Xylella fastidiosa* (three copies of lipase/esterase) exemplify the positive selection pressures exerted on advantageous genes in such pathogens^45^. These increased gene copy numbers in *Xylella* genomes suggest the importance of this enzymatic activity for successful colonization of plants. Literature results show that in *Xanthomonas* and *Xylella* the role of lipase/esterase in virulence varies since it is essential in *Xylella* but dispensable in *Xanthomonas*, despite both being enzymatically active^27^.

Although LipA/LesA has a lipase/esterase function and belongs to a large sub-group of α/β-hydrolases, Xanthomonadaceae LipA represents a new catalysis model with a finely adjusted activity^28^. This finding explains its plant-associated function, characterizing LipA as a notable example of adaptation in phytopathogenic organisms. These authors also suggest that this enzyme is present in a common ancestor of *Xanthomonas* and *Xylella* (phytopathogens) and present in *Burkholderia* species (commensal bacteria associated with plants). Although evolutionarily distant from *Xanthomonas* and *Xylella*, our hypothesis is that *Burkholderia* species would require this molecular speciation because of their association with plants.

### Secreted chorismate mutase (*CM*-*sec*)

CM is a component of the shikimate pathway (Fig. S4 – Supporting Information) and is required for the conversion of chorismate to prephenate, a precursor for phenylalanine (Phe) and tyrosine (Tyr) synthesis^46^. In several organisms, chorismate can also be converted to precursors for tryptophan (Trp) synthesis^47^. In plants, CM acts as a precursor of salicylic acid biosynthesis, an important signaling molecule in the plant immune response^48,49^.

We identified two CM genes, which we named *CM-sec* and *CM-nonsec*. Although both should be able convert chorismate to prephenate, *CM-nonsec* may also be able to convert prephenate to phenylpyruvate, which would be converted to Phe and Tyr^50^. Therefore, *CM-nonsec* likely provides these two amino acids during cell metabolism due to the presence of a Phe-regulatory domain at the C-terminal position that functions to down-regulate this metabolic branch^51,52^.

In previous studies, *X. oryzae* pv. *oryzae CM-sec* knockout mutants were significantly more virulent in rice than the wild type (wt) strain^53^. In addition to differences in lesion size, disease symptoms were also stronger in *CM-sec* knockout mutants. When the *CM-sec* defective strain was complemented with the *CM-sec* functional gene, the virulence was similar to that of the wt^53^. In fact, reduction of virulence associated with *CM-sec* suggests that the protein coded by this gene attenuates the plant’s antibacterial response. Therefore, *CM-sec* may be an important evolutionary adaptation of phytopathogenic species to help ensure survival of bacteria inside the host. A supporting literature result is that *CM-sec* also decreases salicylic acid synthesis in plants^31^. *CM-sec* is probably secreted via T2SS^53^. Moreover, differential expression analysis of secreted proteins from an Xac306 wt and an *hrpB4* mutant showed that *CM-sec* is secreted when both strains are grown in XAM1^54^. Since the absence of a functional T3SS in *hrpB4* mutants did not affect *CM-sec* secretion, is was concluded that *CM-sec* is not secreted through the T3SS. Recently it has been shown that CM is one of the proteins secreted by the T2SS in *Xylella fastidiosa* ^27^, but its precise substrates both in *Xanthomonas* and *Xylella* pathosystems are yet to be determined.

### Proteins involved in N-glycan degradation

Glycans are oligosaccharide chains covalently linked to proteins, and are classified into two types. N-glycans are attached to membrane proteins through a linkage to asparagine in the sequence Asn-Xaa-Ser/Thr, while O-glycans are commonly attached to proteins via Ser/Thr residues^55^. In plants, N-glycans present a conserved structure composed by monomers of xylose, mannose, fucose, N-acetylglucosamine, and galactose^56^. Although N-glycans are usually associated with proteins, free N-glycans (F-NG) have been found in different plant tissues^57,58^. They are either the result of an endoplasmic reticulum (ER) retro-translocation followed by catalysis of endo-β-N-acetylglucosaminidase or N-glycanase, or the result of vesicle trafficking mediated by the Golgi apparatus^59^. Recently, it has been shown that N-glycans attached to ectodomains of plasma membrane pattern recognition receptors (PRR) likely form initial contact sites between plant cells and invading pathogens^60^. The N-glycosylation of membrane proteins, both in the ER and in the Golgi apparatus, has been shown to play a crucial role in plant immunity^61,62^.

*X. campestris* (Xcc) has two gene clusters required for the degradation (*nix*) and internalization of N-glycans and associated carbohydrates monomers (*nag*)^32,33^. The *nix* cluster consists of a gene coding for a TonB-dependent receptor (TBDR) and nine genes (*nixD-L*) with hydrolase function at different glycosidic bonds in the carbohydrate units that compose the N-glycans structure (Fig. 5a,b)^33^. Xac306 and strains of Xcc have a conserved *nag* cluster^32^ (Fig. 5c). Mutations in genes present in both clusters strongly affect the virulence of Xcc in compatible hosts^63^. Interestingly, L-fucosidase was identified in *X. oryzae* secretome only when the bacteria were subjected to growth *in planta*, while glucan 1,4-beta-glucosidase has been detected *in planta* and *in vitro* ^64^. NixE was detected in the culture supernatant of Xcc wt strain, as well as in both T2SS mutant strains, indicating that this protein is secreted in a T2SS-independent manner^33^. Since T2SS is evolutionarily related to the type IV secretory system (T4SS)^65^, NixE may be secreted through T4SS. In contrast, NixF and NixL were not detected in the T2SS mutants, suggesting that these proteins are secreted via the T2SS^33,66^. Based on our results, we hypothesize that Nix proteins could affect the degradation of N-glycans in three different ways: (i) by direct activity on N-glycans associated with plant membrane receptors (EFR and FLS2); (ii) by degradation inside the ER or Golgi apparatus, which prevents formation of mature glycosylated receptors; or (iii) by degradation of free N-glycans. The first two mechanisms may reduce the immune response due to non-recognition of the bacterial EF-Tu and flagellin effectors, and possibly other pathogen-associated molecular patterns (PAMPs).

### VirK

Differential expression analyses have indicated that *virK*/VirK is always induced under infectious conditions or in culture media that mimic survival conditions *in planta* ^67,68^. Another study confirmed the differential expression profile of 32 genes from Xac306 that were putatively associated with virulence in medium containing copper^69^. That work provided additional evidence that the expression profile of *virK* follows that of genes associated with virulence. It is noteworthy that seven out of 10 proteins differentially expressed in both studies were found to interact with VirK, as determined by STITCH 4.0 (Fig. 6).

VirK was also one of the most highly expressed proteins, in infectious conditions or in simulations of such conditions. Expression of 11 proteins (including VirK) secreted by T2SS from Xac306 was higher in a HrpG* mutant with the ability to induce the expression of T3SS-related genes even under nutrient-rich conditions^70^. 109 proteins from the secretome of *X*. *oryzae* grown *in planta* and *in vitro* were identified using 2DE coupled with MALDI-TOF-MS and/or nLC-ESI-MS/MS; VirK and XadA (adhesin, outer membrane protein) were the only proteins associated with virulence detected exclusively *in planta* ^64^. In another study, VirK was one of the 64 identified proteins in vascular fluids from infected rice plants, in conjunction with hydrolytic enzymes involved in chemotaxis, membrane proteins, and proteins classically related to PAMPs such as flagellin and EF-Tu^68^. An analysis of the secretome of Xac306 wt and *hrpB4* (associated with T3SS) mutant cultivated in rich vs. virulence induction medium confirmed that VirK together with cellulases and polygalacturonases is one of 14 proteins detected only in the wt secretome when the bacteria were grown in virulence induction medium^54^. All of these reports support the hypothesis that secretion of VirK is important for pathogen-host interactions, although its precise function remains unknown.

Reports in the literature show that VirK is secreted by the T2SS^70^ or by the T4SS^65^. Additional evidence for secretion through the T2SS is given by the finding that HrpG regulates VirK in *Ralstonia solanacearum* ^71^, as some proteins secreted by the T2SS are regulated by HrpX/G, a two-component system that plays global roles in coordinating different virulence traits of *Xanthomonas axonopodis* pv. *citri* ^72^.

### Co-evolutionary implications

Our analysis suggests that the seven protein families are examples of molecular speciation involved in the survival maintenance of phytopathogens inside plants. The proteins belonging to these seven protein families are putatively secreted by the T2SS and can be classified as effector proteins, directly or indirectly correlated to the induction or evasion of the plant immune system. We hypothesize that the evolution of the corresponding genes is a result of the ‘arms race’ between plants and these phytopathogens^73^. We further speculate that our results are not restricted to the phytopathogens in the *Xanthomonas* and *Xylella* genera, but apply as well to all plant-associated bacteria that have true orthologs of these proteins.

**Figure 7:**
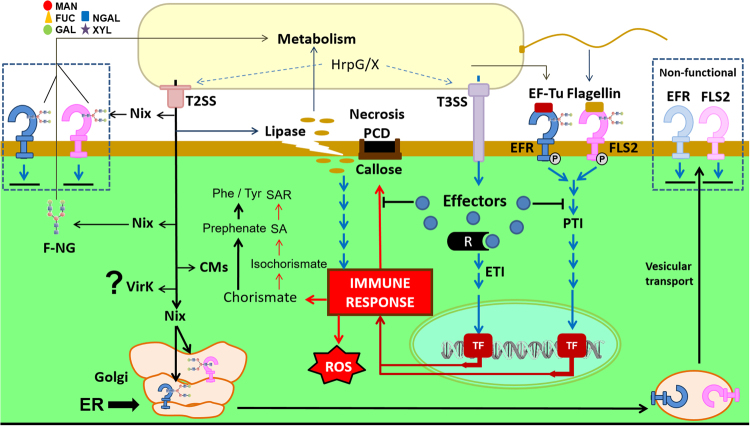
Overview of the seven plant-associated specific proteins and its correlation with induction and evasion of the plant immune system. The arrows in black highlight possible activities of these seven enzymes putatively secreted by T2SS. The Nix nomenclature summarizes the activity of enzymes that are part of the whole *nix* cluster, with particular emphasis on NixE, NixF and NixL. The blue arrows highlight induction of the immune response mediated by DAMP/PTI (mediated by lipase action) and PAMP (mediated by EF-Tu and flagellin), and evasion of immune response induced by ETI (mediated by T3SS effectors). The immune system activation responses (in red) trigger the production of reactive oxygen species (ROS), induction of systemic acquired resistance (SAR) and generation of calloses, necrosis, and programmed cell death (PCD). It is possible that Nix operates in two contexts: in free N-glycans (F-NG) or in N-glycans associated with membrane receptors (EFR and FLS2). The action of Nix proteins could degrade N-glycans that should be linked to these receptors at Golgi and directly degrade N-glycans from receptors on the plant surface. Both byproducts of N-glycans or cell wall degradation may still be used as a carbon source for bacterial metabolism. DAMP: Damage-Associated Molecular Patterns. PTI: PAMP-Triggered Immunity. ETI: Effector-Triggered Immunity.

According to the model proposed in Fig. 7, inside plant tissues *CM-sec* would be related to displacement of plant metabolism for synthesis of aromatic amino acids. As a result, there is a reduction in the production of salicylic acid, a plant hormone that induces the plant defenses against a variety of biotic and abiotic stress by modulating biochemical, morphological, and physiological mechanisms. Likewise, other studies have shown that the lipase/esterase, LipA, is associated with degradation of the plant cell wall. Although the action of this enzyme disrupts the plant’s first defensive barrier so that invading phytopathogens are able to colonize plant tissues, byproducts of this degradation are potential activators of innate immune responses in plants. Moreover, although N-glycans are structures that may be present as free polymers in plant cells, they are essential structures of receptors on the plant cell membrane. Thus, insertion of this polymer into these proteins occurs in the Golgi apparatus, and deficiency in this process culminates in receptors that cannot transduce molecular signals. In fact, loss of the transduction capacity associated with receptors such as EFR and FLS2, which recognize EF-Tu and flagellin proteins, respectively, would reduce the plant immune response. In addition, degradation of N-glycans linked to these receptors on the plant surface by Nix enzymes decreases the immune response while simultaneously facilitating the penetration of bacteria into the plant tissue. Finally, although VirK has no known function to date, our results provide additional evidence that it is a secreted protein, and it is specific to bacteria associated with plants. In addition, VirK is associated with proteins involved in the T2SS, leading us to hypothesize that VirK acts as a new effector associated with reduction of the plant defense response.

## Conclusion

Our study identified seven protein families whose characteristics make them potential molecular targets in dealing with a variety of plant diseases. These results demonstrate the importance and potential of comparative genomics studies to elucidate intriguing biological processes, and the fact that genomic approaches are transforming our understanding of co-evolutionary interactions between bacteria and plants. Furthermore, given the inferred importance of selection in maintaining, or in some cases, duplicating, the genes mentioned in this study, for survival of the respective phytopathogenic lineages, we speculate that some plant-infecting Xanthomonadaceae are transitioning to a mutualistic equilibrium with their plant hosts. This equilibrium may eventually succeed when bacterial virulence and plant development are both successfully adjusted, a possible outcome in the evolution of parasitic bacteria. Therefore, the present study may broaden our knowledge of plant-parasite interactions within the Xanthomonadaceae family, while also contributing to knowledge of host-parasite evolution in general. A complete understanding of the molecular basis of plant disease resistance will enable the application of these discoveries to generate plants that contain tuned disease-resistance pathways that are durable and recognize a wide spectrum of pathogens.

## Methods

### Genome selection

We included in our study 69 completely sequenced genomes available at NCBI database as of October 2015 belonging to the genera *Pseudoxanthomonas* (3), *Stenotrophomonas* (8), *Xanthomonas* (51), and *Xylella* (7) – Fig. S1 and Table S1 (Supporting Information).

### Prediction of protein families

Analysis of the presence or absence of each protein family among the 69 genomes allowed us to predict unique, flexible and core protein families that are shared by a sub-set of bacterial strains using Orthologsorter^74^ available at http://jau.facom.ufms.br/xanthomonadaceae2/orthologsorter/. This tool, whose interface was developed specifically for this purpose enables the user to identify sets of specific protein families from each genome or shared among selected genomes according to a specific interest. Only seven protein families were found from the selection of proteins shared exclusively among the 58 phytopathogens analyzed (see results and discussion). These seven families are comprised by the 420 proteins considered in this work and were found by asking Orthologsorter which families contain at least one protein from the phytopathogen genomes and also do not contain any genes of the other 11 non-pathogenic ones. Protein families have been found by OrthoMCL^75^, that is a very well-known genome-scale software for grouping orthologous protein sequences, based on Blast reciprocal matches and MCL clustering methods.

In order to confirm whether these seven protein families are specific to the phytopathogens analyzed, avoiding problems related to annotation heterogeneity, an amino acid versus nucleotide tBLASTN alignment was performed using the protein sequences of Xac306 genes as queries and a BLAST database composed of the genome sequences from the 69 fully-sequenced genomes as subject with maximum e-value set to 10^−10^. This method generated no new hits compared to the information initially gathered from the annotation of the 68 genomes (excluding Xac306) (Table S5 – Supporting Information).

### Phylogenomic profile

The phylogenomic profile was predicted based on the core protein families. First OrthoMCL found 10 432 families, of which only 846 had exactly one protein from each genome. These families were aligned and concatenated (295 895 columns) using MUSCLE^76^. After the removal of non-informative columns (columns that may not be conserved, including columns with too many gaps, or that may be saturated by multiple substitutions) using GBlocks^77^, only 1 362 columns remained. The tree was then generated from the 1 362 columns using RAxML^78^ with the PROTCAT model, rapid bootstrapping with 100 replicates, and maximum likelihood search.

### Phylogenetic analysis of the seven proteins families

For phylogenetic analysis of each one of the seven protein families specific to the phytopathogens analyzed, we used protein sequences from *Xanthomonas axonopodis* pv. *citri* strain 306 pathotype A – Xac306 (NCBI accession NC_003919.1) as the query for a Blastp search^79^, with the maximum number of hits set to 250. Protein multiple alignment was performed using the G-INS-i algorithm in Mafft 7.25^80^ with 16 iterative refinement steps. TrimAl v1.4 was used to remove columns with an excess of gaps^81^. IQTree v1.3.11^82^ was used to predict the best phylogenetic model out of 468 tested and to estimate the best maximum likelihood (ML) tree with branch support obtained by 1 000 bootstrap pseudo replicates. Tree visualization, rooting, and branch coloring were all performed in FigTree v1.4.2 (http://tree.bio.ed.ac.uk/software/figtree/). Due to unknown relationships among higher-order bacteria clades that appeared in gene trees (e.g., Proteobacteria *vs*. Actinobacteria), we used midpoint rooting to infer polarity of evolution. Branch coloring among higher-order bacteria lineages was kept the same across the studied genes. Figure S2 (Supporting Information) shows the pipeline of computational analyses performed in this work. Additional computational methods are described in Supplementary Methods.

### Strains and growth conditions

The wild type *Xanthomonas axonopodis* pv. *citri* strain 306 (Xac306_wt_) and mutants were grown in Nutrient Broth (NB) medium with aeration (220 rpm) at 28 °C. Plate cultures were prepared in the same medium with the addition of 1.5% agar. NB medium was supplemented with kanamycin (10 μg/mL) for selective growth of the *lesA* mutants. Xac306_wt_ strain used in this study was kindly provided by Dr. Chuck Farah, University of São Paulo, Brazil. For heterologous expression of LesA from Xac306_wt_, *Escherichia coli* strain DH5α cells were grown in Luria-Bertani (LB) medium supplemented with kanamycin (50 μg/mL) at 37 °C and 120 rpm.

### Heterologous expression of *LipA*

Heterologous expression of LipA was performed by cloning XAC0501 in pJexpress 401 vector (DNA2.0, USA). The insertion of the cloned gene was confirmed by PCR using primers flanking *lipA*, followed by transformation of *E. coli* strain DH5α competent cells. Cells were grown in LB broth supplemented with kanamycin (50 μg/mL) at 37 °C and 120 rpm until OD_600_ 0.8, when 1 mM IPTG was added and cells cultured for additional 3 h at 30 °C. Total protein was extracted from *E. coli* cells using a lysis buffer (1 M Tris-HCl pH 7.5; 5 M NaCl; 10 mg/mL lysozyme; 1% glycerol; 100 mM benzamidine; 0.5 M EDTA; 100 mM PMSF). The protein concentration was determined according to Bradford’s method using BSA as the standard.

### Western blot analysis

To detect LipA, 10 μg of total protein extracted from *E. coli* cells was evaluated by SDS-PAGE and transferred by electroblotting to nitrocellulose membrane (BioRad, USA). HRP-conjugated anti-Flag antibody was diluted in PBS-M 1% (PBS plus 1% skim powdered milk 1:1000), followed by incubation of 3 h. Blocking and washing steps were performed with PBS-M 5% (PBS plus 5% skim powdered milk) and PBS-T 0.1% (PBS plus 0.1% Tween 20), respectively. Developments were carried out using ECL Plus western blotting detection reagents (GE Life Sciences, USA).

### Lipase and esterase activity assays

Tributyrin (C4) (Sigma-Aldrich, USA) was used as substrate for LipA activity in a plate assay. To test the lipase activity of the total protein extract from *E. coli*, the triglyceride substrate (1%; v/v) was prepared in a buffer containing 100 mM Tris-HCl (pH 8.0), 25 mM CaCl2, 2% agarose and solidified in Petri dishes. Total protein extracts (1 µg/μL) were added to the wells and assayed for a zone of clearance for 24 to 48 h at room temperature (23 °C). For the *E. coli* and Xac306 *lipA* mutant lipase activity assay, tributyrin (1%; v/v) was emulsified in LB and NB agar medium, respectively. The plates were incubated at 37 and 28 °C for about five days, enough time for the emergence of tributyrin degradation halos surrounding the inoculation holes. Esterase activity was also determined using 4-methylumbelliferyl butyrate (4-MUB) substrate as previously described by Vaneechoutte, *et al*.^83^.

### Hypersensitive response assay in Nicotiana tabacum leaves

Total proteins from empty vector and LipA-expressing *E. coli* were used for syringe infiltration in greenhouse-grown *Nicotiana tabacum* leaves (plants were approximately two months of age and 30 cm height). The infiltration occurred with the aid of a needleless syringe in spots previously pierced with a needle. Ninety μg of protein (0.3 μg/μL) was infiltrated into each leaf spot. HR-like lesions in the infiltrated area were photographed 24 h after inoculation.

### Isolation of lipA mutants

The mutagenesis cassette was chemically synthesized (GenScript, USA) after insertion of a kanamycin resistance gene in the central portion of the target gene XAC0501 (within Ser-200, the first amino acid of the active triad, maintaining the 5′ and 3′ unchanged to allow homologous recombination). For confirmation of the mutated locus, oligonucleotides Xac0501MutF: TGACCACTCACGCTTCTTCC and Xac0501MutR: CAACCATCGTACCCACTCTATC were used for PCR amplification.

### Citrus leaf infection

Mutant and wild type strains of Xac306 were grown in liquid medium NB, centrifuged (5000 x g, 10 minutes, 4 °C) and washed twice with 25 mM CaCl_2_. The optical density was adjusted to OD_600_ of 0.5. This suspension was then infiltrated into the lower surface of leaves of orange seedlings (*Citrus sinensis*, Valencia), approximately 50 cm in height. The infiltration occurred with the aid of a syringe in spots previously pierced with a needle to drench the leaf blade. The treatments of this experiment were done in triplicate. Lesions in the infiltrated area were photographed twenty-five days after inoculation.

### Data Availability

All data used in this work is publicly available at NCBI^6^.

## Electronic supplementary material

Supplementary information
