# Supplementary material for: Identification and analysis of seven effector protein families with different adaptive and evolutionary histories in plant-associated members of the Xanthomonadaceae
Source: Sci Rep. 2017 Nov 23;7:16133. doi: 10.1038/s41598-017-16325-1 (PMC5700972; doi:10.1038/s41598-017-16325-1)
Supplement: Supplementary file 1 — Supplementary information [file 41598_2017_16325_MOESM1_ESM.pdf]

Supplementary Information

Identification and analysis of seven effector protein families with different adaptive and evolutionary histories in plant-associated members of the Xanthomonadaceae

Renata de A. B. Assis, Lorraine Cristina Polloni, José S. L. Patané, Shalabh Thakur, Érica B. Felestrino, Julio Diaz-Caballero, Luciano Antonio Digiampietri, Luiz Ricardo Goulart, Nalvo F. Almeida, Rafael Nascimento, Abhaya M. Dandekar, Paulo A. Zaini, João C. Setubal, David S. Guttman, and Leandro Marcio Moreira*

Department of Biological Science, Institute of Exact and Biological Science, Federal University of Ouro Preto, Ouro Preto, MG, Brazil.

Corresponding author`s e-mail lmmorei@gmail.com; Tel +55 (31) 3559-1698; Fax +55 (31) 3559-1672

CONTENTS

Supplementary Methods - Additional computational analyses performed in this work................ 4

Supplementary Tables.. 5

Table S1. Genome features.. 5

Table S2. Members of the seven protein families enriched in Xanthomonadaceae phytopathogens.. 12

Table S3. Lipases found in *Xanthomonas* and the different functional parameters of each. 24

Table S4. LipA canonical residues analysis in different genomes...................................... 28

Table S5. Validation of the phytopathogen core genome using tblastn. 29

Supplementary Figures 36

Fig. S1. Comparative genomics of the 69 selected strains. (a) General information about each strain classified into phytopathogens (green) and non-phytopathogens (brown). (b) Number of chromosome and plasmid proteins of respective strain. Numbers above the bars indicate number of plasmids in each strain. (c) Number of singletons per genome classified into three annotation categories.. 36

Fig. S2. Flowchart of analytical steps involved in this work... 37

Fig. S3. Domain composition analysis of the seven proteins families using Xac306 as reference. 38

Fig. S4. Shikimate pathway highlighting the possible reactions catalyzed by *CM-sec* and *CM-nonsec* in the metabolism of plants and phytopathogens... 39

Fig. S5. Signal peptide prediction of CM-sec and CM-nonsec.. 40

Fig. S6. Structural analysis of chorismate mutase. 41

Fig. S7. Structural parameters analysis that denotes quality of *CM-sec* protein topology. .. 42

Fig. S8. VirK structural analysis... 43

Fig. S9. Maximum likelihood phylogenetic tree of LipA.. 44

Fig. S10. Maximum likelihood phylogenetic tree of *CM-sec*.. 45

Fig. S11. Maximum likelihood phylogenetic tree of CM-nonsec.. 46

Fig. S12. Maximum likelihood phylogenetic tree of NixE.. 47

Fig. S13. Maximum likelihood phylogenetic tree of NixF. 48

Fig. S14. Maximum likelihood phylogenetic tree of NixL. 49

Fig. S15. Maximum likelihood phylogenetic tree of FucA1. 50

Fig. S16. Maximum likelihood phylogenetic tree of VirK. 51

Supplementary Methods - Additional computational analyses performed in this work

Prediction of signal peptide, cellular localization, and domain composition

To predict the presence, location and cleavage sites of signal peptides in all phytopathogen-specific proteins, we used SignalP [79], Phobius [80], and TatP [81] tools. In addition, we used PSORT [82] to verify cellular localization, and Kyoto Encyclopedia of Genes and Genomes – KEGG [83] and SMART [84] to determine the number of domains for each protein.

Prediction of genes in horizontal gene transfer (HGT) regions and protein-protein/protein-chemical interactions

To predict the presence of the genes that code to the seven proteins families in HGT regions we used IslandViewer [85]. To predict the possible protein-protein or protein-chemical interactions we used STITCH 4.0 [86].

Prediction of transcriptional units of N-glycan degradation genes

For prediction of transcriptional units, we used the program ProOpDB [87].

Reannotation with assigned function

The reannotation of hypothetical genes was made based on clusters of orthologous groups – COG [88].

Secondary and three-dimensional structure prediction

Secondary structure analysis was determined using the Jpred4 tool [89]. Topology prediction analyses were obtained from Swiss-model [90] and Phyre2 [91]. The structural parameters were obtained from Phyre2 [91].

Supplementary Tables

Table S1. Genome features.

Strain	Abbreviation	Niche	Genome Features							Hosts	References
			Type	Ref_Sec	INSDC	Size (Mb)	GC%	Protein	Genes		
X.axonopodis pv. *citri* strain 306	Xac306	Mesophyllic phytopathogen	Chr	NC_003919.1	AE008923.1	5.18	64.8	4,232	4,406	Citrus	Nature. 2002 May 23;417(6887):459-63. PMID: 12024217 Zhang et al 2015
			pXac33	NC_003921.3	AE008924.1	0.0337	61.9	34	36		
			pXac64	NC_003922.1	AE008925.1	0.06492	61.4	61	65		
X.axonopodis strain 29-1	XacA29	Mesophyllic phytopathogen	Chr	NC_020800.1	CP004399.1	5.15	64.8	4,136	4,393	Citrus	Direct Submission / Unpublished Zhang et al 2015
			pXAC64	NC_020797.1	CP004400.1	0.064118	61.6	59	62		
			pXAC47	NC_020798.1	CP004401.1	0.047151	61.8	50	53		
			pXAC33	NC_020801.1	CP004402.1	0.031801	61.9	33	34		
X.axonopodis citri subsp. *citri* strain Aw12879	XacAw12879	Mesophyllic phytopathogen	Chr	NC_020815.1	CP003778.1	5.32	64.7	4,343	4,559	Citrus	Genome Announc. 2013 May 16;1(3). PMID: 23682143 Zhang et al 2015
			pXcaw19	NC_020816.1	CP003779.1	0.018869	63.1	16	16		
			pXcaw58	NC_020817.1	CP003780.1	0.058317	61.9	55	62		
X.campestris pv. *vesicatoria* strain 85-10	Xcv8510	Mesophyllic phytopathogen	Chr	NC_007508.1	AM039952.1	5.18	64.7	4,211	4,442	Pepper and tomato	J Bacteriol. 2005 Nov;187(21):7254-66. PMID: 16237009 Thieme et al 2005
			pXCV2	NC_007504.1	AM039948.1	0.001852	56.6	2	2		
			pXCV19	NC_007505.1	AM039949.1	0.019146	59.8	22	23		
			pXCV38	NC_007506.1	AM039950.1	0.038116	60.7	48	48		
			pXCV183	NC_007507.1	AM039951.1	0.182572	60.5	175	178		
X.axonopodis pv. citrumelo strain F1	XalfaF1	Mesophyllic phytopathogen	Chr	NC_016010.1	CP002914.1	4.97	64.9	4,020	4,188	Brassica	J Bacteriol. 2011 Nov;193(22):6342-57. Epub 2011 Sep 9. PMID: 21908674 Zhang et al 2015
X.oryzae pv. *oryzicola* strain BLS256	XooBLS256	Mesophyllic phytopathogen	Chr	NC_017267.2	CP003057.2	4.83	64.1	3,755	4,161	Oryza	J Bacteriol. 2011 Oct;193(19):5450-64. Epub 2011 Jul 22. PMID: 21784931 Bogdanove et al 2011
X.oryzae pv. *oryzae* strain PXO99A	XoryPXO99A	Vascular phytopathogen	Chr	NC_010717.2	CP000967.2	5.24	63.6	4,375	4,798	Oryza	BMC Genomics. 2008 May 1;9:204. PMID: 18452608 Qian et al 2005
X.oryzae pv. *oryzae strain* KACC10331	XoryKACC10331	Vascular phytopathogen	Chr	NC_006834.1	AE013598.1	4.94	63.7	4,055	4,568	Oryza	Nucleic Acids Res. 2005 Jan 26;33(2):577-86. PMID: 15673718 Qian et al 2005

X.oryzae pv. oryzae strain MAFF 311018	XoryMAFF311018	Vascular phytopathogen	Chr	NC_007705.1	AP008229.1	4.94	63.7	4,183	4,575	Oryza	Direct Submission / Unpublished Qian et al 2005
X.campestris pv. *raphani* strain 756C	Xcr756C	Mesophyllic phytopathogen	Chr	NC_017271.1	CP002789.1	4.94	65.3	4,007	4,140	Brassica and Solanaceae	J Bacteriol. 2011 Oct;193(19):5450-64. Epub 2011 Jul 22. PMID: 21784931 Bogdanove et al 2011
X.campestris pv. campestris strain 8004	Xcam8004	Vascular phytopathogen	Chr	NC_007086.1	CP000050.1	5.15	65	4,248	4,381	Brassica	Genome Res. 2005 Jun;15(6):757-67. Epub 2005 May 17. PMID: 15899963 Qian et al 2005
X.campestris pv. campestris strain ATCC 33913	XcamATCC33913	Vascular phytopathogen	Chr	NC_003902.1	AE008922.1	5.08	65.1	4,179	4,240	Brassica	Nature. 2002 May 23;417(6887):459-63. PMID: 12024217 Qian et al 2005
X.fastidiosa strain 9a5c	Xyf9a5c	Xylem-limited phytopathogen	Chr	NC_002488.3	AE003849.1	2.68	52.7	2,247	2,420	Citrus	Nature. 2000 Jul 13;406(6792):151-9. PMID: 10910347 Simpson et al 2000
			pXF1.3	NC_002489.3	AE003850.3	0.001286	55.6	1	1		
			pXF51	NC_002490.1	AE003851.1	0.051158	49.6	62	66		
X.fastidiosa strain M12	XyfM12	Xylem-limited phytopathogen	Chr	NC_010513.1	CP000941.1	2.48	51.9	2,054	2,187	Almond	J Bacteriol. 2010 Sep;192(17):4534. Epub 2010 Jul 2. PMID: 20601474 Chen et al 2010
X.fastidiosa strain GB514	XyfGB514	Xylem-limited phytopathogen	Chr	NC_017562.1	CP002165.1	2.49	51.8	1,957	2,172	Grape	Direct Submission / Unpublished Schreiber et al 2010
			unnamed	NC_017561.1	CP002166.1	0.02618	49.3	35	37		
X.fastidiosa strain M23	XyfM23	Xylem-limited phytopathogen	Chr	NC_010577.1	CP001011.1	2.54	51.8	2,152	2,242	Almond	J Bacteriol. 2010 Sep;192(17):4534. Epub 2010 Jul 2. PMID: 20601474 Chen et al 2010
			pXFAS01	NC_010579.1	CP001012.1	0.038297	49.2	41	42		
X.fastidiosa strain Temecula1	XyfTemecula1	Xylem-limited phytopathogen	Chr	NC_004556.1	AE009442.1	2.52	51.8	2,156	2,247	Grapevine	J Bacteriol. 2003 Feb;185(3):1018-26. PMID: 12533478 Van Sluys et al 2003
			pXFPD1.3	NC_004554.1	AE009443.1	0.001346	53.8	1	1		
X.albilineans strain GPE PC73	XalbGPEPC73	Xylem-limited phytopathogen	Chr	NC_013722.1	FP565176.1	3.77	63	2,946	3,104	Sugar cane	BMC Genomics. 2009 Dec 17;10:616. PMID: 20017926 Pieretti et al 2009
			plasmIII	NC_017555.1	FP340277.1	0.027212	57.3	31	31		
			plasmII	NC_017556.1	FP340278.1	0.031555	60	34	35		
			plasmI	NC_017557.1	FP340279.1	0.024837	58.4	27	28		
P.suwonensis strain 11-1	Psuw111	Compost feedstock-adapted	Chr	NC_014924.1	CP002446.1	3.42	70.2	3,023	3,109	Compost feedstock	Direct Submission / Unpublished Choi et al 2013
P.spadix strain BD-a59	PspaBDa59	Gasoline-contaminated sediment	Chr	NC_016147.2	CP003093.2	3.45	67.7	2,994	3,104	Soil	J Bacteriol. 2012 Jan;194(2):544. PMID: 22207748
S.maltophilia strain R551-3	SmalR5513	Endophytic	Chr	NC_011071.1	CP001111.1	4.57	66.3	4,011	4,112	Endophyte	Direct Submission / Unpublished Alavi et al 2014

S.maltophilia strain K279a	SmalK279a	Opportunistic pathogen	Chr	NC_010943.1	AM743169.1	4.85	66.3	4,328	4,458	Human pathogen	Genome Biol. 2008 Apr 17;9(4):R74. PMID: 18419807
S.maltophilia strain JV3	SmalJV3	Rhizosphere-colonizing	Chr	NC_015947.1	CP002986.1	4.54	66.9	4,022	4,128	Rhizosphere	Direct Submission / Unpublished https://www.ncbi.nlm.nih.gov/nuccore/NC_015947.1
S.maltophilia strain D457	SmalD457	Opportunistic pathogen	Chr	NC_017671.1	HE798556.1	4.77	66.8	4,220	4,381	Human pathogen	J Bacteriol. 2012 Jul;194(13):3563-4. PMID: 22689246
P.suwonensis strain J1	PsuwJ1	Leafy wood soil	Chr	NZ_CP011144.1	CP011144.1	3.89	70.2	3,136	3,222	Soil	Genome Announc. 2015 Jun 11;3(3). PMID: 26067962
S.acidaminiphila strain ZAC14D2_NAIMI4_2	StenoacidaZAC14D2	Superficial sediment of polluted river	Chr	NZ_CP012900.1	CP012900.1	4.14	68.5	3,513	3,656	River sediment	Genome Announc. 2015 Dec 10;3(6). PMID: 26659678
S.maltophilia strain ISMM2	StenomaltoISMMS2	Opportunistic pathogen	Chr	NZ_CP011305.1	CP011305.1	4.51	66.4	3,964	4,076	Human pathogen	Antimicrob Agents Chemother. 2015 Nov;59(11):7117-20. Epub 2015 Aug 31. PMID: 26324280
S.maltophilia strain ISMMS2R	StenomaltoISMMS2R	Opportunistic pathogen	Chr	NZ_CP011306.1	CP011306.1	4.51	66.4	3,964	4,076	Human pathogen	Antimicrob Agents Chemother. 2015 Nov;59(11):7117-20. Epub 2015 Aug 31. PMID: 26324280
S.maltophilia strain ISMMS3	StenomaltoISMMS3	Opportunistic pathogen	Chr	NZ_CP011010.1	CP011010.1	4.8	66.7	4,217	4,335	Human pathogen	Antimicrob Agents Chemother. 2015 Nov;59(11):7117-20. Epub 2015 Aug 31. PMID: 26324280
X.axonopodis pv. *citri* strain 5208	Xac5208	Mesophyllic phytopathogen	Chr	NZ_CP009028.1	CP009028.1	5.18	64.8	4,233	4,407	Citrus	ISME J. 2015 Oct;9(10):2128-38. Epub 2015 Feb 17. PMID: 25689023 Zhang et al 2015
			pXAC33	NZ_CP009026.1	CP009026.1	0.028753	61.7	29	31		
			pXAC64	NZ_CP009027.1	CP009027.1	0.06492	61.4	61	65		
X.citri subsp. *citri* strain A306	XacA306	Mesophyllic phytopathogen	Chr	NZ_CP006857.1	CP006857.1	5.18	64.8	4,228	4,405	Citrus	Direct Submission / Unpublished Zhang et al 2015
			pXAC33	NZ_CP006855.1	CP006855.1	0.033703	61.9	34	36		
			pXAC64	NZ_CP006856.1	CP006856.1	0.06492	61.4	61	65		
X.axonopodis pv. *citri* strain AW13	XacAW13	Mesophyllic phytopathogen	Chr	NZ_CP009031.1	CP009031.1	5.32	64.7	4,354	4,558	Citrus	ISME J. 2015 Oct;9(10):2128-38. Epub 2015 Feb 17. PMID: 25689023 Zhang et al 2015
			pXCAW19	NZ_CP009029.1	CP009029.1	0.018869	63.1	16	16		
			pXCAW58	NZ_CP009030.1	CP009030.1	0.058317	61.9	56	63		
X.axonopodis pv. *citri* strain AW14	XacAW14	Mesophyllic phytopathogen	Chr	NZ_CP009034.1	CP009034.1	5.32	64.7	4,350	4,558	Citrus	ISME J. 2015 Oct;9(10):2128-38. Epub 2015 Feb 17. PMID: 25689023 Zhang et al 2015
			pXCAW19	NZ_CP009032.1	CP009032.1	0.018869	63.1	16	16		
			pXCAW58	NZ_CP009033.1	CP009033.1	0.058317	61.9	56	63		

X.axonopodis pv. *citri* strain AW15	XacAW15	Mesophyllic phytopathogen	Chr	NZ_CP009037.1	CP009037.1	5.32	64.7	4,355	4,559	Citrus	ISME J. 2015 Oct;9(10):2128-38. Epub 2015 Feb 17. PMID: 25689023 Zhang et al 2015
			pXCAW19	NZ_CP009035.1	CP009035.1	0.018869	63.1	16	16		
			pXCAW58	NZ_CP009036.1	CP009036.1	0.058317	61.9	56	63		
X.axonopodis pv. *citri* strain AW16	XacAW16	Mesophyllic phytopathogen	Chr	NZ_CP009040.1	CP009040.1	5.32	64.7	4,356	4,561	Citrus	ISME J. 2015 Oct;9(10):2128-38. Epub 2015 Feb 17. PMID: 25689023 Zhang et al 2015
			pXCAW19	NZ_CP009038.1	CP009038.1	0.018869	63.1	16	16		
			pXCAW58	NZ_CP009039.1	CP009039.1	0.058317	61.9	56	63		
X.axonopodis pv. *citri* strain BL18	XacBL18	Mesophyllic phytopathogen	Chr	NZ_CP009025.1	CP009025.1	5.18	64.8	4,233	4,408	Citrus	ISME J. 2015 Oct;9(10):2128-38. Epub 2015 Feb 17. PMID: 25689023 Zhang et al 2015
			pXAC33	NZ_CP009023.1	CP009023.1	0.026933	61.6	26	28		
			pXAC64	NZ_CP009024.1	CP009024.1	0.06492	61.4	61	65		
X.axonopodis pv. *citri* strain FB19	XacFB19	Mesophyllic phytopathogen	Chr	NZ_CP009022.1	CP009022.1	5.18	64.8	4,233	4,407	Citrus	ISME J. 2015 Oct;9(10):2128-38. Epub 2015 Feb 17. PMID: 25689023 Zhang et al 2015
			pXAC33	NZ_CP009020.1	CP009020.1	0.026933	61.6	26	28		
			pXAC64	NZ_CP009021.1	CP009021.1	0.06492	61.4	61	65		
X.axonopodis pv. *citri* strain GD2	XacGD2	Mesophyllic phytopathogen	Chr	NZ_CP009019.1	CP009019.1	5.13	64.8	4,206	4,376	Citrus	ISME J. 2015 Oct;9(10):2128-38. Epub 2015 Feb 17. PMID: 25689023 Zhang et al 2015
			pXAC33	NZ_CP009017.1	CP009017.1	0.033703	61.9	33	35		
			pXAC64	NZ_CP009018.1	CP009018.1	0.06492	61.4	60	64		
X.axonopodis pv. *citri* strain GD3	XacGD3	Mesophyllic phytopathogen	Chr	NZ_CP009016.1	CP009016.1	5.13	64.8	4,202	4,373	Citrus	ISME J. 2015 Oct;9(10):2128-38. Epub 2015 Feb 17. PMID: 25689023 Zhang et al 2015
			pXAC33	NZ_CP009014.1	CP009014.1	0.033703	61.9	33	35		
			pXAC64	NZ_CP009015.1	CP009015.1	0.064919	61.4	59	64		
X.axonopodis pv. *citri* strain JX4	XacJX4	Mesophyllic phytopathogen	Chr	NZ_CP009013.1	CP009013.1	5.12	64.8	4,201	4,373	Citrus	ISME J. 2015 Oct;9(10):2128-38. Epub 2015 Feb 17. PMID: 25689023 Zhang et al 2015
			pXAC33	NZ_CP009011.1	CP009011.1	0.033695	61.9	32	35		
			pXAC64	NZ_CP009012.1	CP009012.1	0.064912	61.4		64		
X.axonopodis pv. *citri* strain JX5	XacJX5	Mesophyllic phytopathogen	Chr	NZ_CP009010.1	CP009010.1	5.12	64.8	4,201	4,375	Citrus	ISME J. 2015 Oct;9(10):2128-38. Epub 2015 Feb 17. PMID: 25689023 Zhang et al 2015
			pXAC33	NZ_CP009008.1	CP009008.1	0.033695	61.9	32	35		

			pXAC64	NZ_CP009009.1	CP009009.1	0.064912	61.4	59	64		
X.axonopodis pv. *citri* strain JX6	XacJX6	Mesophyllic phytopathogen	Chr	NZ_CP011827.1	CP011827.1	5.12	64.8	4,192	4,357	Citrus	Direct Submission / Unpublished Zhang et al 2015
			pXAC64	NZ_CP013664.1	CP013664.1	0.064919	61.4	60	64		
			pXAC33	NZ_CP013665.1	CP013665.1	0.033703	61.9	33	35		
X.axonopodis pv. *citri* strain MF20	XacMF20	Mesophyllic phytopathogen	Chr	NZ_CP009007.1	CP009007.1	5.18	64.8	4,234	4,408	Citrus	ISME J. 2015 Oct;9(10):2128-38. Epub 2015 Feb 17. PMID: 25689023 Zhang et al 2015
			pXAC33	NZ_CP009005.1	CP009005.1	0.026933	61.6	26	28		
			pXAC64	NZ_CP009006.1	CP009006.1	0.06492	61.4	61	65		
X.axonopodis pv. *citri* strain MN10	XacMN10	Mesophyllic phytopathogen	Chr	NZ_CP009004.1	CP009004.1	5.12	64.8	4,201	4,376	Citrus	ISME J. 2015 Oct;9(10):2128-38. Epub 2015 Feb 17. PMID: 25689023 Zhang et al 2015
			pXAC33	NZ_CP009002.1	CP009002.1	0.033702	61.9	33	35		
			pXAC64	NZ_CP009003.1	CP009003.1	0.063721	61.3	58	62		
X.axonopodis pv. *citri* strain MN11	XacMN11	Mesophyllic phytopathogen	Chr	NZ_CP009001.1	CP009001.1	5.12	64.8	4,199	4,370	Citrus	ISME J. 2015 Oct;9(10):2128-38. Epub 2015 Feb 17. PMID: 25689023 Zhang et al 2015
			pXAC33	NZ_CP008999.1	CP008999.1	0.033703	61.9	33	35		
			pXAC64	NZ_CP009000.1	CP009000.1	0.063721	61.3	58	62		
X.axonopodis pv. *citri* strain MN12	XacMN12	Mesophyllic phytopathogen	Chr	NZ_CP008998.1	CP008998.1	5.12	64.8	4,205	4,376	Citrus	ISME J. 2015 Oct;9(10):2128-38. Epub 2015 Feb 17. PMID: 25689023 Zhang et al 2015
			pXAC33	NZ_CP008996.1	CP008996.1	0.033703	61.9	33	35		
			pXAC64	NZ_CP008997.1	CP008997.1	0.063721	61.3	58	62		
X.axonopodis pv. *citri* strain NT17	XacNT17	Mesophyllic phytopathogen	Chr	NZ_CP008995.1	CP008995.1	5.18	64.8	4,233	4,406	Citrus	ISME J. 2015 Oct;9(10):2128-38. Epub 2015 Feb 17. PMID: 25689023 Zhang et al 2015
			pXAC33	NZ_CP008993.1	CP008993.1	0.028776	61.7	29	31		
			pXAC64	NZ_CP008994.1	CP008994.1	0.06492	61.4	61	65		
X.axonopodis pv. *citri* strain U16	XacU16	Mesophyllic phytopathogen	Chr	NZ_CP008992.1	CP008992.1	5.12	64.8	4,201	4,374	Citrus	ISME J. 2015 Oct;9(10):2128-38. Epub 2015 Feb 17. PMID: 25689023 Zhang et al 2015
			pXAC33	NZ_CP008990.1	CP008990.1	0.033703	61.9	34	36		
			pXAC64	NZ_CP008991.1	CP008991.1	0.06492	61.4	60	64		
X.axonopodis pv. *citri* strain U17	XacU17	Mesophyllic phytopathogen	Chr	NZ_CP008989.1	CP008989.1	5.12	64.8	4,203	4,371	Citrus	ISME J. 2015 Oct;9(10):2128-38. Epub 2015 Feb 17. PMID: 25689023

			pXAC33	NZ_CP008987.1	CP008987.1	0.033703	61.9	33	35		Zhang et al 2015
			pXAC64	NZ_CP008988.1	CP008988.1	0.06492	61.4	60	64		
X.campestris strain 17	Xc17	Vascular phytopathogen	Chr	NZ_CP011256.1	CP011256.1	5.25	65.4	4,255	4,423	Brassica	Direct Submission / Unpublished<br>Qian et al 2005
X.campestris pv. *campestris* strain B100	XccB100	Vascular phytopathogen	Chr	NC_010688.1	AM920689.1	5.08	65	4,202	4,334	Brassica	J Biotechnol. 2008 Mar 20;134(1-2):33-45. Epub 2008 Jan 20. PMID: 18304669<br>Qian et al 2005
X.campestris pv. *campestris* strain ICMP 21080	XccICMP21080	Vascular phytopathogen	Chr	NZ_CP012145.1	CP012145.1	4.91	65.3	4,022	4,179	Brassica	Genome Announc. 2015 Oct 29;3(5). PMID: 26514760<br>Qian et al 2005
X.campestris pv. *campestris* strain ICMP 4013	XccICMP4013	Vascular phytopathogen	Chr	NZ_CP012146.1	CP012146.1	4.91	65.3	4,007	4,184	Brassica	Genome Announc. 2015 Oct 29;3(5). PMID: 26514760<br>Qian et al 2005
X.fastidiosa strain MUL0034	XfMUL0034	Xylem-limited phytopathogen	Chr	NZ_CP006740.1	CP006740.1	2.64	52	2,204	2,365	Mulberry	Direct Submission / Unpublished<br>Guan et al 2014
			unnamed2	NZ_CP006739.1	CP006739.1	0.024391	49	30	33		
X.fastidiosa subsp. *sandyi* strain Ann-1	XfsandyiAnn1	Xylem-limited phytopathogen	Chr	NZ_CP006696.1	CP006696.1	2.75	52.1	2,339	2,536	Oleander	Direct Submission / Unpublished<br>Bhattacharyya et al 2002
			unnamed1	NZ_CP006697.1	CP006697.1	0.030305	49.5	36	39		
X.fuscans subsp. *fuscans* strain 4834-R	Xfus4834R	Vascular phytopathogen	Chr	NC_022541.1	FO681494.1	4.98	64.8	3,907	4,182	Bean	BMC Genomics. 2013 Nov 6;14:761. PMID: 24195767<br>Darrasse et al 2013
			pla	NC_022539.1	FO681495.1	0.045224	61.3	39	49		
			plb	NC_022540.1	FO681496.1	0.019514	60.6	22	23		
			plc	NC_022542.1	FO681497.1	0.04195	60	36	42		
X.oryzae pv. *oryzicola* strain B8-12	XooB812	Mesophyllic phytopathogen	Chr	NZ_CP011955.1	CP011955.1	4.79	64.1	3,749	4,152	Oryza	Direct Submission / Unpublished<br>Bogdanove et al 2011
X.oryzae pv. *oryzicola* strain BLS279	XooBLS279	Mesophyllic phytopathogen	Chr	NZ_CP011956.1	CP011956.1	4.79	64.1	3,743	4,147	Oryza	Direct Submission / Unpublished<br>Bogdanove et al 2011
X.oryzae pv. *oryzicola* strain BXOR1	XooBXOR1	Mesophyllic phytopathogen	Chr	NZ_CP011957.1	CP011957.1	4.69	64.1	3,660	4,053	Oryza	Direct Submission / Unpublished<br>Bogdanove et al 2011
X.oryzae pv. *oryzicola* strain CFBP2286	XooCFBP2286	Mesophyllic phytopathogen	Chr	NZ_CP011962.1	CP011962.1	4.97	64	3,906	4,317	Oryza	Direct Submission / Unpublished<br>Bogdanove et al 2011
			unnamed	NZ_CP011963.1	CP011963.1	0.036574	60.6	45	47		
X.oryzae pv. *oryzicola* strain CFBP7331	XooCFBP7331	Mesophyllic phytopathogen	Chr	NZ_CP011958.1	CP011958.1	5.01	63.9	3,919	4,377	Oryza	Direct Submission / Unpublished<br>Bogdanove et al 2011
X.oryzae pv. *oryzicola* strain CFBP7341	XooCFBP7341	Mesophyllic phytopathogen	Chr	NZ_CP011959.1	CP011959.1	5.02	63.9	3,934	4,384	Oryza	Direct Submission / Unpublished<br>Bogdanove et al 2011

X.oryzae pv. *oryzicola* strain CFBP7342	XooCFBP7342	Mesophyllic phytopathogen	Chr	NZ_CP007221.1	CP007221.1	5.08	64	4,083	4,537	Oryza	Direct Submission / Unpublished Bogdanove et al 2011
X.oryzae pv. *oryzicola* strain L8	XooL8	Mesophyllic phytopathogen	Chr	NZ_CP011960.1	CP011960.1	4.8	64.1	3,735	4,139	Oryza	Direct Submission / Unpublished Bogdanove et al 2011
X.oryzae pv. *oryzae* strain PXO86	XooPXO86	Vascular phytopathogen	Chr	NZ_CP007166.1	CP007166.1	5.02	63.7	4,178	4,593	Oryza	Direct Submission / Unpublished Qian et al 2005
X.oryzae pv. *oryzicola* strain RS105	XooRS105	Mesophyllic phytopathogen	Chr	NZ_CP011961.1	CP011961.1	4.78	64.1	3,736	4,152	Oryza	Direct Submission / Unpublished Bogdanove et al 2011
X.oryzae pv. *oryzicola* strain YM15	XooYM15	Mesophyllic phytopathogen	Chr	NZ_CP007810.1	CP007810.1	4.43	64.1	3,530	3,887	Oryza	Direct Submission / Unpublished Bogdanove et al 2011
X.sacchari strain R1	XsacchariR1	Biocontrol	Chr	NZ_CP010409.1	CP010409.1	4.5	68.9	3,432	3,642	Oryza	Direct Submission / Unpublished Fang et al 2015
			unnamed	NZ_CP010410.1	CP010410.1	0.508653	69.5	404	441		
X.translucens pv. *undulosa* strain Xtu 4699	Xtu4699	Mesophyllic phytopathogen	Chr	NZ_CP008714.1	CP008714.1	4.56	68.1	3,585	3,783	Wheat	Direct Submission / Unpublished Gardiner et al 2014

Table S2. Members of the seven protein families enriched in Xanthomonadaceae phytopathogens.

NCBI ID	NCBI accession, protein name and species/strain
**Secreted lipase (LipA/LesA)**	
21241272	NP_640854.1 hypothetical protein XAC0501 [Xanthomonas axonopodis pv. citri str. 306]
930138311	AJZ38311.1 hypothetical protein J152_00616 [Xac5208]
470470433	YP_007635001.1 hypothetical protein XAC29_02550 [Xac29_c1]
932267091	AJD67091.1 hypothetical protein J151_00622 [Xanthomonas citri subsp. citri A306]
471266461	YP_007648926.1 secreted lipase [Xanthomonas citri subsp. citri Aw12879]
930243128	AJZ43128.1 Dienelactone hydrolase [XacAW13]
930347744	AJZ47744.1 Dienelactone hydrolase [XacAW14]
930452363	AJZ52363.1 Dienelactone hydrolase [XacAW15]
930565158	AJZ65158.1 Dienelactone hydrolase [XacAW16]
930633842	AJZ33842.1 Dienelactone hydrolase [XacBL18]
930729379	AJZ29379.1 Dienelactone hydrolase [XacFB19]
930824961	AJZ24961.1 Dienelactone hydrolase [XacGD2]
930920535	AJZ20535.1 Dienelactone hydrolase [XacGD3]
931016112	AJZ16112.1 Dienelactone hydrolase [XacJX4]
931111683	AJZ11683.1 Dienelactone hydrolase [XacJX5]
932323755	AKM23755.1 lipase [Xanthomonas citri pv. citri jx-6]
931207212	AJZ07212.1 Dienelactone hydrolase [XacMF20]
931302789	AJZ02789.1 Dienelactone hydrolase [XacMN10]
931498365	AJY98365.1 Dienelactone hydrolase [XacMN11]
931593941	AJY93941.1 Dienelactone hydrolase [XacMN12]
931689470	AJY89470.1 Dienelactone hydrolase [XacNT17]
931785047	AJY85047.1 Dienelactone hydrolase [XacUI6]
931880625	AJY80625.1 Dienelactone hydrolase [XacUI7]
285019895	YP_003377606.1 hypothetical protein XALc_3133 [Xanthomonas albilineans GPE PC73]
346723430	YP_004850099.1 hypothetical protein XACM_0494 [XalfaF1]
932480684	AKC80684.1 lipase [Xanthomonas campestris 17]
66767479	YP_242241.1 hypothetical protein XC_1151 [Xanthomonas campestris pv. campestris str. 8004]
21232388	NP_638305.1 hypothetical protein XCC2957 [Xanthomonas campestris pv. campestris str. ATCC 33913]
932550538	CAP50538.1 Putative secreted lipase [Xanthomonas campestris pv. campestris B100]
932615456	AKS15456.1 lipase [Xanthomonas campestris pv. campestris ICMP 21080]
932719487	AKS19487.1 lipase [Xanthomonas campestris pv. campestris ICMP 4013]
384428952	YP_005638312.1 hypothetical protein XCR_3331 [Xanthomonas campestris pv. raphani 756C]
989122167	XCV0536 CAJ22167 putative secreted lipase [Xcv8510_c1]
932812529	AIC12529.1 lipase [Xylella fastidiosa MUL0034]
932812913	AIC12913.1 lipase [Xylella fastidiosa MUL0034]
932812914	AIC12914.1 lipase [Xylella fastidiosa MUL0034]

932910260	AIC10260.1 lipase [Xylella fastidiosa subsp. sandyi Ann-1]
932910261	AIC10261.1 lipase [Xylella fastidiosa subsp. sandyi Ann-1]
932910717	AIC10717.1 lipase [Xylella fastidiosa subsp. sandyi Ann-1]
549713492	YP_008637355.1 secreted lipase [Xfus4834_c1]
933092053	AKN92053.1 lipase [Xanthomonas oryzae pv. oryzicola B8-12]
384421071	YP_005630431.1 hypothetical protein XOC_4185 [Xanthomonas oryzae pv. oryzicola BLS256]
933195793	AKN95793.1 lipase [Xanthomonas oryzae pv. oryzicola BLS279]
933299526	AKN99526.1 lipase [Xanthomonas oryzae pv. oryzicola BXOR1]
933321296	AKO21296.1 lipase [Xanthomonas oryzae pv. oryzicola CFBP2286]
933405935	AKO05935.1 lipase [Xanthomonas oryzae pv. oryzicola CFBP7331]
933505935	AKO05935.1 lipase [Xanthomonas oryzae pv. oryzicola CFBP7341]
933686168	AJQ86168.1 lipase [Xanthomonas oryzae pv. oryzicola CFBP7342]
933711014	AKO11014.1 lipase [Xanthomonas oryzae pv. oryzicola L8]
933885004	AJQ85004.1 lipase [Xanthomonas oryzae pv. oryzae PXO86]
933911014	AKO11014.1 lipase [Xanthomonas oryzae pv. oryzicola RS105]
934065497	AKK65497.1 lipase [Xanthomonas oryzae pv. oryzicola YM15]
58580149	YP_199165.1 hypothetical protein XOO0526 [Xanthomonas oryzae pv. oryzae KACC 10331]
84622147	YP_449519.1 hypothetical protein XOO_0490 [Xanthomonas oryzae pv. oryzae MAFF 311018]
188578865	YP_001915794.1 hypothetical protein PXO_02951 [Xanthomonas oryzae pv. oryzae PXO99A]
935546583	AJC46583.1 lipase [Xanthomonas sacchari R1]
935666103	AKK66103.1 lipase [Xanthomonas translucens pv. undulosa Xtu 4699]
15836959	NP_297647.1 hypothetical protein XF0357 [Xylella fastidiosa 9a5c]
15836960	NP_297648.1 hypothetical protein XF0358 [Xylella fastidiosa 9a5c]
15838742	NP_299430.1 hypothetical protein XF2151 [Xylella fastidiosa 9a5c]
386083129	YP_005999411.1 putative secreted lipase [Xylella fastidiosa subsp. fastidiosa GB514]
386083642	YP_005999924.1 putative secreted lipase [Xylella fastidiosa subsp. fastidiosa GB514]
386083643	YP_005999925.1 putative secreted lipase [Xylella fastidiosa subsp. fastidiosa GB514]
170730482	YP_001775915.1 hypothetical protein Xfasm12_1355 [Xylella fastidiosa M12]
170730948	YP_001776381.1 hypothetical protein Xfasm12_1869 [Xylella fastidiosa M12]
182681826	YP_001829986.1 putative secreted lipase [Xylella fastidiosa M23]
182682312	YP_001830472.1 putative secreted lipase [Xylella fastidiosa M23]
182682313	YP_001830473.1 putative secreted lipase [Xylella fastidiosa M23]
28199099	NP_779413.1 hypothetical protein PD1211 [Xylella fastidiosa Temecula1]
28199577	NP_779891.1 hypothetical protein PD1702 [Xylella fastidiosa Temecula1]
28199578	NP_779892.1 hypothetical protein PD1703 [Xylella fastidiosa Temecula1]
Number of proteins = 71	
Gblocks 418/451 (92%)[a]	
**Alpha-L-fucosidase (FucA1)**	
21242059	NP_641641.1 hypothetical protein XAC1306 [Xanthomonas axonopodis pv. citri str. 306]
930139117	AJZ39117.1 Alpha-L-fucosidase [Xac5208]

470471206	YP_007635774.1 Alpha-L-fucosidase [Xac29_c1]
932267896	AJD67896.1 alpha-L-fucosidase [Xanthomonas citri subsp. citri A306]
471268551	YP_007651016.1 Alpha-L-fucosidase [Xanthomonas citri subsp. citri Aw12879]
930245135	AJZ45135.1 Alpha-L-fucosidase [XacAW13]
930349753	AJZ49753.1 Alpha-L-fucosidase [XacAW14]
930454373	AJZ54373.1 Alpha-L-fucosidase [XacAW15]
930567166	AJZ67166.1 Alpha-L-fucosidase [XacAW16]
930634647	AJZ34647.1 Alpha-L-fucosidase [XacBL18]
930730184	AJZ30184.1 Alpha-L-fucosidase [XacFB19]
930825766	AJZ25766.1 Alpha-L-fucosidase [XacGD2]
930921340	AJZ21340.1 Alpha-L-fucosidase [XacGD3]
931016917	AJZ16917.1 Alpha-L-fucosidase [XacJX4]
931112488	AJZ12488.1 Alpha-L-fucosidase [XacJX5]
932324473	AKM24473.1 alpha-L-fucosidase [Xanthomonas citri pv. citri jx-6]
931208017	AJZ08017.1 Alpha-L-fucosidase [XacMF20]
931303594	AJZ03594.1 Alpha-L-fucosidase [XacMN10]
931499170	AJY99170.1 Alpha-L-fucosidase [XacMN11]
931594746	AJY94746.1 Alpha-L-fucosidase [XacMN12]
931690275	AJY90275.1 Alpha-L-fucosidase [XacNT17]
931785851	AJY85851.1 Alpha-L-fucosidase [XacUI6]
931881429	AJY81429.1 Alpha-L-fucosidase [XacUI7]
285018989	YP_003376700.1 alpha-l-fucosidase [Xanthomonas albilineans GPE PC73]
346724202	YP_004850871.1 Alpha-L-fucosidase [XalfaF1]
932477556	AKC77556.1 alpha-L-fucosidase [Xanthomonas campestris 17]
66769296	YP_244058.1 hypothetical protein XC_2990 [Xanthomonas campestris pv. campestris str. 8004]
21230708	NP_636625.1 hypothetical protein XCC1251 [Xanthomonas campestris pv. campestris str. ATCC 33913]
932552415	CAP52415.1 exported alpha-L-fucosidase [Xanthomonas campestris pv. campestris B100]
932616941	AKS16941.1 alpha-L-fucosidase [Xanthomonas campestris pv. campestris ICMP 21080]
932720958	AKS20958.1 alpha-L-fucosidase [Xanthomonas campestris pv. campestris ICMP 4013]
384427165	YP_005636523.1 F5-8 type C domain protein [Xanthomonas campestris pv. raphani 756C]
989122988	XCV1357 CAJ22988 conserved hypothetical protein [Xcv8510_c1]
932814110	AIC14110.1 alpha-L-fucosidase [Xylella fastidiosa MUL0034]
932910971	AIC10971.1 alpha-L-fucosidase [Xylella fastidiosa subsp. sandyi Ann-1]
549715993	YP_008639856.1 putative alpha-L-fucosidase [Xfus4834_c1]
933094077	AKN94077.1 alpha-L-fucosidase [Xanthomonas oryzae pv. oryzicola B8-12]
384420107	YP_005629467.1 F5-8 type C domain protein [Xanthomonas oryzae pv. oryzicola BLS256]
933197755	AKN97755.1 alpha-L-fucosidase [Xanthomonas oryzae pv. oryzicola BLS279]
933200213	AKO00213.1 alpha-L-fucosidase [Xanthomonas oryzae pv. oryzicola BXOR1]
933320533	AKO20533.1 alpha-L-fucosidase [Xanthomonas oryzae pv. oryzicola CFBP2286]
933403971	AKO03971.1 alpha-L-fucosidase [Xanthomonas oryzae pv. oryzicola CFBP7331]

933503971	AKO03971.1 alpha-L-fucosidase [Xanthomonas oryzae pv. oryzicola CFBP7341]
933686836	AJQ86836.1 alpha-L-fucosidase [Xanthomonas oryzae pv. oryzicola CFBP7342]
933713025	AKO13025.1 alpha-L-fucosidase [Xanthomonas oryzae pv. oryzicola L8]
933883801	AJQ83801.1 alpha-L-fucosidase [Xanthomonas oryzae pv. oryzae PXO86]
933913025	AKO13025.1 alpha-L-fucosidase [Xanthomonas oryzae pv. oryzicola RS105]
934064788	AKK64788.1 alpha-L-fucosidase [Xanthomonas oryzae pv. oryzicola YM15]
58581458	YP_200474.1 hypothetical protein XOO1835 [Xanthomonas oryzae pv. oryzae KACC 10331]
84623388	YP_450760.1 hypothetical protein XOO_1731 [Xanthomonas oryzae pv. oryzae MAFF 311018]
188577303	YP_001914232.1 F5/8 type C domain-containing protein [Xanthomonas oryzae pv. oryzae PXO99A]
935544489	AJC44489.1 alpha-L-fucosidase [Xanthomonas sacchari R1]
935545484	AJC45484.1 alpha-L-fucosidase [Xanthomonas sacchari R1]
935667025	AKK67025.1 alpha-L-fucosidase [Xanthomonas translucens pv. undulosa Xtu 4699]
15836711	NP_297399.1 hypothetical protein XF0106 [Xylella fastidiosa 9a5c]
386084155	YP_006000437.1 Alpha-L-fucosidase [Xylella fastidiosa subsp. fastidiosa GB514]
170729323	YP_001774756.1 alpha-L-fucosidase [Xylella fastidiosa M12]
182680643	YP_001828803.1 alpha-L-fucosidase [Xylella fastidiosa M23]
28198017	NP_778331.1 hypothetical protein PD0080 [Xylella fastidiosa Temecula1]
Number of proteins = 59	
Gblocks 638/793 (80%)[a]	
**Alpha-L-fucosidase (NixE)**	
21243799	NP_643381.1 alpha-L-fucosidase [Xanthomonas axonopodis pv. citri str. 306]
930140890	AJZ40890.1 Alpha-L-fucosidase [Xac5208]
470472941	YP_007637509.1 alpha-L-fucosidase [Xac29_c1]
932269669	AJD69669.1 alpha-L-fucosidase [Xanthomonas citri subsp. citri A306]
471268867	YP_007651332.1 Alpha-L-fucosidase [Xanthomonas citri subsp. citri Aw12879]
930245437	AJZ45437.1 Alpha-L-fucosidase [XacAW13]
930350055	AJZ50055.1 Alpha-L-fucosidase [XacAW14]
930454675	AJZ54675.1 Alpha-L-fucosidase [XacAW15]
930567468	AJZ67468.1 Alpha-L-fucosidase [XacAW16]
930636421	AJZ36421.1 Alpha-L-fucosidase [XacBL18]
930731958	AJZ31958.1 Alpha-L-fucosidase [XacFB19]
930827520	AJZ27520.1 Alpha-L-fucosidase [XacGD2]
930923096	AJZ23096.1 Alpha-L-fucosidase [XacGD3]
931018669	AJZ18669.1 Alpha-L-fucosidase [XacJX4]
931114243	AJZ14243.1 Alpha-L-fucosidase [XacJX5]
932326044	AKM26044.1 alpha-L-fucosidase [Xanthomonas citri pv. citri jx-6]
931209790	AJZ09790.1 Alpha-L-fucosidase [XacMF20]
931305350	AJZ05350.1 Alpha-L-fucosidase [XacMN10]
931400923	AJZ00923.1 Alpha-L-fucosidase [XacMN11]
931596498	AJY96498.1 Alpha-L-fucosidase [XacMN12]

931692050	AJY92050.1 Alpha-L-fucosidase [XacNT17]
931787607	AJY87607.1 Alpha-L-fucosidase [XacUI6]
931883181	AJY83181.1 Alpha-L-fucosidase [XacUI7]
285018980	YP_003376691.1 alpha-l-fucosidase [Xanthomonas albilineans GPE PC73]
346725875	YP_004852544.1 alpha-L-fucosidase [XalfaF1]
932479041	AKC79041.1 alpha-L-fucosidase [Xanthomonas campestris 17]
66767548	YP_242310.1 alpha-L-fucosidase [Xanthomonas campestris pv. campestris str. 8004]
21232319	NP_638236.1 alpha-L-fucosidase [Xanthomonas campestris pv. campestris str. ATCC 33913]
932550614	CAP50614.1 exported alpha-L-fucosidase [Xanthomonas campestris pv. campestris B100]
932615521	AKS15521.1 alpha-L-fucosidase [Xanthomonas campestris pv. campestris ICMP 21080]
932719549	AKS19549.1 alpha-L-fucosidase [Xanthomonas campestris pv. campestris ICMP 4013]
384428891	YP_005638251.1 alpha-L-fucosidase [Xanthomonas campestris pv. raphani 756C]
989124938	XCV3207 CAJ24938 alpha-L-fucosidase [Xcv8510_c1]
932813376	AIC13376.1 alpha-L-fucosidase [Xylella fastidiosa MUL0034]
932909177	AIC09177.1 alpha-L-fucosidase [Xylella fastidiosa subsp. sandyi Ann-1]
549714475	YP_008638338.1 alpha-L-fucosidase [Xfus4834_c1]
933094115	AKN94115.1 alpha-L-fucosidase [Xanthomonas oryzae pv. oryzicola B8-12]
384420159	YP_005629519.1 alpha-L-fucosidase [Xanthomonas oryzae pv. oryzicola BLS256]
933197793	AKN97793.1 alpha-L-fucosidase [Xanthomonas oryzae pv. oryzicola BLS279]
933200176	AKO00176.1 alpha-L-fucosidase [Xanthomonas oryzae pv. oryzicola BXOR1]
933320569	AKO20569.1 alpha-L-fucosidase [Xanthomonas oryzae pv. oryzicola CFBP2286]
933403930	AKO03930.1 alpha-L-fucosidase [Xanthomonas oryzae pv. oryzicola CFBP7331]
933503930	AKO03930.1 alpha-L-fucosidase [Xanthomonas oryzae pv. oryzicola CFBP7341]
933686793	AJQ86793.1 alpha-L-fucosidase [Xanthomonas oryzae pv. oryzicola CFBP7342]
933713067	AKO13067.1 alpha-L-fucosidase [Xanthomonas oryzae pv. oryzicola L8]
933883844	AJQ83844.1 alpha-L-fucosidase [Xanthomonas oryzae pv. oryzae PXO86]
933913067	AKO13067.1 alpha-L-fucosidase [Xanthomonas oryzae pv. oryzicola RS105]
934064827	AKK64827.1 alpha-L-fucosidase [Xanthomonas oryzae pv. oryzicola YM15]
58581406	YP_200422.1 alpha-L-fucosidase [Xanthomonas oryzae pv. oryzae KACC 10331]
84623343	YP_450715.1 alpha-L-fucosidase [Xanthomonas oryzae pv. oryzae MAFF 311018]
188577354	YP_001914283.1 alpha-L-fucosidase [Xanthomonas oryzae pv. oryzae PXO99A]
935544517	AJC44517.1 alpha-L-fucosidase [Xanthomonas sacchari R1]
935668458	AKK68458.1 alpha-L-fucosidase [Xanthomonas translucens pv. undulosa Xtu 4699]
15839303	NP_299991.1 alpha-L-fucosidase [Xylella fastidiosa 9a5c]
386084013	YP_006000295.1 Alpha-L-fucosidase [Xylella fastidiosa subsp. fastidiosa GB514]
170731308	YP_001776741.1 alpha-L-fucosidase [Xylella fastidiosa M12]
182682679	YP_001830839.1 alpha-L-fucosidase [Xylella fastidiosa M23]
28199927	NP_780241.1 alpha-L-fucosidase [Xylella fastidiosa Temecula1]
Number of proteins = 58	
Gblocks 559/626 (89%)[a]	

**VirK protein (VirK)**	
21241208	NP_640790.1 VirK protein [Xanthomonas axonopodis pv. citri str. 306]
930138249	AJZ38249.1 hypothetical protein J152_00552 [Xac5208]
470470370	YP_007634938.1 VirK protein [Xac29_c1]
932267029	AJD67029.1 hypothetical protein J151_00558 [Xanthomonas citri subsp. citri A306]
471266395	YP_007648860.1 Hypothetical Protein XCAW_00846 [Xanthomonas citri subsp. citri Aw12879]
930243065	AJZ43065.1 VirK protein [XacAW13]
930347681	AJZ47681.1 VirK protein [XacAW14]
930452300	AJZ52300.1 VirK protein [XacAW15]
930565095	AJZ65095.1 VirK protein [XacAW16]
930633780	AJZ33780.1 VirK protein [XacBL18]
930729316	AJZ29316.1 VirK protein [XacFB19]
930824899	AJZ24899.1 VirK protein [XacGD2]
930920473	AJZ20473.1 VirK protein [XacGD3]
931016049	AJZ16049.1 VirK protein [XacJX4]
931111620	AJZ11620.1 VirK protein [XacJX5]
932323697	AKM23697.1 hypothetical protein AB890_02275 [Xanthomonas citri pv. citri jx-6]
931207150	AJZ07150.1 VirK protein [XacMF20]
931302727	AJZ02727.1 VirK protein [XacMN10]
931498303	AJY98303.1 VirK protein [XacMN11]
931593879	AJY93879.1 VirK protein [XacMN12]
931689408	AJY89408.1 VirK protein [XacNT17]
931784985	AJY84985.1 VirK protein [XacUI6]
931880563	AJY80563.1 VirK protein [XacUI7]
285018768	YP_003376479.1 virk; protein [Xanthomonas albilineans GPE PC73]
346723365	YP_004850034.1 VirK protein [XalfaF1]
932480551	AKC80551.1 hypothetical protein XB05_18665 [Xanthomonas campestris 17]
66766773	YP_241535.1 VirK protein [Xanthomonas campestris pv. campestris str. 8004]
21229895	NP_635812.1 VirK protein [Xanthomonas campestris pv. campestris str. ATCC 33913]
932549781	CAP49781.1 putative exported protein [Xanthomonas campestris pv. campestris B100]
932614831	AKS14831.1 hypothetical protein AEA00_02125 [Xanthomonas campestris pv. campestris ICMP 21080]
932718848	AKS18848.1 hypothetical protein AEA01_02115 [Xanthomonas campestris pv. campestris ICMP 4013]
384429709	YP_005639070.1 VirK protein [Xanthomonas campestris pv. raphani 756C]
989122093	XCV0462 CAJ22093 VirK protein [Xcv8510_c1]
932812398	AIC12398.1 VirK protein [Xylella fastidiosa MUL0034]
932910345	AIC10345.1 VirK protein [Xylella fastidiosa subsp. sandyi Ann-1]
549713434	YP_008637297.1 VirK protein [Xfus4834_c1]
933094783	AKN94783.1 hypothetical protein ACU13_18985 [Xanthomonas oryzae pv. oryzicola B8-12]
384417510	YP_005626870.1 VirK protein [Xanthomonas oryzae pv. oryzicola BLS256]
933198509	AKN98509.1 hypothetical protein ACU10_18905 [Xanthomonas oryzae pv. oryzicola BLS279]

933202170	AKO02170.1 hypothetical protein ACU15_18465 [Xanthomonas oryzae pv. oryzicola BXOR1]
933318448	AKO18448.1 hypothetical protein ACU11_02055 [Xanthomonas oryzae pv. oryzicola CFBP2286]
933403173	AKO03173.1 hypothetical protein ACU16_02225 [Xanthomonas oryzae pv. oryzicola CFBP7331]
933503173	AKO03173.1 hypothetical protein ACU16_02225 [Xanthomonas oryzae pv. oryzicola CFBP7341]
933689400	AJQ89400.1 hypothetical protein BE73_22035 [Xanthomonas oryzae pv. oryzicola CFBP7342]
933713733	AKO13733.1 hypothetical protein ACU14_18920 [Xanthomonas oryzae pv. oryzicola L8]
933881330	AJQ81330.1 hypothetical protein AZ54_00650 [Xanthomonas oryzae pv. oryzae PXO86]
933913733	AKO13733.1 hypothetical protein ACU14_18920 [Xanthomonas oryzae pv. oryzicola RS105]
934062683	AKK62683.1 hypothetical protein FE36_01665 [Xanthomonas oryzae pv. oryzicola YM15]
58579754	YP_198770.1 VirK protein [Xanthomonas oryzae pv. oryzae KACC 10331]
84621700	YP_449072.1 VirK protein [Xanthomonas oryzae pv. oryzae MAFF 311018]
188574397	YP_001911326.1 VirK protein [Xanthomonas oryzae pv. oryzae PXO99A]
935545191	AJC45191.1 hypothetical protein SB85_04925 [Xanthomonas sacchari R1]
935668166	AKK68166.1 hypothetical protein FD63_12115 [Xanthomonas translucens pv. undulosa Xtu 4699]
15838539	NP_299227.1 VirK protein [Xylella fastidiosa 9a5c]
386084955	YP_006001237.1 VirK family protein [Xylella fastidiosa subsp. fastidiosa GB514]
170730189	YP_001775622.1 VirK protein [Xylella fastidiosa M12]
182681452	YP_001829612.1 VirK family protein [Xylella fastidiosa M23]
28198760	NP_779074.1 VirK protein [Xylella fastidiosa Temecula1]
Number of proteins = 58	
Gblocks 142/174 (81%)[a]	
**Chorismate mutase (PheA)**	
21244372	NP_643954.1 chorismate mutase [Xanthomonas axonopodis pv. citri str. 306]
930141457	AJZ41457.1 Chorismate mutase [Xac5208]
470473507	YP_007638075.1 chorismate mutase [Xac29_c1]
932270238	AJD70238.1 chorismate mutase [Xanthomonas citri subsp. citri A306]
471269846	YP_007652311.1 Chorismate mutase [Xanthomonas citri subsp. citri Aw12879]
930246402	AJZ46402.1 chorismate mutase, putative [XacAW13]
930351022	AJZ51022.1 chorismate mutase, putative [XacAW14]
930455643	AJZ55643.1 chorismate mutase, putative [XacAW15]
930568433	AJZ68433.1 chorismate mutase, putative [XacAW16]
930636990	AJZ36990.1 chorismate mutase, putative [XacBL18]
930732526	AJZ32526.1 chorismate mutase, putative [XacFB19]
930828085	AJZ28085.1 chorismate mutase, putative [XacGD2]
930923661	AJZ23661.1 chorismate mutase, putative [XacGD3]
931019233	AJZ19233.1 chorismate mutase, putative [XacJX4]
931114809	AJZ14809.1 chorismate mutase, putative [XacJX5]
932326544	AKM26544.1 chorismate mutase [Xanthomonas citri pv. citri jx-6]
931210358	AJZ10358.1 chorismate mutase, putative [XacMF20]
931305917	AJZ05917.1 chorismate mutase, putative [XacMN10]

931401489	AJZ01489.1 chorismate mutase, putative [XacMN11]
931597065	AJY97065.1 chorismate mutase, putative [XacMN12]
931692618	AJY92618.1 chorismate mutase, putative [XacNT17]
931788173	AJY88173.1 chorismate mutase, putative [XacUI6]
931883747	AJY83747.1 chorismate mutase, putative [XacUI7]
285019664	YP_003377375.1 chorismate mutase precursor [Xanthomonas albilineans GPE PC73]
346726415	YP_004853084.1 chorismate mutase [XalfaF1]
932481391	AKC81391.1 chorismate mutase [Xanthomonas campestris 17]
66769975	YP_244737.1 chorismate mutase [Xanthomonas campestris pv. campestris str. 8004]
21230031	NP_635948.1 chorismate mutase [Xanthomonas campestris pv. campestris str. ATCC 33913]
932553159	CAP53159.1 Monofunctional chorismate mutase precursor [Xanthomonas campestris pv. campestris B100]
932618426	AKS18426.1 chorismate mutase [Xanthomonas campestris pv. campestris ICMP 21080]
932722436	AKS22436.1 chorismate mutase [Xanthomonas campestris pv. campestris ICMP 4013]
384426387	YP_005635744.1 chorismate mutase-prephenate dehydratase [Xanthomonas campestris pv. raphani 756C]
989125496	XCV3765 CAJ25496 Chorismate mutase precursor [Xcv8510_c1]
932811993	AIC11993.1 chorismate mutase [Xylella fastidiosa MUL0034]
932909550	AIC09550.1 chorismate mutase [Xylella fastidiosa subsp. sandyi Ann-1]
549716313	YP_008640176.1 putative chorismate mutase [Xfus4834_c1]
933094590	AKN94590.1 chorismate mutase [Xanthomonas oryzae pv. oryzicola B8-12]
384420809	YP_005630169.1 chorismate mutase, prephenate dehydratase [Xanthomonas oryzae pv. oryzicola BLS256]
933198313	AKN98313.1 chorismate mutase [Xanthomonas oryzae pv. oryzicola BLS279]
933299696	AKN99696.1 chorismate mutase [Xanthomonas oryzae pv. oryzicola BXOR1]
933321103	AKO21103.1 chorismate mutase [Xanthomonas oryzae pv. oryzicola CFBP2286]
933403438	AKO03438.1 chorismate mutase [Xanthomonas oryzae pv. oryzicola CFBP7331]
933503438	AKO03438.1 chorismate mutase [Xanthomonas oryzae pv. oryzicola CFBP7341]
933686390	AJQ86390.1 chorismate mutase [Xanthomonas oryzae pv. oryzicola CFBP7342]
933713539	AKO13539.1 chorismate mutase [Xanthomonas oryzae pv. oryzicola L8]
933884788	AJQ84788.1 chorismate mutase [Xanthomonas oryzae pv. oryzae PXO86]
933913539	AKO13539.1 chorismate mutase [Xanthomonas oryzae pv. oryzicola RS105]
934065315	AKK65315.1 chorismate mutase [Xanthomonas oryzae pv. oryzicola YM15]
58580357	YP_199373.1 chorismate mutase [Xanthomonas oryzae pv. oryzae KACC 10331]
84622327	YP_449699.1 chorismate mutase [Xanthomonas oryzae pv. oryzae MAFF 311018]
188578708	YP_001915637.1 chorismate mutase [Xanthomonas oryzae pv. oryzae PXO99A]
935547571	AJC47571.1 chorismate mutase [Xanthomonas sacchari R1]
935669031	AKK69031.1 chorismate mutase [Xanthomonas translucens pv. undulosa Xtu 4699]
15837743	NP_298431.1 chorismate mutase [Xylella fastidiosa 9a5c]
386084504	YP_006000786.1 chorismate mutase [Xylella fastidiosa subsp. fastidiosa GB514]
170729689	YP_001775122.1 chorismate mutase [Xylella fastidiosa M12]
182680983	YP_001829143.1 chorismate mutase [Xylella fastidiosa M23]

28198342	NP_778656.1 chorismate mutase [Xylella fastidiosa Temecula1]
Number of proteins = 58	
Gblocks 189/201 (94%)[a]	
**Glycosyl hydrolase (NixF)**	
21243800	NP_643382.1 hypothetical protein XAC3073 [Xanthomonas axonopodis pv. citri str. 306]
930140891	AJZ40891.1 putative glycosyl hydrolase [Xac5208]
470472942	YP_007637510.1 hypothetical protein XAC29_15630 [Xac29_c1]
932269670	AJD69670.1 putative glycosyl hydrolase [Xanthomonas citri subsp. citri A306]
471268868	YP_007651333.1 glycosyl hydrolase [Xanthomonas citri subsp. citri Aw12879]
930245438	AJZ45438.1 putative glycosyl hydrolase [XacAW13]
930350056	AJZ50056.1 putative glycosyl hydrolase [XacAW14]
930454676	AJZ54676.1 putative glycosyl hydrolase [XacAW15]
930567469	AJZ67469.1 putative glycosyl hydrolase [XacAW16]
930636422	AJZ36422.1 putative glycosyl hydrolase [XacBL18]
930731959	AJZ31959.1 putative glycosyl hydrolase [XacFB19]
930827521	AJZ27521.1 putative glycosyl hydrolase [XacGD2]
930923097	AJZ23097.1 putative glycosyl hydrolase [XacGD3]
931018670	AJZ18670.1 putative glycosyl hydrolase [XacJX4]
931114244	AJZ14244.1 putative glycosyl hydrolase [XacJX5]
932326045	AKM26045.1 glycosyl hydrolase [Xanthomonas citri pv. citri jx-6]
931209791	AJZ09791.1 putative glycosyl hydrolase [XacMF20]
931305351	AJZ05351.1 putative glycosyl hydrolase [XacMN10]
931400924	AJZ00924.1 putative glycosyl hydrolase [XacMN11]
931596499	AJY96499.1 putative glycosyl hydrolase [XacMN12]
931692051	AJY92051.1 putative glycosyl hydrolase [XacNT17]
931787608	AJY87608.1 putative glycosyl hydrolase [XacUI6]
931883182	AJY83182.1 putative glycosyl hydrolase [XacUI7]
285018981	YP_003376692.1 glycosyl hydrolases family 18 [Xanthomonas albilineans GPE PC73]
346725876	YP_004852545.1 glycosyl hydrolase [XalfaF1]
932479042	AKC79042.1 glycosyl hydrolase [Xanthomonas campestris 17]
66767547	YP_242309.1 hypothetical protein XC_1220 [Xanthomonas campestris pv. campestris str. 8004]
21232320	NP_638237.1 hypothetical protein XCC2889 [Xanthomonas campestris pv. campestris str. ATCC 33913]
932550613	CAP50613.1 exported putative chitinase [Xanthomonas campestris pv. campestris B100]
932615520	AKS15520.1 glycosyl hydrolase [Xanthomonas campestris pv. campestris ICMP 21080]
932719548	AKS19548.1 glycosyl hydrolase [Xanthomonas campestris pv. campestris ICMP 4013]
384428892	YP_005638252.1 glycosyl hydrolase family protein [Xanthomonas campestris pv. raphani 756C]
989124939	XCV3208 CAJ24939 putative glycoside hydrolase family 18 protein [Xcv8510_c1]
932811853	AIC11853.1 glycosyl hydrolase [Xylella fastidiosa MUL0034]
932910774	AIC10774.1 glycosyl hydrolase [Xylella fastidiosa subsp. sandyi Ann-1]
549714474	YP_008638337.1 putative endo-beta-N-acetylglucosaminidase [Xfus4834_c1]

933094116	AKN94116.1 glycosyl hydrolase [Xanthomonas oryzae pv. oryzicola B8-12]
384420160	YP_005629520.1 glycosyl hydrolase family protein [Xanthomonas oryzae pv. oryzicola BLS256]
933197794	AKN97794.1 glycosyl hydrolase [Xanthomonas oryzae pv. oryzicola BLS279]
933200175	AKO00175.1 glycosyl hydrolase [Xanthomonas oryzae pv. oryzicola BXOR1]
933320570	AKO20570.1 glycosyl hydrolase [Xanthomonas oryzae pv. oryzicola CFBP2286]
933403929	AKO03929.1 glycosyl hydrolase [Xanthomonas oryzae pv. oryzicola CFBP7331]
933503929	AKO03929.1 glycosyl hydrolase [Xanthomonas oryzae pv. oryzicola CFBP7341]
933686792	AJQ86792.1 glycosyl hydrolase [Xanthomonas oryzae pv. oryzicola CFBP7342]
933713068	AKO13068.1 glycosyl hydrolase [Xanthomonas oryzae pv. oryzicola L8]
933883845	AJQ83845.1 glycosyl hydrolase [Xanthomonas oryzae pv. oryzae PXO86]
933913068	AKO13068.1 glycosyl hydrolase [Xanthomonas oryzae pv. oryzicola RS105]
934064828	AKK64828.1 glycosyl hydrolase [Xanthomonas oryzae pv. oryzicola YM15]
58581405	YP_200421.1 hypothetical protein XOO1782 [Xanthomonas oryzae pv. oryzae KACC 10331]
84623342	YP_450714.1 hypothetical protein XOO_1685 [Xanthomonas oryzae pv. oryzae MAFF 311018]
188577355	YP_001914284.1 glycosyl hydrolase family protein [Xanthomonas oryzae pv. oryzae PXO99A]
935544516	AJC44516.1 glycosyl hydrolase [Xanthomonas sacchari R1]
935668459	AKK68459.1 glycosyl hydrolase [Xanthomonas translucens pv. undulosa Xtu 4699]
15837450	NP_298138.1 hypothetical protein XF0848 [Xylella fastidiosa 9a5c]
386083776	YP_006000058.1 glycoside hydrolase family protein [Xylella fastidiosa subsp. fastidiosa GB514]
170731069	YP_001776502.1 hypothetical protein Xfasm12_2002 [Xylella fastidiosa M12]
182682440	YP_001830600.1 glycoside hydrolase family protein [Xylella fastidiosa M23]
28199696	NP_780010.1 hypothetical protein PD1826 [Xylella fastidiosa Temecula1]
Number of proteins = 58	
Gblocks 351/359 (97%)[a]	
**Beta-galactosidase (NixL)**	
21243811	NP_643393.1 beta-galactosidase [Xanthomonas axonopodis pv. citri str. 306]
930140902	AJZ40902.1 Beta-galactosidase [Xac5208]
470472953	YP_007637521.1 beta-galactosidase [Xac29_c1]
932269681	AJD69681.1 beta-galactosidase [Xanthomonas citri subsp. citri A306]
471268879	YP_007651344.1 Beta-galactosidase [Xanthomonas citri subsp. citri Aw12879]
930245449	AJZ45449.1 Beta-galactosidase [XacAW13]
930350067	AJZ50067.1 Beta-galactosidase [XacAW14]
930454687	AJZ54687.1 Beta-galactosidase [XacAW15]
930567480	AJZ67480.1 Beta-galactosidase [XacAW16]
930636433	AJZ36433.1 Beta-galactosidase [XacBL18]
930731970	AJZ31970.1 Beta-galactosidase [XacFB19]
930827532	AJZ27532.1 Beta-galactosidase [XacGD2]
930923108	AJZ23108.1 Beta-galactosidase [XacGD3]
931018681	AJZ18681.1 Beta-galactosidase [XacJX4]
931114255	AJZ14255.1 Beta-galactosidase [XacJX5]

932326055	AKM26055.1 beta-galactosidase [Xanthomonas citri pv. citri jx-6]
931209802	AJZ09802.1 Beta-galactosidase [XacMF20]
931305362	AJZ05362.1 Beta-galactosidase [XacMN10]
931400935	AJZ00935.1 Beta-galactosidase [XacMN11]
931596510	AJY96510.1 Beta-galactosidase [XacMN12]
931692062	AJY92062.1 Beta-galactosidase [XacNT17]
931787619	AJY87619.1 Beta-galactosidase [XacUI6]
931883193	AJY83193.1 Beta-galactosidase [XacUI7]
285018987	YP_003376698.1 beta-galactosidase [Xanthomonas albilineans GPE PC73]
346725882	YP_004852551.1 beta-galactosidase [XalfaF1]
932479048	AKC79048.1 beta-galactosidase [Xanthomonas campestris 17]
66767541	YP_242303.1 beta-galactosidase [Xanthomonas campestris pv. campestris str. 8004]
21232326	NP_638243.1 beta-galactosidase [Xanthomonas campestris pv. campestris str. ATCC 33913]
932550607	CAP50607.1 exported beta-galactosidase [Xanthomonas campestris pv. campestris B100]
932615516	AKS15516.1 beta-galactosidase [Xanthomonas campestris pv. campestris ICMP 21080]
932719544	AKS19544.1 beta-galactosidase [Xanthomonas campestris pv. campestris ICMP 4013]
384428898	YP_005638258.1 beta-galactosidase [Xanthomonas campestris pv. raphani 756C]
989124945	XCV3214 CAJ24945 beta-galactosidase [Xcv8510_c1]
932811847	AIC11847.1 beta-galactosidase [Xylella fastidiosa MUL0034]
932910780	AIC10780.1 beta-galactosidase [Xylella fastidiosa subsp. sandyi Ann-1]
549714463	YP_008638326.1 beta-galactosidase [Xfus4834_c1]
933094124	AKN94124.1 beta-galactosidase [Xanthomonas oryzae pv. oryzicola B8-12]
384420175	YP_005629535.1 beta-galactosidase [Xanthomonas oryzae pv. oryzicola BLS256]
933197801	AKN97801.1 beta-galactosidase [Xanthomonas oryzae pv. oryzicola BLS279]
933200167	AKO00167.1 beta-galactosidase [Xanthomonas oryzae pv. oryzicola BXOR1]
933320579	AKO20579.1 beta-galactosidase [Xanthomonas oryzae pv. oryzicola CFBP2286]
933403921	AKO03921.1 beta-galactosidase [Xanthomonas oryzae pv. oryzicola CFBP7331]
933503921	AKO03921.1 beta-galactosidase [Xanthomonas oryzae pv. oryzicola CFBP7341]
933686783	AJQ86783.1 beta-galactosidase [Xanthomonas oryzae pv. oryzicola CFBP7342]
933713076	AKO13076.1 beta-galactosidase [Xanthomonas oryzae pv. oryzicola L8]
933883858	AJQ83858.1 beta-galactosidase [Xanthomonas oryzae pv. oryzae PXO86]
933913076	AKO13076.1 beta-galactosidase [Xanthomonas oryzae pv. oryzicola RS105]
934064835	AKK64835.1 beta-galactosidase [Xanthomonas oryzae pv. oryzicola YM15]
58581392	YP_200408.1 beta-galactosidase [Xanthomonas oryzae pv. oryzae KACC 10331]
84623327	YP_450699.1 beta-galactosidase [Xanthomonas oryzae pv. oryzae MAFF 311018]
188577369	YP_001914298.1 beta-galactosidase [Xanthomonas oryzae pv. oryzae PXO99A]
935544505	AJC44505.1 beta-galactosidase [Xanthomonas sacchari R1]
935668467	AKK68467.1 beta-galactosidase [Xanthomonas translucens pv. undulosa Xtu 4699]
15837442	NP_298130.1 beta-galactosidase [Xylella fastidiosa 9a5c]
386083781	YP_006000063.1 Beta-galactosidase [Xylella fastidiosa subsp. fastidiosa GB514]

170731075	YP_001776508.1 beta-galactosidase [Xylella fastidiosa M12]
182682446	YP_001830606.1 beta-galactosidase [Xylella fastidiosa M23]
28199702	NP_780016.1 beta-galactosidase [Xylella fastidiosa Temecula1]
Number of proteins = 58	
Gblocks 604/685 (88%)[a]	
**TOTAL: 420**	
[a]GBlock remove ambiguously aligned and uninformative positions in the resulting alignments.	

Table S3. Lipases found in *Xanthomonas* and the different functional parameters of each.

Old locus tag	New locus tag	OrthoMCL	Protein ID	PFAM domains	Size (aa)	TM domain	SP	Pathways	Name
XAC0501	XAC_RS02605	NP_640854.1 hypothetical protein	AAM35390.1	LIP; Hydrolase_4; Abhydrolase_6; Peptidase_S9; Peptidase_S15; DUF2974; Chlorophyllase2; DLH; Abhydrolase_4; Cutinase; Chlorophyllase	385	0	1-35	NA	NA
XAC2393	XAC_RS12170	NP_642709.1 carboxylestera se	AAM37245.1	Abhydrolase_3; Peptidase_S9; COesterase; Hydrolase_4; Este rase_phd; DLH; Abhydrolase_2; FSH1; Lipase_3	371	92-112	0	NA	NA
XAC3365	XAC_RS17050	NP_643672.1 hypothetical protein	AAM38208.1	PI-PLC-C1; PI-PLC-X	391	0	1-46	NA	NA
XAC1564	XAC_RS07955	NP_641897.1 acetylhydrolas	AAM36433.1	Lipase_GDSL_2; Lipase_GDS L; Lipase_GDSL_3	476	0	0	NA	NA

		e							
XAC3674	XAC_RS18585	NP_643981.1 hypothetical protein	AAM38517.1	Hydrolase_4; Esterase; UPF0227; Abhydrolase_1; BAAT_C; Abhydrolase_2; Peptidase_S9; Abhydrolase_3; DLH; Peptidase_S28	289	0	0	NA	NA
XAC0372	XAC_RS01965	NP_640728.1 hydrolase	AAM35264.1	Hydrolase_4; Abhydrolase_6; Abhydrolase_1; Peptidase_S15; Peptidase_S9; DLH; Abhydrolase_4; Thioesterase; BAAT_C; FSH1	280	0	0	NA	NA
XAC4310	XAC_RS21735	NP_644604.1 cardiolipin synthetase	AAM39140.1	PLDc_2; PLDc	407	244-265	0	xac00564	Glycerophospholipid metabolism
XAC2990	XAC_RS15180	NP_643299.1 hypothetical protein	AAM37835.1	Lipase_3	460	0	0	NA	NA

ACU13_12325	ACU13_12325	AKN93695.1 hypothetical protein	AKN93695.1	Unknown	201	0	0	Unknown	Unknown
XC_1740	XC_RS08720	YP_242825.1 hypothetical protein	AAY48805.1	LIP; Hydrolase_4; Abhydrolase_6; Peptidase_S9; Peptidase_S15; DLH	376	0	0	NA	NA
XCC0265	XCC0265	NP_635660.1 lipase	AAM39584.1	DUF732; Phage_int_SAM_1	91	0	1-19	NA	NA
SB85_02285	SB85_02285	AJC44764.1 phospholipase	AJC44764	PLDc_2; PLDc; PLDc_3; Regulator_TrmB; DUF1669	426	0	1-33	NA	NA
XAC3159	XAC3159	NP_643467.1 phospholipase C	AAM38003.1	Phosphoesterase; TAT_signal	201	0	1-29	NA	NA
PXO_01547	PXO_01547	YP_001914375.1 Non-hemolytic phospholipase C	ACD59843.1	Phosphoesterase; TAT_signal	220	0	1-28	NA	NA

*COG1075: Predicted acetyltransferases and hydrolases with the alpha/beta hydrolase fold.

Table S4. LipA canonical residues analysis in different genomes.

Strain	Accession	Model (Fig. 2)	Size	Topology analysis					Canonical residues		
				Template	Residues	Confidence	Coverage	Identity	S176	D336	H377
Xac306	WP_015462810	1	421	C3h2iA	382	100%	99%	87%	164	324	365
Xcv8510	WP_011346188	4	420	C3h2iA	387	100%	92%	87%	196	359	400
XalbGPEPC73	WP_012917605	5	421	C3h2iA	387	100%	92%	70%	200	360	401
Xyf9a5c-1	WP_010892890	6	387	C3h2iA	382	100%	99%	69%	165	325	367
Xyf9a5c-3	WP_010894600	8	424	C3h2iA	383	100%	90%	61%	204	362	404
Xfus4834	WP_022558105	2	420	C3h2iA	387	100%	92%	88%	199	359	400
XcamATCC33913	WP_011038080	3	382	C3h2iA	379	100%	99%	72%	163	321	362
Xyf-9a5c-2	AAF83168	7	385	C3h2iA	380	100%	99%	62%	165	323	365
Burkholderia graminis	WP_006047192	9	527	C3h2iA	398	100%	74%	38%	222	431	**A474**
Pseudoduganella violaceinigra	WP_051293673	10	513	C3h2iA	388	100%	76%	30%	228	411	**E449**
Acidovorax delafieldii	WP_005799851	11	435	C3h2iA	361	100%	83%	38%	195	355	**P399**

Table S5. Validation of the phytopathogen core genome using tblastn.

	Xanthomonas oryzae pv. oryzae MAFF 311018	Xanthomonas oryzae pv. oryzicola YM15	Xanthomonas translucens pv. undulosa Xtu 4699	Xanthomonas citri pv. citri jx-6	Xanthomonas oryzae pv. oryzicola CFBP2286	Xanthomonas oryzae pv. oryzicola B8-12	Xanthomonas oryzae pv. oryzicola BLS279	Xanthomonas oryzae pv. oryzicola BXOR1	Xanthomonas oryzae pv. oryzicola CFBP7331	Xanthomonas oryzae pv. oryzicola CFBP7341	Xanthomonas oryzae pv. oryzicola L8
gene	ASM1002v1	ASM102191v1	ASM102193v1	ASM102828v2	ASM104273v1	ASM104274v1	ASM104277v1	ASM104279v1	ASM104281v1	ASM104283v1	ASM104285v1
xac:XAC0435	239	237	123	255	236	236	236	237	237	237	236
xac:XAC0501	699	717	592	796	716	716	716	717	717	717	716
xac:XAC1306	1151	1146	1094	1176	1153	1151	1151	1153	1153	1153	1151
xac:XAC3072	1035	1038	894	1076	1036	1040	1040	1033	1038	1038	1040
xac:XAC3073	692	695	642	705	696	696	696	691	692	692	696
xac:XAC3084	1126	1132	911	1186	1133	1131	1131	1132	1132	1132	1131
xac:XAC3647	325	325	230	347	325	323	325	325	322	322	323
gene	ASM1002v1	ASM102191v1	ASM102193v1	ASM102828v2	ASM104273v1	ASM104274v1	ASM104277v1	ASM104279v1	ASM104281v1	ASM104283v1	ASM104285v1
xac:XAC0435	0,937254902	0,929411765	0,482352941	1	0,925490196	0,925490196	0,925490196	0,929411765	0,929411765	0,929411765	0,925490196
xac:XAC0501	0,878140704	0,900753769	0,743718593	1	0,899497487	0,899497487	0,899497487	0,900753769	0,900753769	0,900753769	0,899497487
xac:XAC1306	0,978741497	0,974489796	0,930272109	1	0,980442177	0,978741497	0,978741497	0,980442177	0,980442177	0,980442177	0,978741497
xac:XAC3072	0,961895911	0,964684015	0,830855019	1	0,962825279	0,966542751	0,966542751	0,960037175	0,964684015	0,964684015	0,966542751
xac:XAC3073	0,981560284	0,985815603	0,910638298	1	0,987234043	0,987234043	0,987234043	0,980141844	0,981560284	0,981560284	0,987234043
xac:XAC3084	0,949409781	0,954468803	0,768128162	1	0,955311973	0,953625632	0,953625632	0,954468803	0,954468803	0,954468803	0,953625632
xac:XAC3647	0,936599424	0,936599424	0,662824207	1	0,936599424	0,930835735	0,936599424	0,936599424	0,92795389	0,92795389	0,930835735
	Xanthomonas oryzae pv. oryzae MAFF 311018	Xanthomonas oryzae pv. oryzicola YM15	Xanthomonas translucens pv. undulosa Xtu 4699	Xanthomonas citri pv. citri jx-6	Xanthomonas oryzae pv. oryzicola CFBP2286	Xanthomonas oryzae pv. oryzicola B8-12	Xanthomonas oryzae pv. oryzicola BLS279	Xanthomonas oryzae pv. oryzicola BXOR1	Xanthomonas oryzae pv. oryzicola CFBP7331	Xanthomonas oryzae pv. oryzicola CFBP7341	Xanthomonas oryzae pv. oryzicola L8

Xanthomonas oryzae pv. oryzicola RS105	Xanthomonas campestris pv. campestris ICMP 21080	Xanthomonas campestris pv. campestris ICMP 4013	Xanthomonas campestris pv. campestris str. 8004	Stenotrophomona s maltophilia ISMMS3	Stenotrophomona s maltophilia ISMMS2	Stenotrophomona s maltophilia ISMMS2R	Stenotrophomona s acidaminiphila ZAC14D2_NAI MI4_2	Xylella fastidiosa subsp. fastidiosa GB514	Xanthomonas oryzae pv. oryzicola BLS256	Pseudoxanthomo nas suwonensis 11-1	Xylella fastidiosa M12
ASM104287v1	ASM118641 v1	ASM118646v 1	ASM1210v1	ASM127459v 1	ASM127465 v1	ASM127467v 1	ASM131430v 1	ASM14840v1	ASM16831v3	ASM18596v1	ASM1932 v1
236	218	219	219	0	0	0	0	103	236	0	105
716	583	583	583	0	0	0	0	561	716	0	563
1151	1138	1138	1138	0	0	0	0	237	1153	0	239
1040	1010	1011	1011	0	0	0	0	822	1040	0	827
696	669	669	671	0	0	0	0	528	696	0	525
1131	1083	1088	1083	0	0	0	0	852	1133	0	846
323	272	266	266	0	0	0	0	203	325	0	199
ASM104287v1	ASM118641 v1	ASM118646v 1	ASM1210v1	ASM127459v 1	ASM127465 v1	ASM127467v 1	ASM131430v 1	ASM14840v1	ASM16831v3	ASM18596v1	ASM1932 v1
0,925490196	0,854901961	0,858823529	0,858823529	0	0	0	0	0,403921569	0,925490196	0	0,411764706
0,899497487	0,73241206	0,73241206	0,73241206	0	0	0	0	0,704773869	0,899497487	0	0,707286432
0,978741497	0,967687075	0,967687075	0,967687075	0	0	0	0	0,201530612	0,980442177	0	0,203231293
0,966542751	0,93866171	0,939591078	0,939591078	0	0	0	0	0,76394052	0,966542751	0	0,768587361
0,987234043	0,94893617	0,94893617	0,95177305	0	0	0	0	0,74893617	0,987234043	0	0,744680851
0,953625632	0,913153457	0,917369309	0,913153457	0	0	0	0	0,718381113	0,955311973	0	0,713322091
0,930835735	0,783861671	0,766570605	0,766570605	0	0	0	0	0,585014409	0,936599424	0	0,573487032
Xanthomonas oryzae pv. oryzicola RS105	Xanthomonas campestris pv. campestris ICMP 21080	Xanthomonas campestris pv. campestris ICMP 4013	Xanthomonas campestris pv. campestris str. 8004	Stenotrophomonas maltophilia ISMMS3	Stenotrophomonas maltophilia ISMMS2	Stenotrophomonas maltophilia ISMMS2R	Stenotrophomonas acidaminiphila ZAC14D2_NAIMI4 _2	Xylella fastidiosa subsp. fastidiosa GB514	Xanthomonas oryzae pv. oryzicola BLS256	Pseudoxanthomonas suwonensis 11-1	Xylella fastidiosa M12

Xanthomonas oryzae pv. oryzae PXO99A	Xylella fastidiosa M23	Stenotrophomonas maltophilia R551-3	Xanthomonas campestris pv. raphani 756C	Stenotrophomonas maltophilia JV3	Xanthomonas axonopodis pv. citrumelo F1	Pseudoxanthomonas spadix BD-a59	Stenotrophomonas maltophilia D457	Xanthomonas axonopodis Xac29-1	Xanthomonas citri subsp. citri Aw12879	Xylella fastidiosa 9a5c	Xylella fastidiosa subsp. sandyi Ann-1
ASM1958v2	ASM1976v1	ASM2066v1	ASM22196v1	ASM22388v1	ASM22591v1	ASM23391v4	ASM28459v1	ASM34858v1	ASM34922v1	ASM672v1	ASM69880v1
239	103	0	216	0	253	0	0	255	255	108	103
699	568	0	578	0	768	0	0	796	796	575	582
1150	237	0	1127	0	1157	0	0	1176	1172	238	238
1037	822	0	1012	0	1051	0	0	1076	1073	519	823
696	528	0	668	0	699	0	0	705	705	532	535
1126	852	0	1079	0	1140	0	0	1186	1186	856	844
325	203	0	271	0	337	0	0	347	347	204	201
ASM1958v2	ASM1976v1	ASM2066v1	ASM22196v1	ASM22388v1	ASM22591v1	ASM23391v4	ASM28459v1	ASM34858v1	ASM34922v1	ASM672v1	ASM69880v1
0,937254902	0,403921569	0	0,847058824	0	0,992156863	0	0	1	1	0,423529412	0,403921569
0,878140704	0,713567839	0	0,726130653	0	0,964824121	0	0	1	1	0,722361809	0,731155779
0,977891156	0,201530612	0	0,958333333	0	0,983843537	0	0	1	0,996598639	0,202380952	0,202380952
0,963754647	0,76394052	0	0,940520446	0	0,976765799	0	0	1	0,997211896	0,482342007	0,764869888
0,987234043	0,74893617	0	0,94751773	0	0,991489362	0	0	1	1	0,754609929	0,758865248
0,949409781	0,718381113	0	0,909780776	0	0,961214165	0	0	1	1	0,721753794	0,71163575
0,936599424	0,585014409	0	0,780979827	0	0,971181556	0	0	1	1	0,587896254	0,57925072
Xanthomonas oryzae pv. oryzae PXO99A	Xylella fastidiosa M23	Stenotrophomonas maltophilia R551-3	Xanthomonas campestris pv. raphani 756C	Stenotrophomonas maltophilia JV3	Xanthomonas axonopodis pv. citrumelo F1	Pseudoxanthomonas spadix BD-a59	Stenotrophomonas maltophilia D457	Xanthomonas axonopodis Xac29-1	Xanthomonas citri subsp. citri Aw12879	Xylella fastidiosa 9a5c	Xylella fastidiosa subsp. sandyi Ann-1

Xylella fastidiosa MUL0034	Xanthomonas campestris pv. campestris B100	Xanthomonas campestris pv. campestris str. ATCC 33913	Xanthomonas axonopodis pv. citri str. 306	Stenotrophomonas maltophilia K279a	Xylella fastidiosa Temecula1	Xanthomonas oryzae pv. oryzae KACC 10331	Xanthomonas sacchari R1	Xanthomonas citri subsp. citri A306	Xanthomonas albilineans GPE PC73	Xanthomonas campestris pv. vesicatoria str. 85-10	Xanthomonas oryzae pv. oryzicola CFBP7342
ASM69882v1	ASM7060v1	ASM714v1	ASM716 v1	ASM7248v1	ASM724v1	ASM738v1	ASM81518v1	ASM81688v1	ASM8796v1	ASM916v1	ASM94082v1
103	219	219	255	0	103	239	137	255	134	255	237
575	583	583	796	0	568	699	580	796	561	763	717
238	1138	1138	1176	0	237	1151	1109	1176	235	1154	1153
821	1011	1011	1076	0	822	1037	905	1076	877	1031	1035
525	671	671	705	0	528	696	632	705	625	682	695
854	1088	1085	1186	0	852	1126	940	1186	890	1144	1132
200	272	266	347	0	203	321	251	347	238	336	325
ASM69882v1	ASM7060v1	ASM714v1	ASM716 v1	ASM7248v1	ASM724v1	ASM738v1	ASM81518v1	ASM81688v1	ASM8796v1	ASM916v1	ASM94082v1
0,403921569	0,858823529	0,858823529	1	0	0,403921569	0,937254902	0,537254902	1	0,525490196	1	0,929411765
0,722361809	0,73241206	0,73241206	1	0	0,713567839	0,878140704	0,728643216	1	0,704773869	0,958542714	0,900753769
0,202380952	0,967687075	0,967687075	1	0	0,201530612	0,978741497	0,943027211	1	0,199829932	0,981292517	0,980442177
0,763011152	0,939591078	0,939591078	1	0	0,76394052	0,963754647	0,841078067	1	0,815055762	0,958178439	0,961895911
0,744680851	0,95177305	0,95177305	1	0	0,74893617	0,987234043	0,896453901	1	0,886524823	0,967375887	0,985815603
0,720067454	0,917369309	0,914839798	1	0	0,718381113	0,949409781	0,792580101	1	0,750421585	0,964586847	0,954468803
0,576368876	0,783861671	0,766570605	1	0	0,585014409	0,925072046	0,723342939	1	0,685878963	0,968299712	0,936599424
Xylella fastidiosa MUL0034	Xanthomonas campestris pv. campestris B100	Xanthomonas campestris pv. campestris str. ATCC 33913	Xanthomonas axonopodis pv. citri str. 306	Stenotrophomonas maltophilia K279a	Xylella fastidiosa Temecula1	Xanthomonas oryzae pv. oryzae KACC 10331	Xanthomonas sacchari R1	Xanthomonas citri subsp. citri A306	Xanthomonas albilineans GPE PC73	Xanthomonas campestris pv. vesicatoria str. 85-10	Xanthomonas oryzae pv. oryzicola CFBP7342

Xanthomonas oryzae pv. oryzae PXO86	Xanthomonas citri subsp. citri UI7	Xanthomonas citri subsp. citri UI6	Xanthomonas citri subsp. citri NT17	Xanthomonas citri subsp. citri MN12	Xanthomonas citri subsp. citri MN11	Xanthomonas citri subsp. citri MN10	Xanthomonas citri subsp. citri mf20	Xanthomonas citri subsp. citri jx5	Xanthomonas citri subsp. citri jx4	Xanthomonas citri subsp. citri gd3	Xanthomonas citri subsp. citri gd2
ASM94807v1	ASM96115v1	ASM96117v1	ASM96119v1	ASM96121v1	ASM96123v1	ASM96125v1	ASM96127v1	ASM96129v1	ASM96131v1	ASM96133v1	ASM96135v1
239	255	255	255	255	255	255	255	255	255	255	255
699	796	796	796	796	796	796	796	796	796	795	795
1151	1176	1176	1176	1176	1176	1176	1176	1176	1176	1176	1176
1035	1076	1076	1076	1076	1076	1076	1076	1076	1076	1076	1076
696	705	705	705	705	705	705	705	705	705	705	705
1126	1186	1186	1186	1186	1186	1186	1186	1186	1186	1186	1186
321	347	347	347	347	347	347	347	347	347	347	347
ASM94807v1	ASM96115v1	ASM96117v1	ASM96119v1	ASM96121v1	ASM96123v1	ASM96125v1	ASM96127v1	ASM96129v1	ASM96131v1	ASM96133v1	ASM96135v1
0,937254902	1	1	1	1	1	1	1	1	1	1	1
0,878140704	1	1	1	1	1	1	1	1	1	0,998743719	0,998743719
0,978741497	1	1	1	1	1	1	1	1	1	1	1
0,961895911	1	1	1	1	1	1	1	1	1	1	1
0,987234043	1	1	1	1	1	1	1	1	1	1	1
0,949409781	1	1	1	1	1	1	1	1	1	1	1
0,925072046	1	1	1	1	1	1	1	1	1	1	1
Xanthomonas oryzae pv. oryzae PXO86	Xanthomonas citri subsp. citri UI7	Xanthomonas citri subsp. citri UI6	Xanthomonas citri subsp. citri NT17	Xanthomonas citri subsp. citri MN12	Xanthomonas citri subsp. citri MN11	Xanthomonas citri subsp. citri MN10	Xanthomonas citri subsp. citri mf20	Xanthomonas citri subsp. citri jx5	Xanthomonas citri subsp. citri jx4	Xanthomonas citri subsp. citri gd3	Xanthomonas citri subsp. citri gd2

Xanthomonas citri subsp. citri FB19	Xanthomonas citri subsp. citri BL18	Xanthomonas citri subsp. citri 5208	Xanthomonas citri subsp. citri AW13	Xanthomonas citri subsp. citri AW14	Xanthomonas citri subsp. citri AW15	Xanthomonas citri subsp. citri AW16	Xanthomonas fuscans subsp. fuscans 4834-R	Xanthomonas campestris 17	Pseudoxanthomonas suwonensis J1
ASM96137v1	ASM96139v1	ASM96141v1	ASM96143v1	ASM96145v1	ASM96147v1	ASM96149v1	ASM96968v1	ASM97274v1	ASM97286v1
255	255	255	255	255	255	255	253	222	0
796	796	796	796	796	796	796	784	706	0
1176	1176	1176	1175	1175	1175	1175	1154	1153	0
1076	1076	1076	1073	1073	1073	1073	1053	1004	0
705	705	705	705	705	705	705	701	682	0
1186	1186	1186	1186	1186	1186	1186	1165	1086	0
347	347	347	347	347	347	347	339	260	0
ASM96137v1	ASM96139v1	ASM96141v1	ASM96143v1	ASM96145v1	ASM96147v1	ASM96149v1	ASM96968v1	ASM97274v1	ASM97286v1
1	1	1	1	1	1	1	0,992156863	0,870588235	0
1	1	1	1	1	1	1	0,984924623	0,886934673	0
1	1	1	0,99914966	0,99914966	0,99914966	0,99914966	0,981292517	0,980442177	0
1	1	1	0,997211896	0,997211896	0,997211896	0,997211896	0,978624535	0,933085502	0
1	1	1	1	1	1	1	0,994326241	0,967375887	0
1	1	1	1	1	1	1	0,982293423	0,915682968	0
1	1	1	1	1	1	1	0,976945245	0,749279539	0
Xanthomonas citri subsp. citri FB19	Xanthomonas citri subsp. citri BL18	Xanthomonas citri subsp. citri 5208	Xanthomonas citri subsp. citri AW13	Xanthomonas citri subsp. citri AW14	Xanthomonas citri subsp. citri AW15	Xanthomonas citri subsp. citri AW16	Xanthomonas fuscans subsp. fuscans 4834-R	Xanthomonas campestris 17	Pseudoxanthomonas suwonensis J1

Supplementary Figures

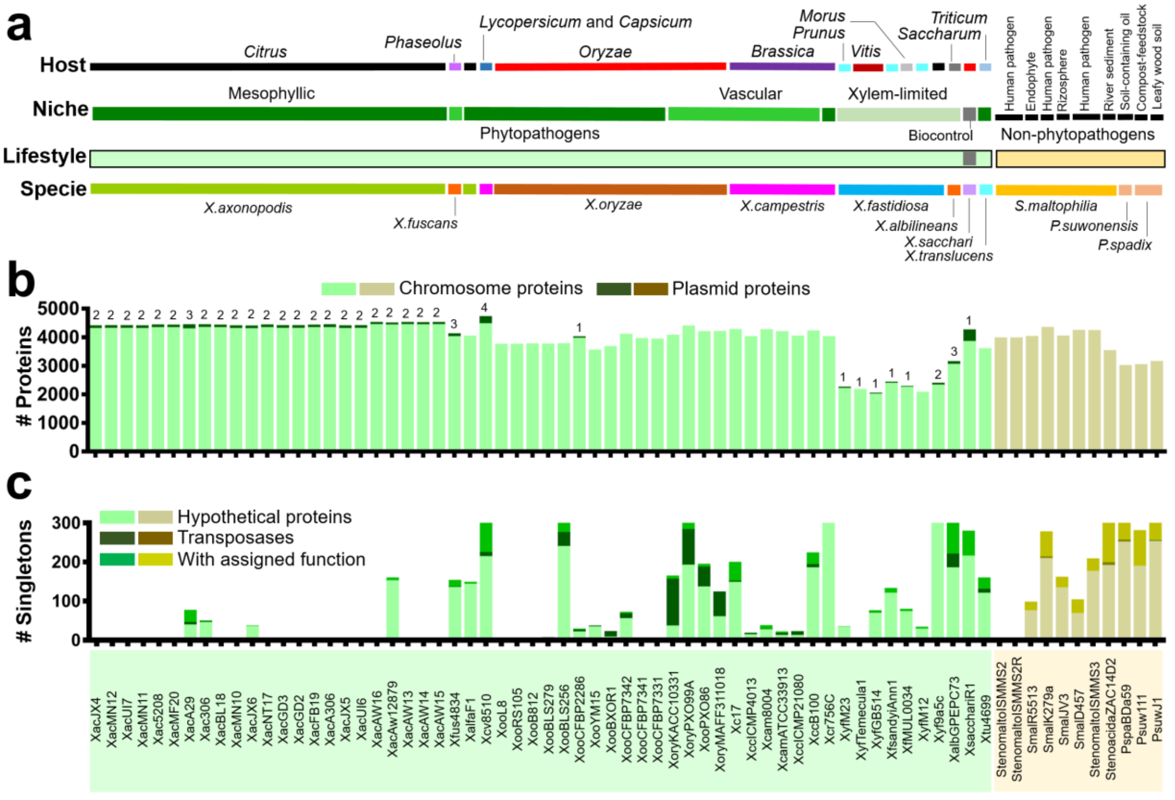

Fig. S1. Comparative genomics of the 69 selected strains. (a) General information about each strain classified into phytopathogens (green) and non-phytopathogens (brown). (b) Number of chromosome and plasmid proteins of respective strain. Numbers above the bars indicate number of plasmids in each strain. (c) Number of singletons per genome classified into three annotation categories.

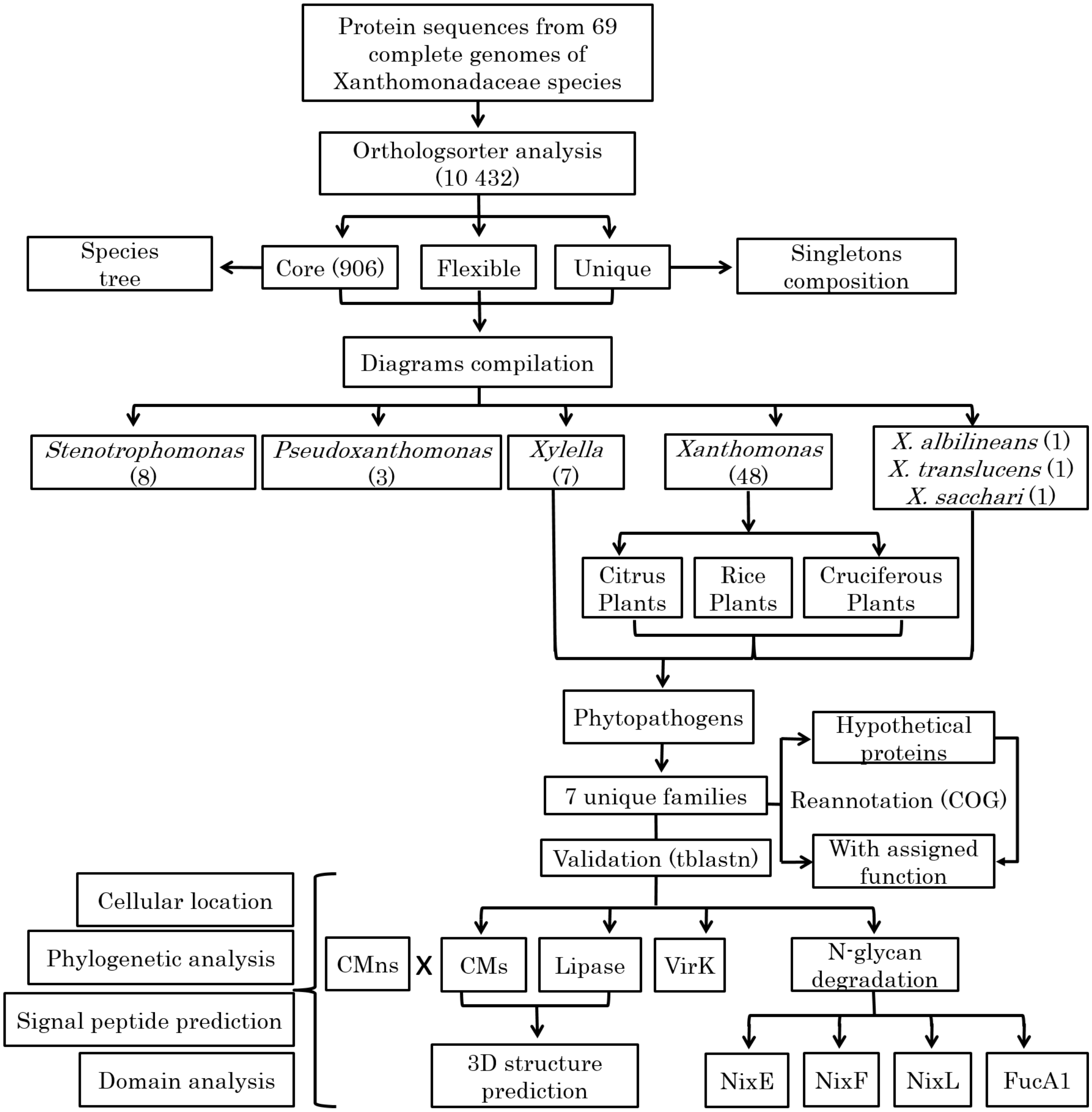

Fig. S2. Flowchart of analytical steps involved in this work.

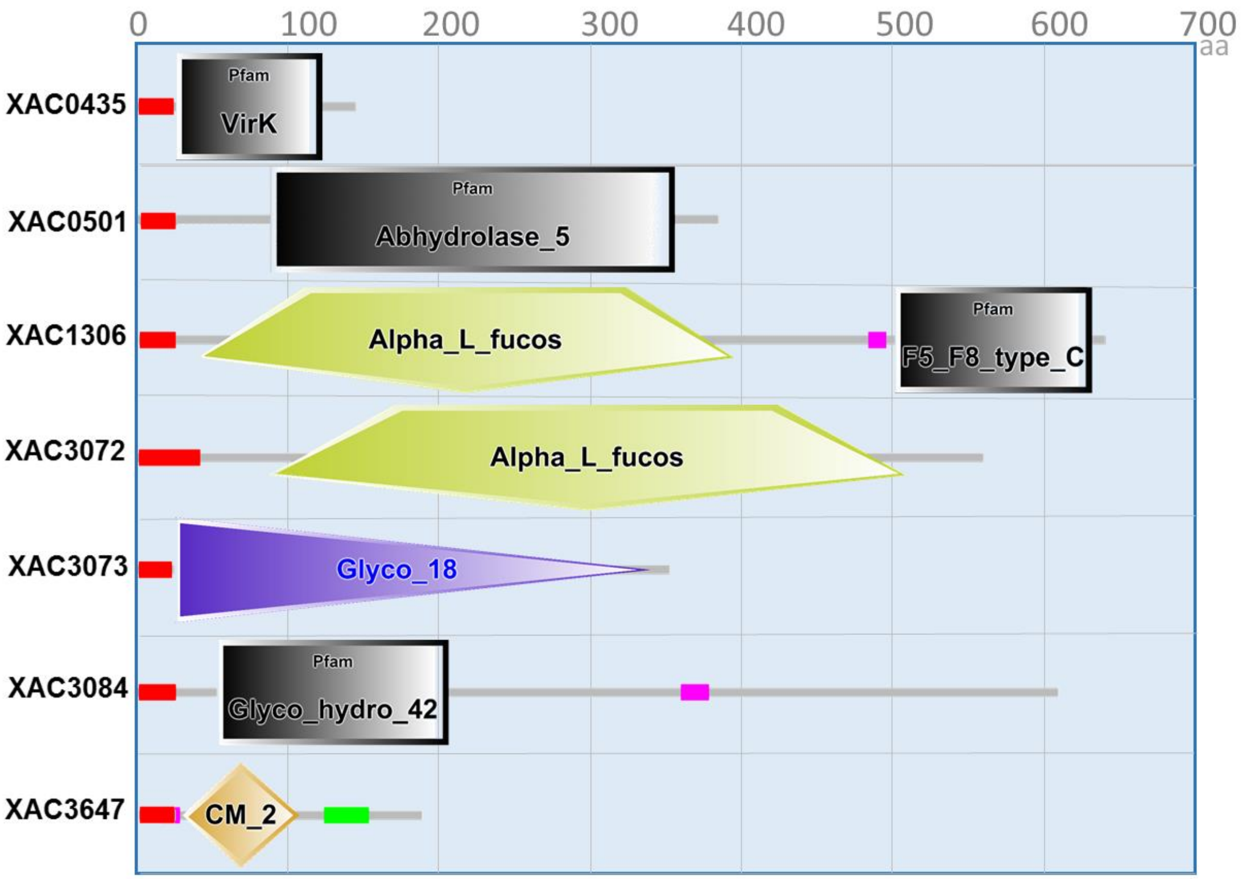

Fig. S3. Domain composition analysis of the seven proteins families using Xac306 as reference.

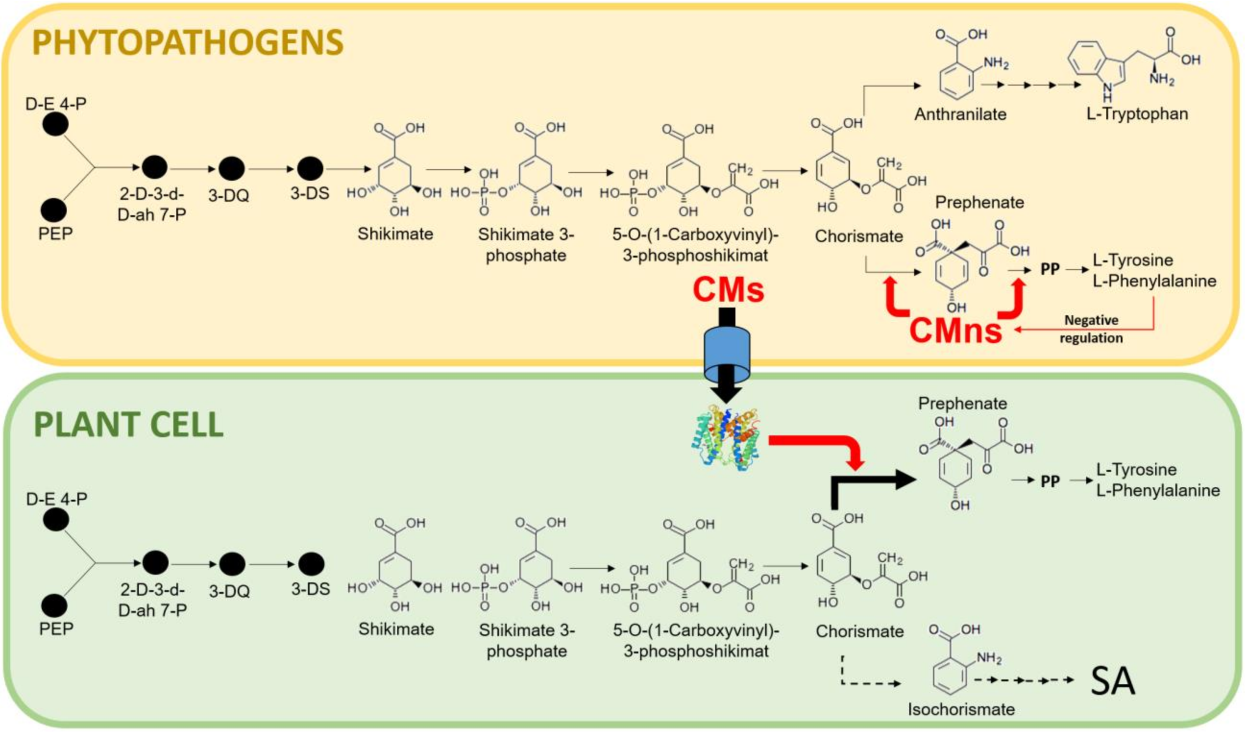

Fig. S4. Shikimate pathway highlighting the possible reactions catalyzed by *CM-sec* and *CM-nonsec* in the metabolism of plants and phytopathogens.

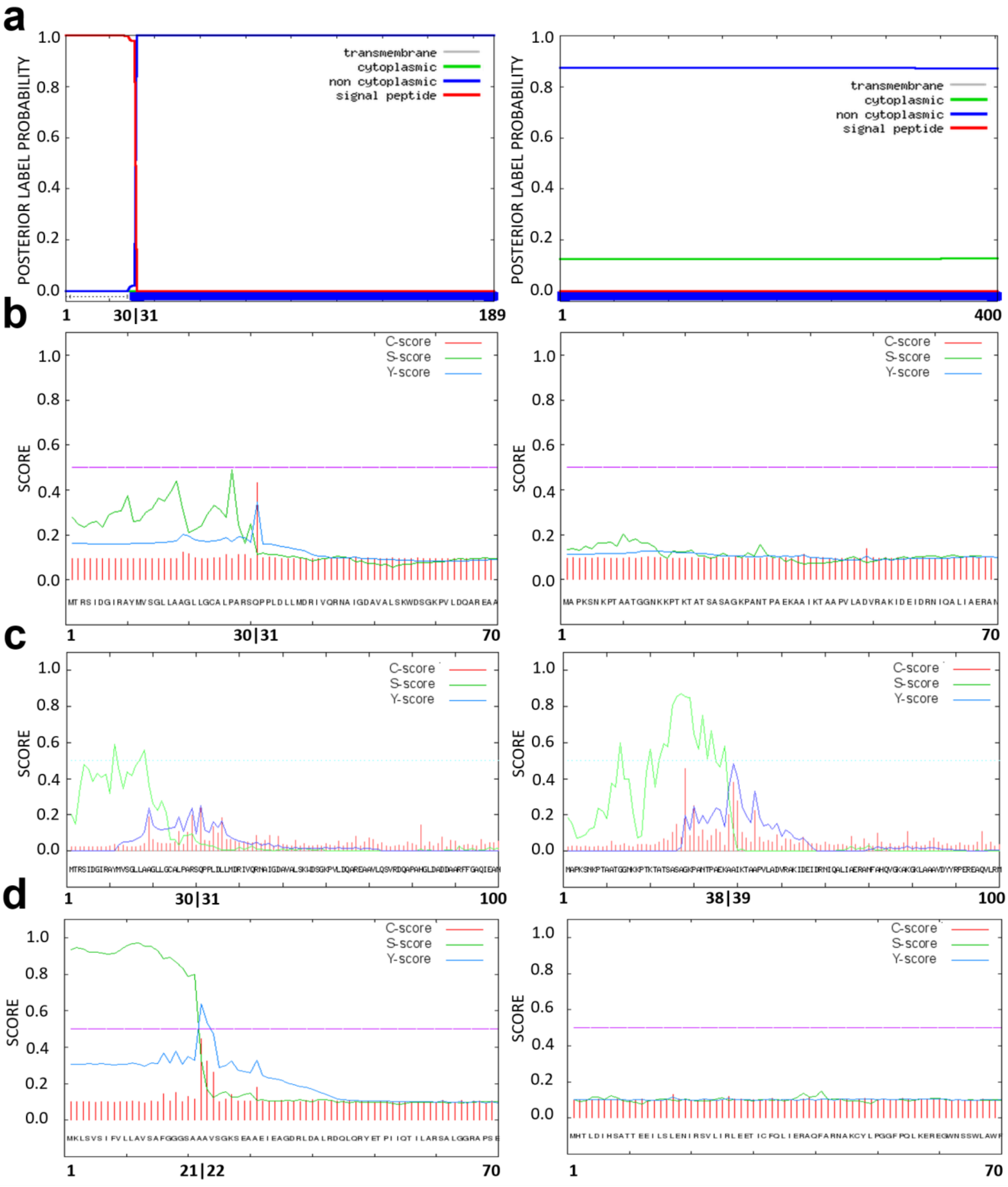

Fig. S5. Signal peptide prediction of CM-sec and CM-nonsec.

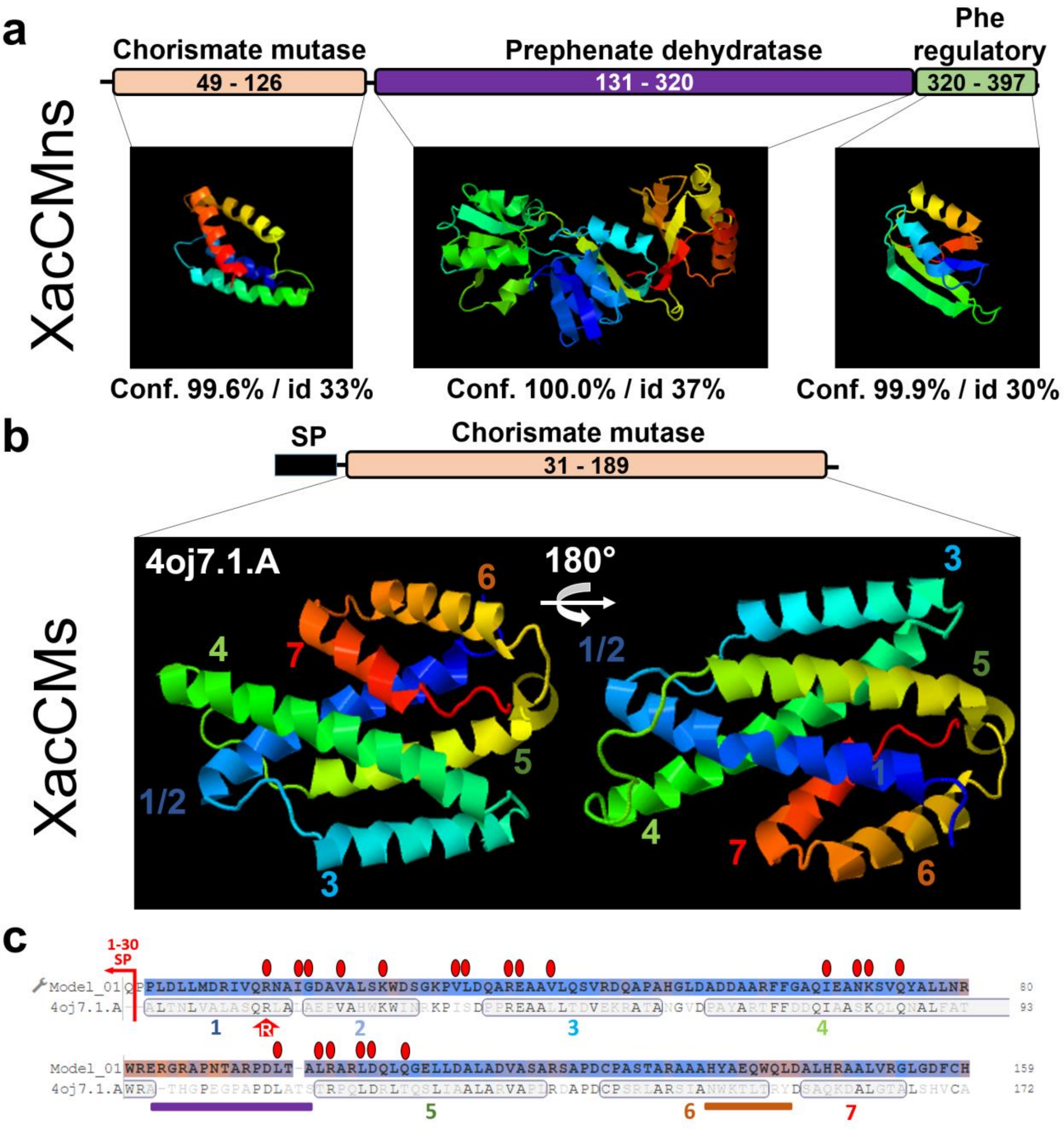

Model	Template	Hit	Seq Identity	Coverage	Description
XAC3647	4oj7.1.A	*Burkholderia thailandensis*	**39.74%**	**98.2%**	**Chorismate mutase**

Fig. S6. Structural analysis of chorismate mutase.

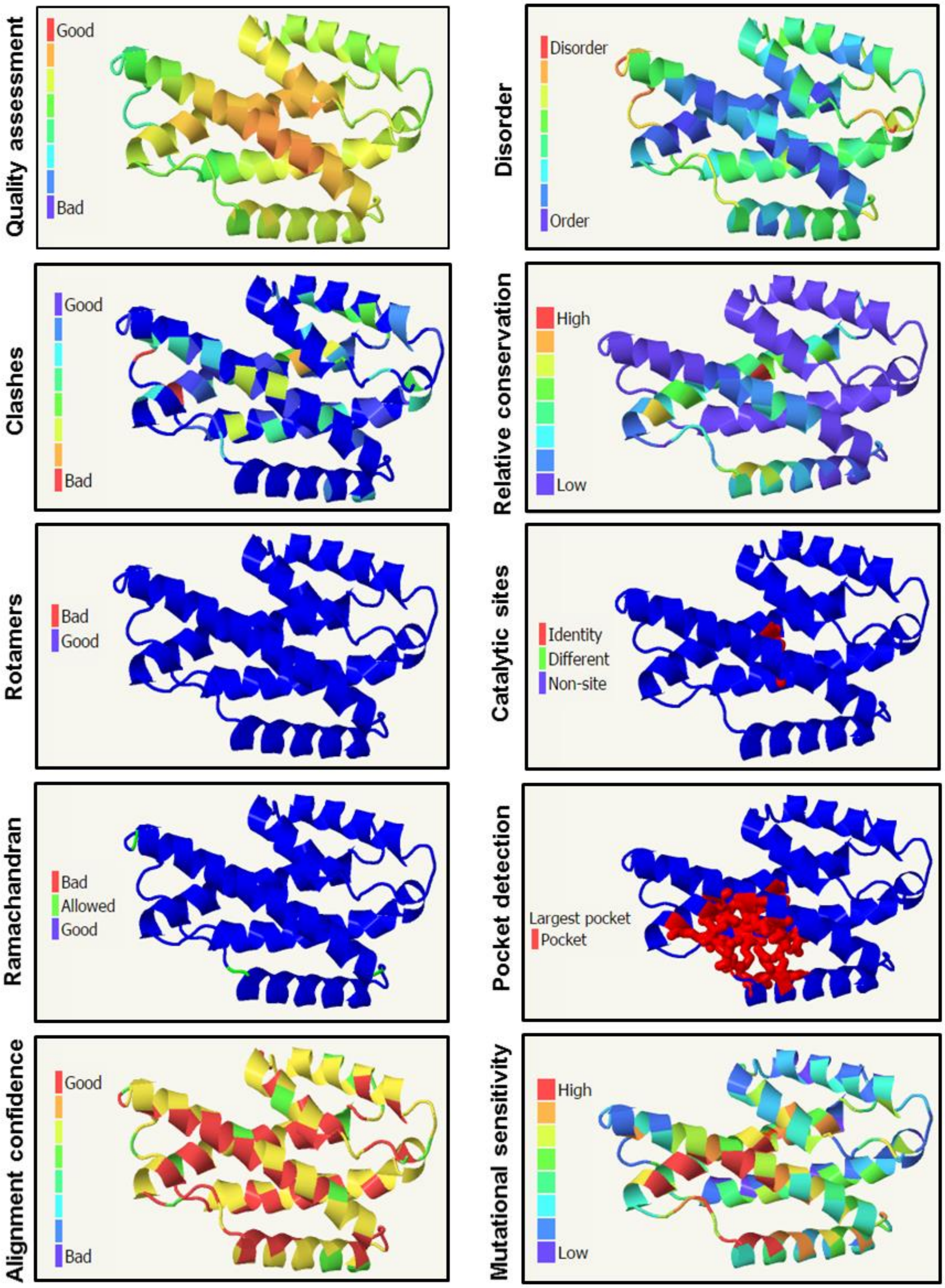

Fig. S7. Structural parameters analysis that denotes quality of *CM-sec* protein topology.

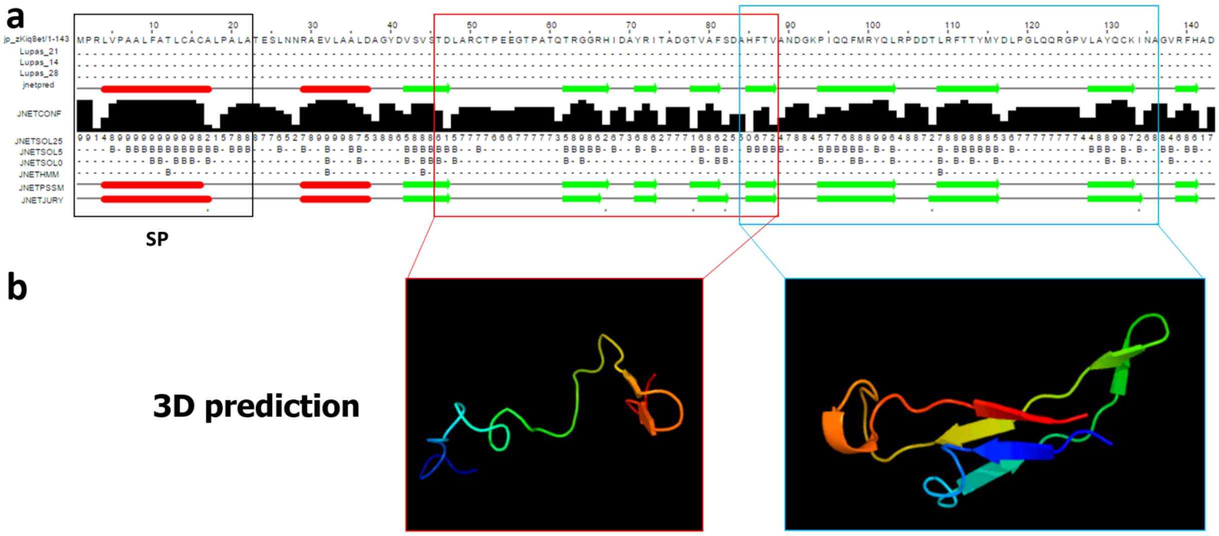

Template Information

Model	c4o01D
PDB header	Transferase
PDB Molecule	Bacteriophytochrome
Residues	37
Confidence	54.9%
Identity	19
Coverage	26%

Model	d1eapb2
PDB header	Immunoglobulin
PDB Molecule	Immunoglobulin-like
Residues	51
Confidence	41.9%
Identity	10
Coverage	36%

Fig. S8. VirK structural analysis.

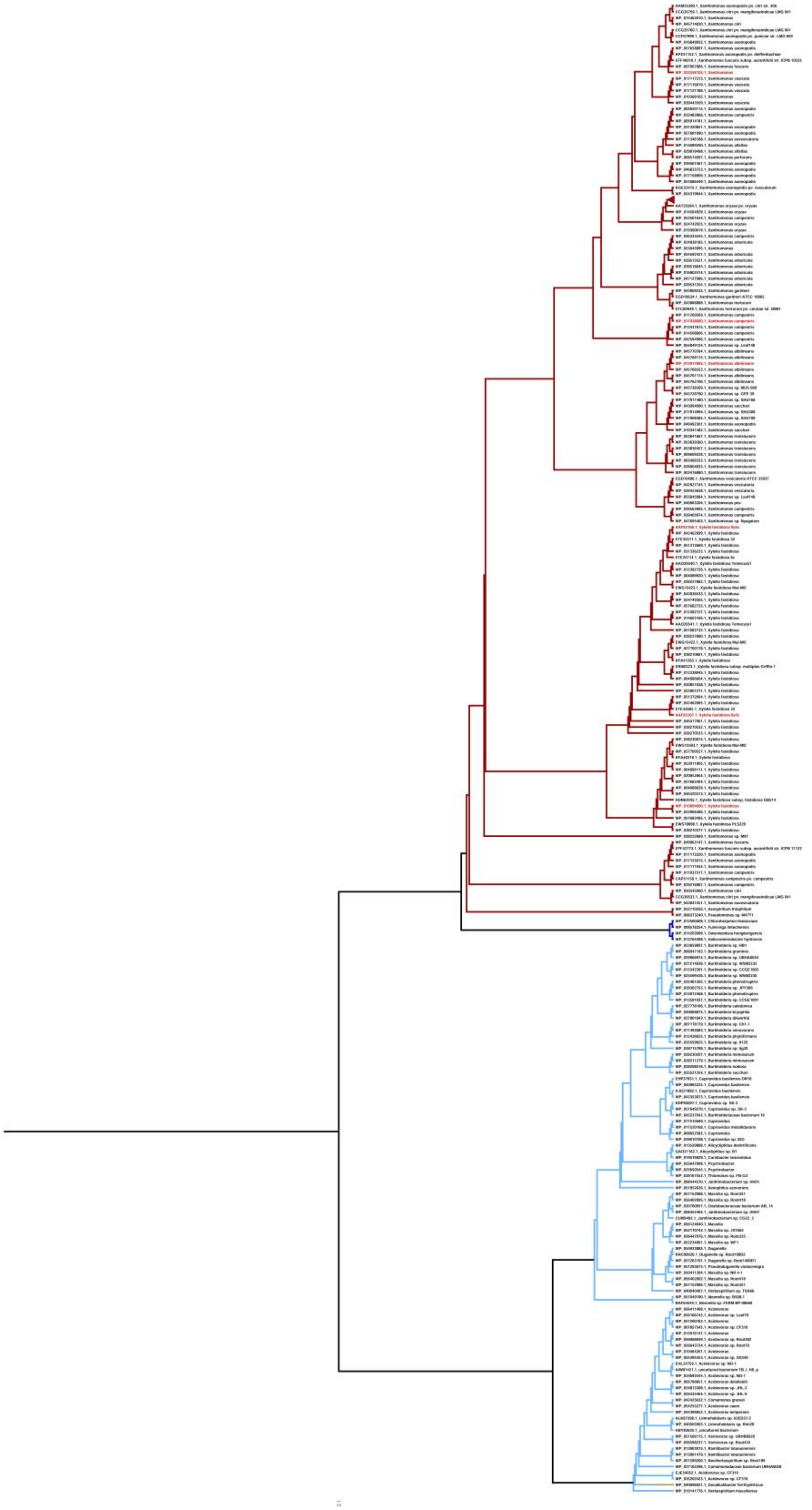

Fig. S9. Maximum likelihood phylogenetic tree of LipA.

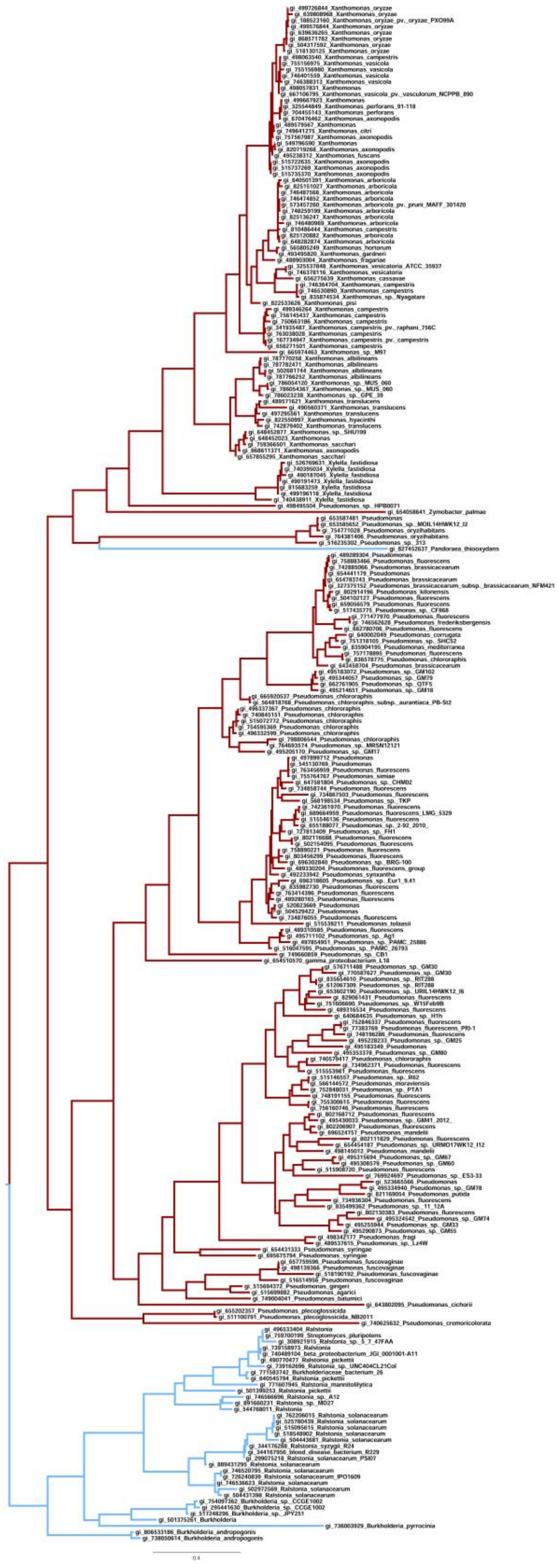

Fig. S10. Maximum likelihood phylogenetic tree of *CM-sec*.

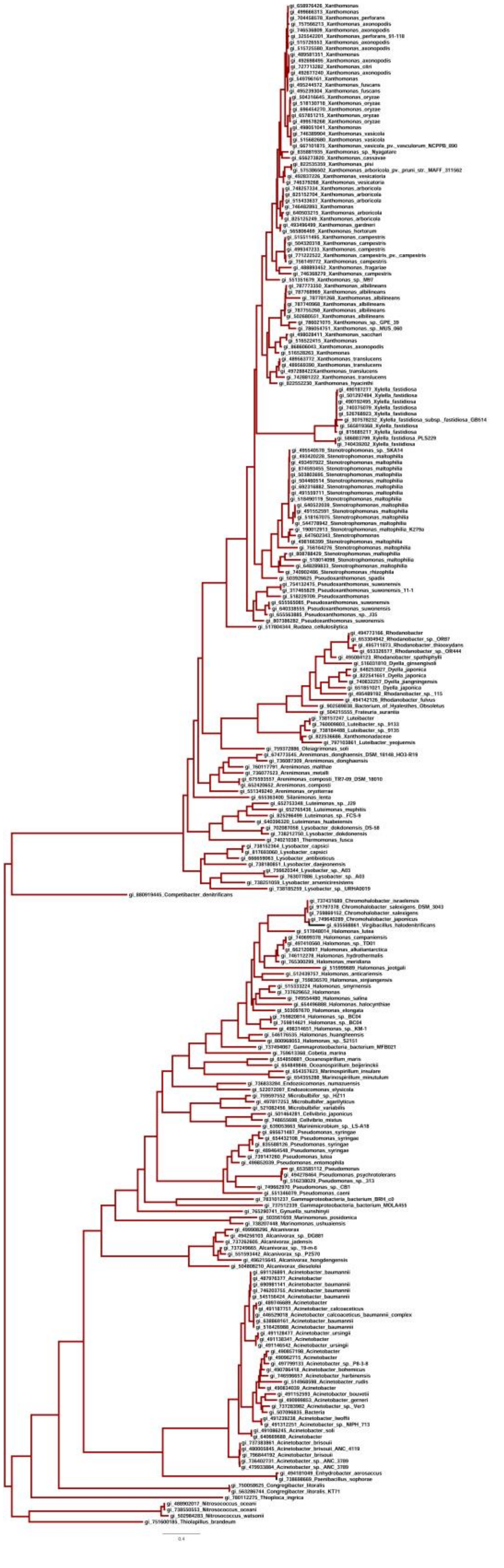

Fig. S11. Maximum likelihood phylogenetic tree of CM-nonsec.

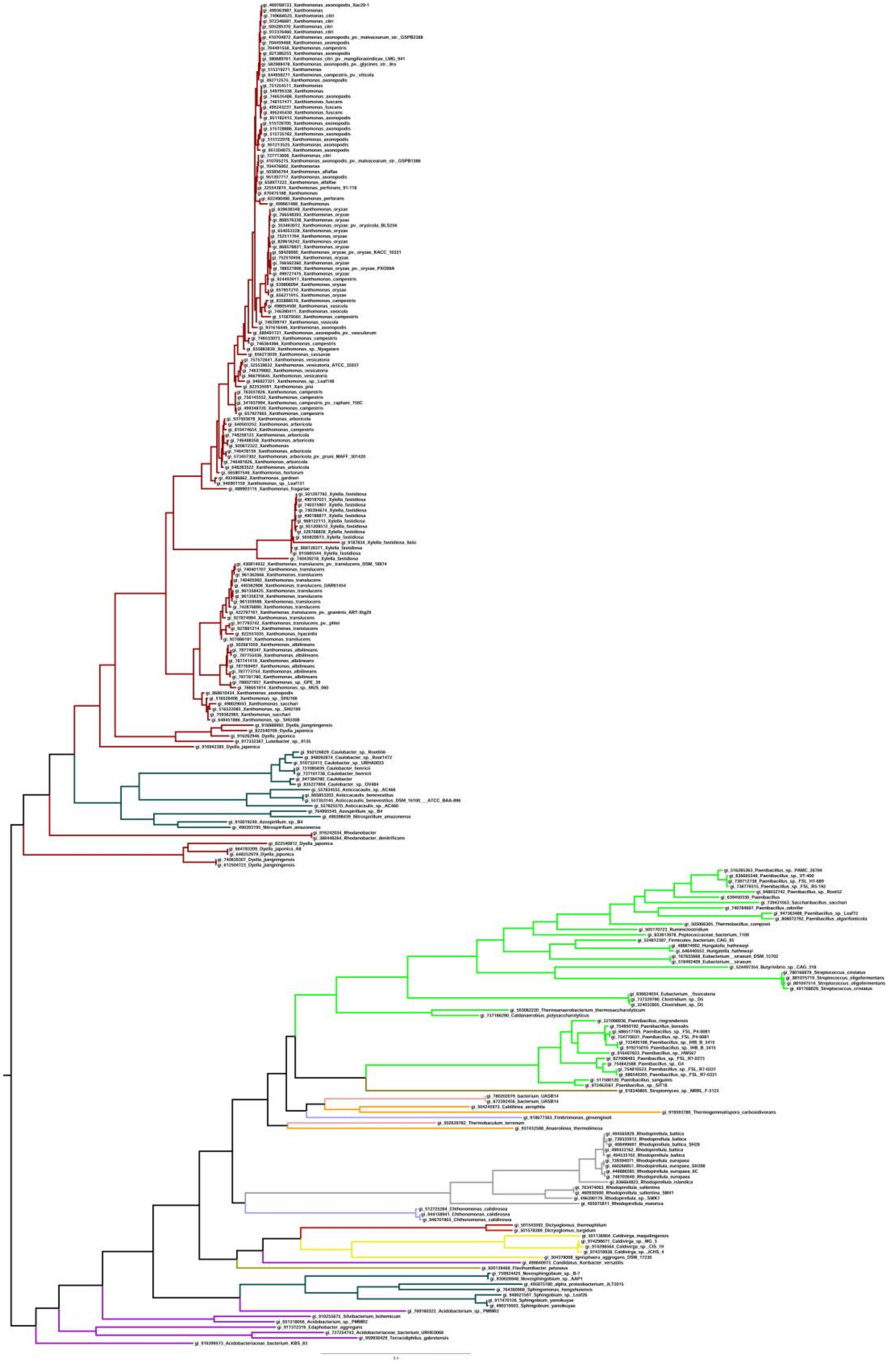

Fig. S12. Maximum likelihood phylogenetic tree of NixE.

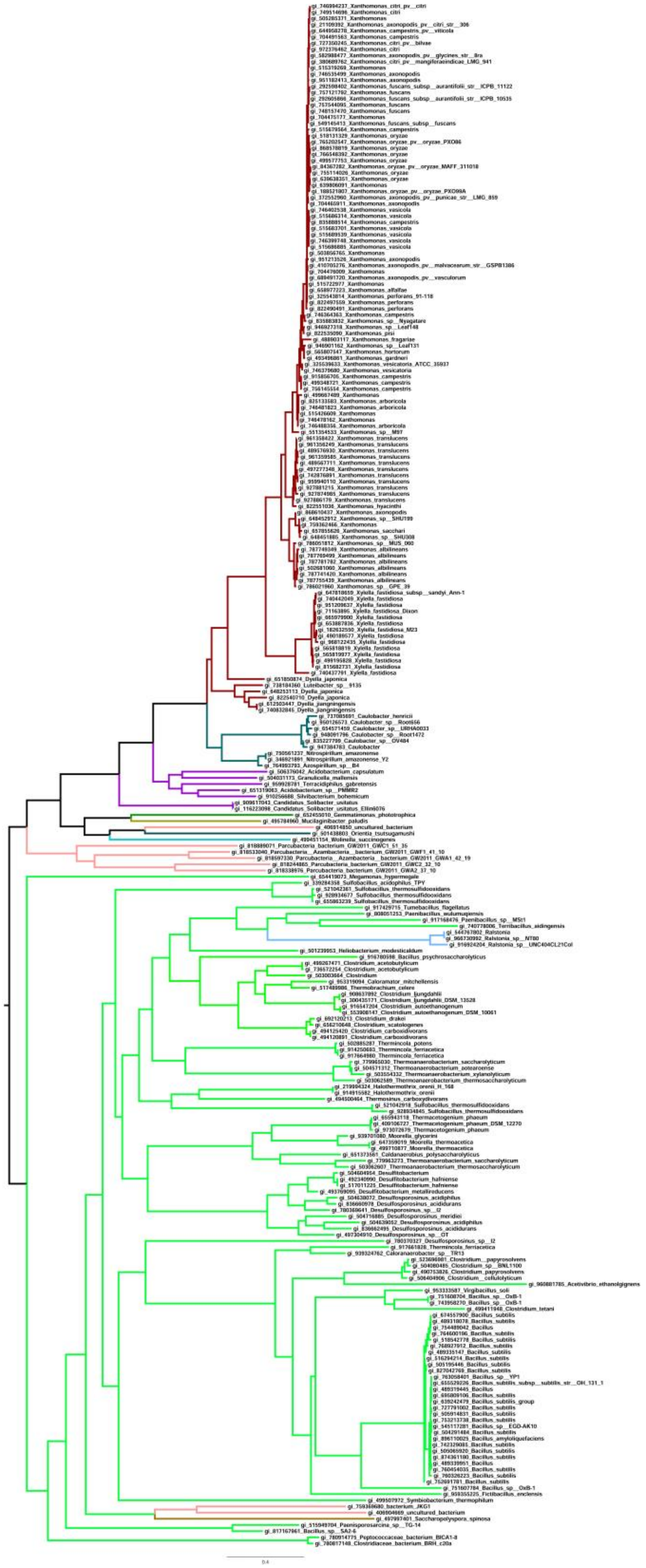

Fig. S13. Maximum likelihood phylogenetic tree of NixF.

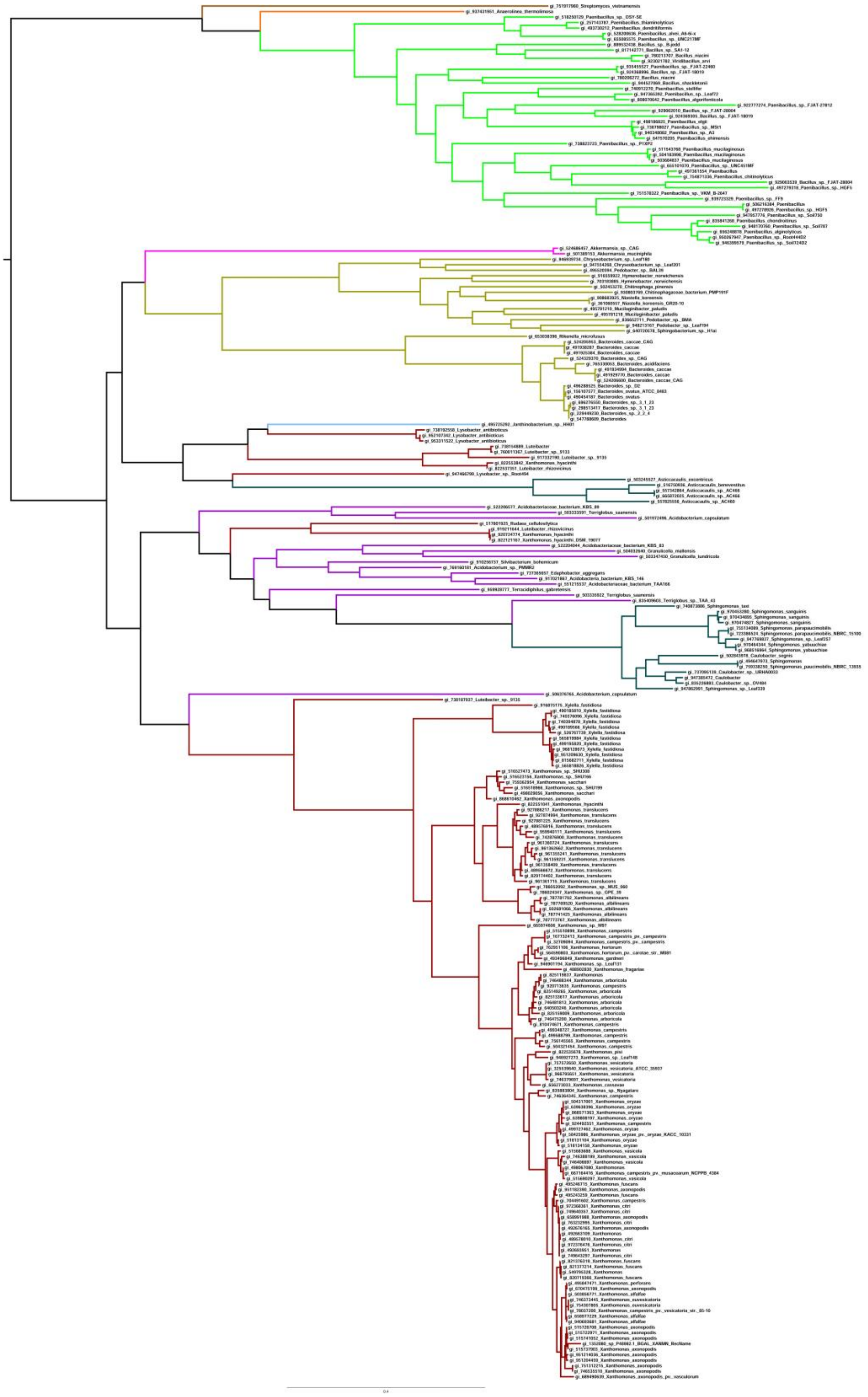

Fig. S14. Maximum likelihood phylogenetic tree of NixL.

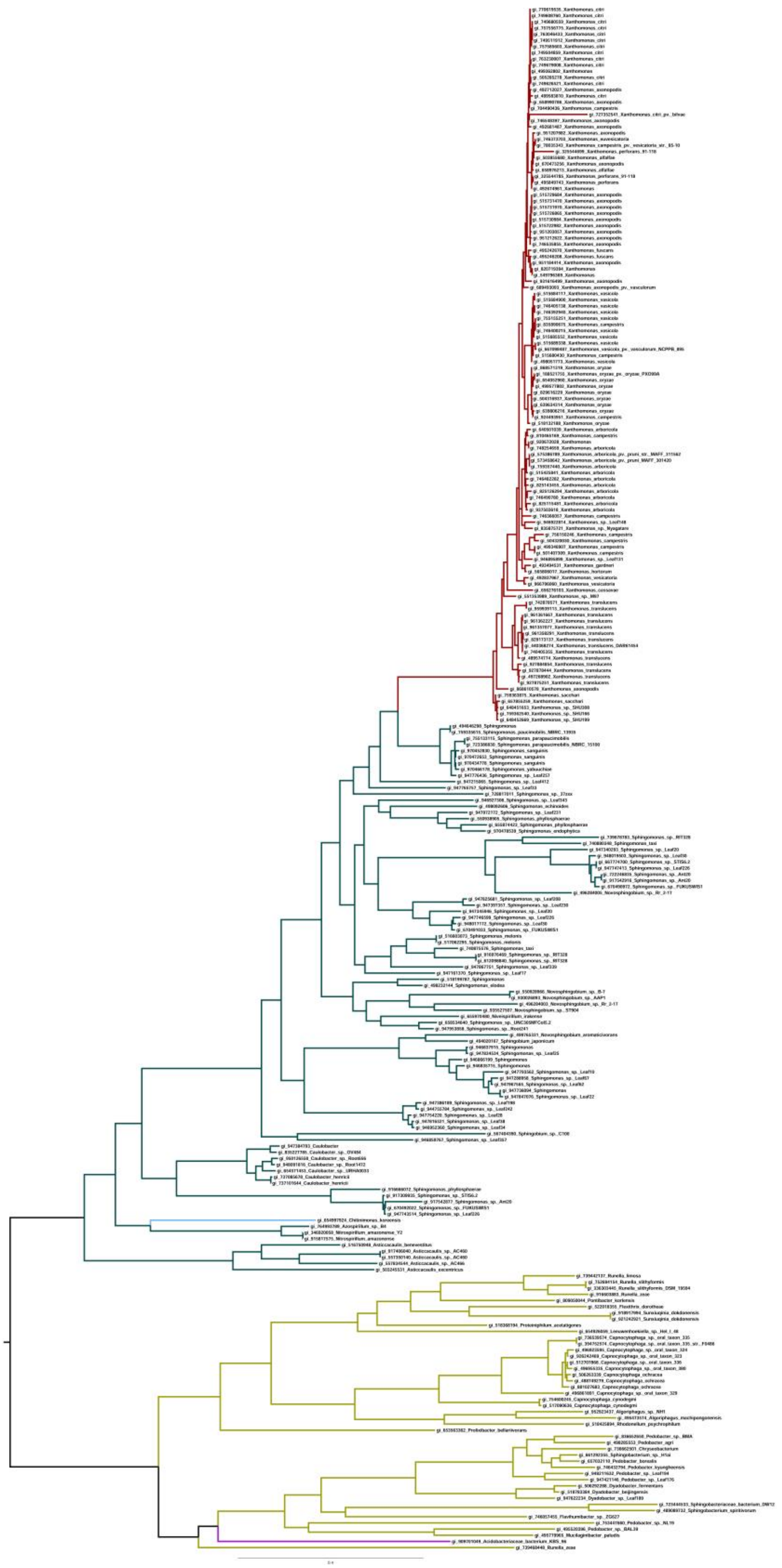

Fig. S15. Maximum likelihood phylogenetic tree of FucA1.

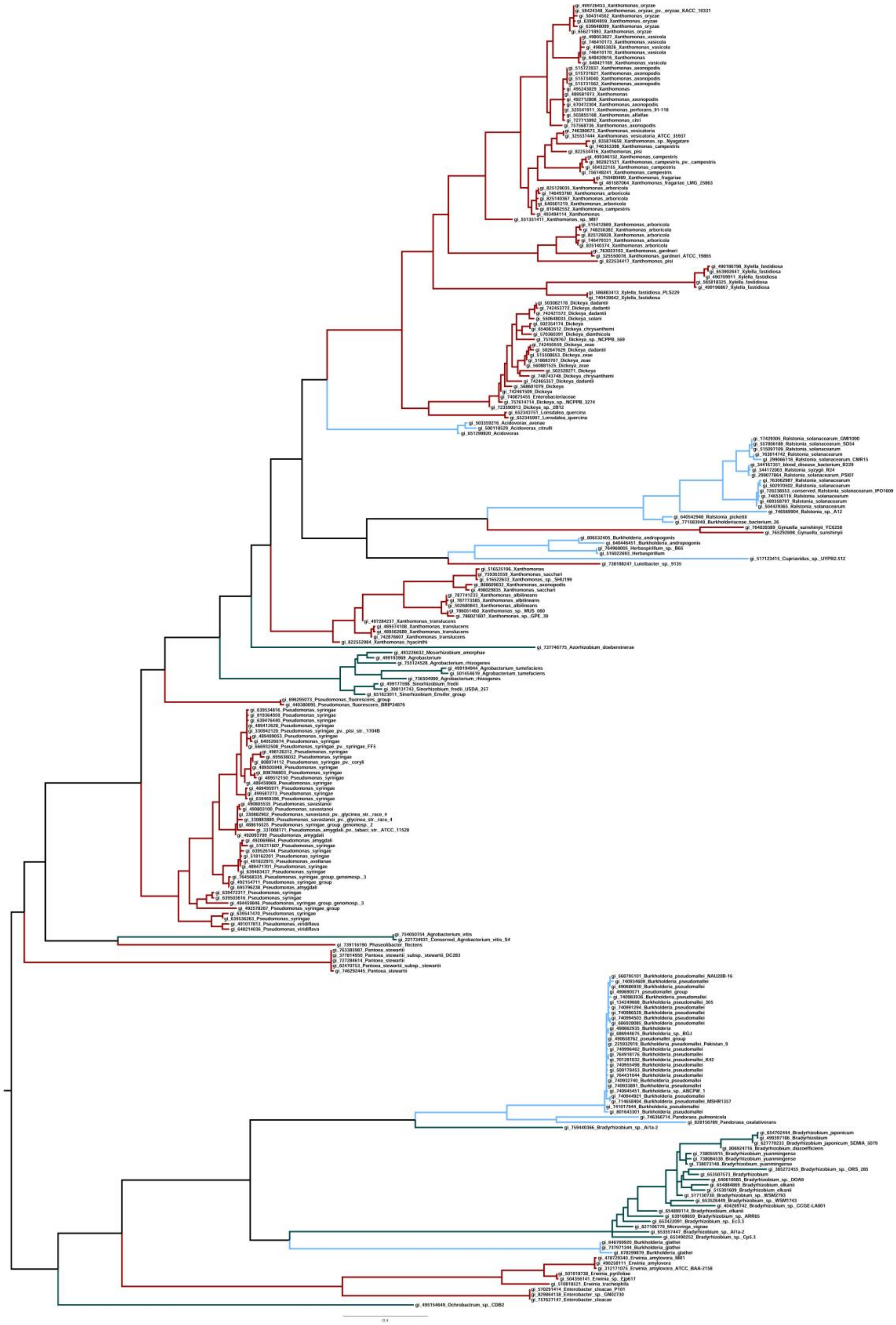

Fig. S16. Maximum likelihood phylogenetic tree of VirK.
